# Herded and hunted goat genomes from the dawn of domestication in the Zagros Mountains

**DOI:** 10.1073/pnas.2100901118

**Published:** 2021-06-07

**Authors:** Kevin G. Daly, Valeria Mattiangeli, Andrew J. Hare, Hossein Davoudi, Homa Fathi, Sanaz Beizaee Doost, Sarieh Amiri, Roya Khazaeli, Delphine Decruyenaere, Jebrael Nokandeh, Tobias Richter, Hojjat Darabi, Peder Mortensen, Alexis Pantos, Lisa Yeomans, Pernille Bangsgaard, Marjan Mashkour, Melinda A. Zeder, Daniel G. Bradley

**Affiliations:** ^a^Smurfit Institute of Genetics, Trinity College Dublin, Dublin 2, Ireland;; ^b^Bioarchaeology Laboratory, Central Laboratory, University of Tehran, 1417466191 Tehran, Iran;; ^c^Archéozoologie, Archéobotanique: Sociétés, Pratiques et Environnements (UMR7209), CNRS, Muséum National d’Histoire Naturelle, 75005 Paris, France;; ^d^National Museum of Iran, 1136917111 Tehran, Iran;; ^e^Research Institute of Cultural Heritage and Tourism Organization of Iran, 1136917111 Tehran, Iran;; ^f^Centre for the Study of Early Agricultural Societies, Department of Cross-Cultural and Regional Studies, University of Copenhagen, 2300 Copenhagen, Denmark;; ^g^Department of Archaeology, Razi University, 6714414971 Kermanshah, Iran;; ^h^Globe Institute, University of Copenhagen, 1165 Copenhagen, Denmark;; ^i^Osteology Department, National Museum of Iran, 1136917111 Tehran, Iran;; ^j^Department of Anthropology, National Museum of Natural History, Smithsonian Institution, Washington, DC 20560

**Keywords:** caprine, ancient DNA, Neolithic, domestication, archaeozoology

## Abstract

The Aceramic Neolithic (∼9600 to 7000 cal BC) period in the Zagros Mountains, western Iran, provides some of the earliest archaeological evidence of goat (*Capra hircus*) management and husbandry by circa 8200 cal BC, with detectable morphological change appearing ∼1,000 y later. To examine the genomic imprint of initial management and its implications for the goat domestication process, we analyzed 14 novel nuclear genomes (mean coverage 1.13X) and 32 mitochondrial (mtDNA) genomes (mean coverage 143X) from two such sites, Ganj Dareh and Tepe Abdul Hosein. These genomes show two distinct clusters: those with domestic affinity and a minority group with stronger wild affinity, indicating that managed goats were genetically distinct from wild goats at this early horizon. This genetic duality, the presence of long runs of homozygosity, shared ancestry with later Neolithic populations, a sex bias in archaeozoological remains, and demographic profiles from across all layers of Ganj Dareh support management of genetically domestic goat by circa 8200 cal BC, and represent the oldest to-this-date reported livestock genomes. In these sites a combination of high autosomal and mtDNA diversity, contrasting limited Y chromosomal lineage diversity, an absence of reported selection signatures for pigmentation, and the wild morphology of bone remains illustrates domestication as an extended process lacking a strong initial bottleneck, beginning with spatial control, demographic manipulation via biased male culling, captive breeding, and subsequently phenotypic and genomic selection.

## Significance

Goats were among the first domestic animals and today are an important livestock species; archaeozoological evidence from the Zagros Mountains of western Iran indicates that goats were managed by the late ninth/early eighth millennium. We assess goat assemblages from Ganj Dareh and Tepe Abdul Hosein, two Aceramic Neolithic Zagros sites, using complementary archaeozoological and archaeogenomic approaches. Nuclear and mitochondrial genomes indicate that these goats were genetically diverse and ancestral to later domestic goats and already distinct from wild goats. Demographic profiles from bone remains, differential diversity patterns of uniparental markers, and presence of long runs of homozygosity reveal the practicing and consequences of management, thus expanding our understanding of the beginnings of animal husbandry.

The initial domestication of southwest Asian crops and livestock species unfolded across the Fertile Crescent after the end of the Younger Dryas climatic downturn circa 9600 BC and coalesced into fully integrated agricultural economies by 7500 cal BC (1). Until recently, the western part of this region was cast as the epicenter of this transition, while the Zagros Mountains, at the eastern end of the Fertile Crescent, was considered a backwater, slow to receive and embrace domesticates and food-producing technologies from farther west (2, 3). However, this region of western Iran has been recently postulated as a primary center for the domestication of a number of plant and animal species (4), including barley (5, 6), possibly emmer wheat (7), several pulse species (8), and most notably, goats (9). This latter hypothesis has been supported by ancient genomic data, which indicate that the eastern Fertile Crescent was one of three regions key in shaping the Neolithic goat gene pool (10).

Here we explore the transition from the hunting of wild bezoar goats (*Capra aegagrus*) to their initial management and subsequent domestication in the eastern Fertile Crescent by combining ancient genome sequencing and archaeozoological evidence from Ganj Dareh and Tepe Abdul Hosein, two sites in the central Zagros dating to the early or Aceramic Neolithic (AN) circa 8000 cal BC. These genomic data present a singular opportunity to deepen our understanding of the consequences of goat management at the dawn of domestication.

## Ganj Dareh and Tepe Abdul Hosein

**Fig. 1.**
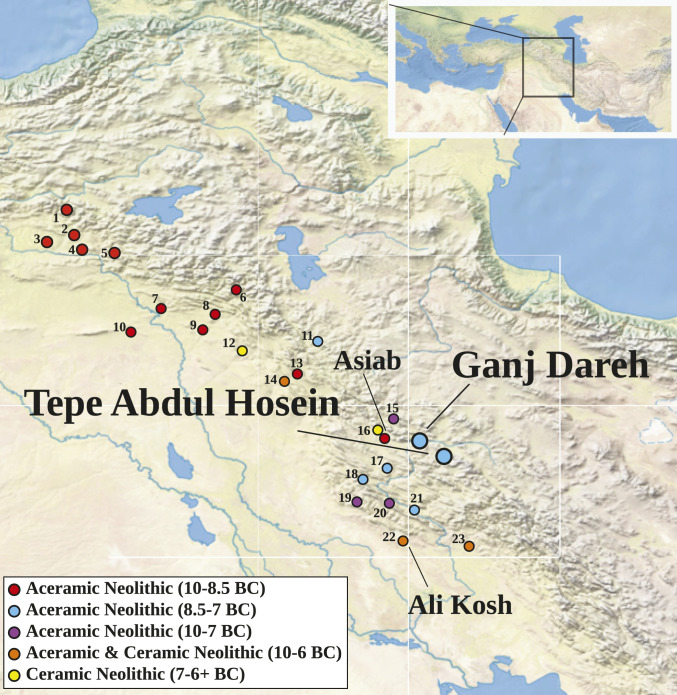
Map showing selected archaeological sites of the Zagros and eastern Fertile Crescent, with sampled sites enlarged and those mentioned in the text labeled. Site key presented in *SI Appendix*, Table S1. *Inset* map shows broader geographic context.

Ganj Dareh (*SI Appendix*, Fig. S1) is located in a side valley of the Gamasiab river valley near Harsin in western highland Iran at an elevation of ∼1,400 m above mean sea-level (AMSL) (Fig. 1). Excavations encountered five major occupation levels (levels A to E) (11). The upper four levels contained architecture composed of rectangular structures with thick mud-brick walls, while the lowest, level E, consisted primarily of pits; the stratigraphy was recently clarified by an Iranian–Danish collaboration (12). Tepe Abdul Hosein (*SI Appendix*, Fig. S2) is located in the highland Zagros (circa 1,830 m AMSL) ∼75 km southeast of Ganj Dareh. A single season of excavation at the site encountered lower levels that, as at Ganj Dareh, consisted of ashy deposits, with upper levels containing more substantial mud-brick architecture (13). At both sites, excavations have recovered large assemblages of animal remains dominated by caprines, a pattern typical of archaeological sites in the Zagros stretching from the Middle Paleolithic to after the Neolithic (14, 15).

Five accelerator mass spectrometry (AMS) radiocarbon dates (*SI Appendix*, Fig. S3 and Table S2), with other recent direct AMS radiocarbon dates from goat bones remains, point to an occupation of Ganj Dareh lasting several hundred years in the late ninth/early eighth millennium BC (8200 to 7600 cal BC, 1-σ range) (12, 15), with roughly contemporaneous habitation at Tepe Abdul Hosein (16). Together with a number of other contemporary settlements with similar architectural and material culture assemblages, these formed a regional network of interacting communities in the Zagros during the eighth millennium BC (Fig. 1) (17). This cultural nexus was preceded by settlements with shorter occupational episodes that proliferated across the region at the beginning of the Early Holocene (circa 9700 to 8500 BC).

## Archaeozoological Evidence of Goat Domestication in the Central Zagros

Previously published archaeozoological analyses, along with new data presented here, provide an outline of the process of goat domestication in the central Zagros. With their large scimitar-shaped horns and large body size, goats from archaeological sites dating to the Late Pleistocene and the earliest phases of the Early Holocene bear the morphological features of wild bezoars. The demographic profile of these goats is consistent with hunting strategies aimed at maximizing meat returns by focusing on large adult male animals. For example, nearly 70% of the goats from the Early Holocene site of Asiab (9750 to 9300 cal BC) (12, 15) were older than 4 y of age when killed (*SI Appendix*, Table S3), with an emphasis on prime age animals indicated for both males and females (*SI Appendix*, Table S4). Moreover, a preference for adult males is evidenced by a 0.67:1 female-to-male ratio, a pattern even more strongly expressed at earlier Paleolithic sites in the region (*SI Appendix*, Table S5).

More than 1,000 y after Asiab was abandoned, Ganj Dareh and Tepe Abdul Hosein goats still show no sign of the smaller twisted horns (*SI Appendix*, Fig. S4) and reduced body size once held to be markers of initial domestication (2, 18–22). The demographic profile of these goats, however, strongly suggests that they were managed animals (14, 15, 23, 24). Management strategies practiced by present-day herders in the region seeking to maximize herd security and growth cull the majority of males between the ages of about 18 mo to 2 y, reserving a few males as breeding stock. Slaughter of females is delayed until they pass peak reproductive years (25–27). Herd management should therefore be reflected in archaeological assemblages composed of the bones of young males and older adult females. Moreover, assemblages of managed goats are expected to show a bias toward females due to the better preservation and chance of recovery of the fused bones of adult females compared to the more friable unfused bones of young males.

**Fig. 2.**
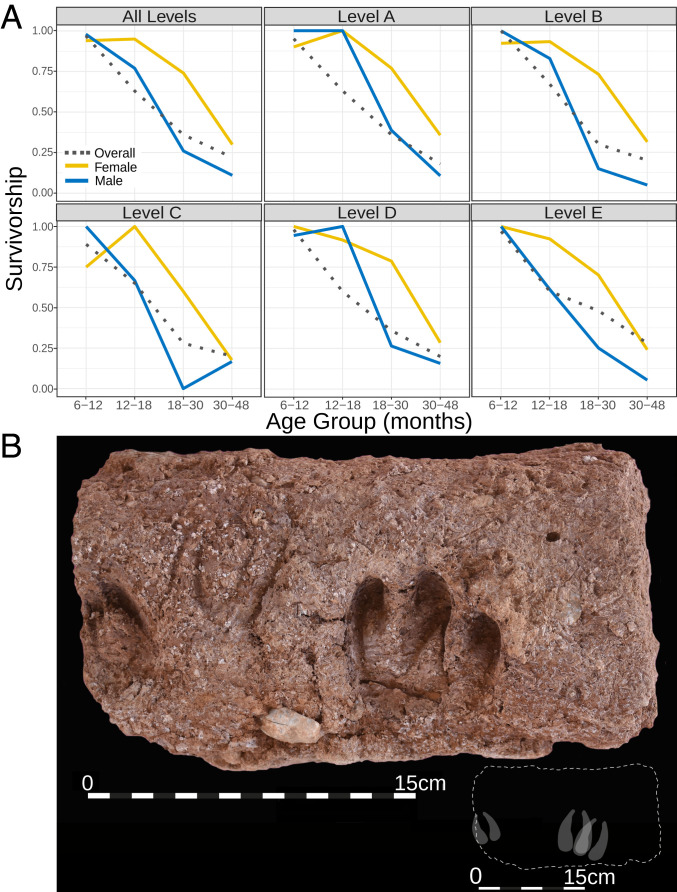
Archaeozoological evidence for goat management at Ganj Dareh. (*A*) Overall and sex-specific combined survivorship curves of goat samples from Ganj Dareh Smith excavation assemblage, showing proportion of animals surviving beyond each age group across all levels and each level individually. The overall curves include material that could not be sexed and are not included in the sex-specific curves. (*B*) Hoofprints in mud-brick at Ganj Dareh (sample 270, context 2033, lowest level of collapse from Smith excavation); *Inset* displays likely individual hoof impressions.

At Ganj Dareh, goat remains recovered from both earlier (14, 15, 23, 24) and recent excavations analyzed by L.Y. and P.B. show a strong emphasis on the harvest of younger animals, with only around 20% surviving beyond 4 y of age (Fig. 2*A* and *SI Appendix*, Table S3). This demographic profile stands in contrast to that of Ganj Dareh sheep, which shows an emphasis on prime age animals (*SI Appendix*, Table S3), suggesting that sheep remained a wild, hunted resource, and representing just 10% of identified caprine remains (24). The age profile of the goats from Tepe Abdul Hosein also shows a small proportion of animals surviving beyond 4 y of age (*SI Appendix*, Table S3), albeit with a later drop in survivorship than at Ganj Dareh (18 mo vs. 12 mo). Moreover, sex-specific harvest profiles of Ganj Dareh goats indicate that between 60% and 70% of males from all five building levels had been harvested before they reached 2.5 y of age, while between 60% and 70% of females survived beyond this age (Fig. 2*A* and *SI Appendix*, Fig. S5 and Tables S4 and S6). In addition, females outnumber males in all five levels in ratios that range from 1.11 to 1.86 to 1 (*SI Appendix*, Tables S5 and S7), consistent with management practices from the beginning of the site’s occupation. Future analysis of the Tepe Abdul Hosein assemblage by M.M. and colleagues will determine if sex-specific harvest profiles and sex ratios resemble those from Ganj Dareh, but the general pattern suggests a similar management strategy was employed at both sites.

In addition, goat assemblages from both the previous (28) and more recent excavations yielded significant numbers of bones of perinatal and neonatal caprines (*SI Appendix*, Fig. S6). High rates of spontaneous abortion and neonatal mortality in managed animals are thought to be linked to the presence of pregnant females and newborn animals close to or within settlements, early management practices, and the rise of zoonotic diseases common in domestic herds, such as brucellosis (29). Evidence of herbivore penning and dung burning at the Zagros sites of Sheikh-e Abad and Jani point to caprine management being practiced across the region during this period (30, 31). The discovery of hoofprints in a mud-brick recovered from Ganj Dareh level D during the early excavations (32), as well as further set of three hoofprints on a brick discovered during the 2017 excavations (Fig. 2*B*), provide an additional indication that Ganj Dareh inhabitants managed goats.

The first detectable morphological change in goats is seen in the site of Ali Kosh in lowland southwest Iran well outside the range of wild goats (Fig. 1) and occupied across the Aceramic–Ceramic Neolithic transition circa 7500 to 6500 BC (33). Demographic profiles from the basal levels of the site are consistent with management in their emphasis on young male harvest and prolonged female survivorship, and a sex ratio strongly biased toward females (*SI Appendix*, Tables S3 and S5). Over the site’s 1,000-y occupation, subtle changes in the size and shape of goat horn cores from the wild to the domestic morphotypes provide the earliest evidence of morphological change in animals undergoing domestication (34). Reduction in body size in Zagros goats is not yet seen until the Ceramic Neolithic (CN, circa 7000 BC) (*SI Appendix*, Fig. S7), although Holocene climate change may have also contributed to the reduction of goat body size over time (15).

## Genomic Insight into Goat Domestication in the Central Zagros

Genomic data from the goats of Ganj Dareh and Tepe Abdul Hosein thus present a singular opportunity to deepen our understanding of the consequences of goat management at the dawn of domestication: prior to detectable morphological change, but after the onset of culling practices consistent with management. Screening of archaeological bones from Ganj Dareh and Tepe Abdul Hosein for preserved goat DNA resulted in 7 samples with surviving DNA from Tepe Abdul Hosein and 25 from across all of Ganj Dareh’s levels (*SI Appendix*, Table S8), with moderate endogenous DNA levels at both sites (mean 15.4% and 9.9%, respectively) showing residual ancient damage after Uracil–DNA–glycosylase treatment (*SI Appendix*, Fig. S8) and low contamination (0 to 1.13%) (*SI Appendix*, Table S9). For further genome sequencing, we targeted those best preserved and produced 10 nuclear genomes from Ganj Dareh (median of 1.3X) and 5 from Tepe Abdul Hosein [median 0.24X; including one published previously (10)] (*SI Appendix*, Tables S8 and S10). Remaining samples were enriched for mitochondrial DNA (mtDNA), giving a total of 25 mtDNA chromosomes from Ganj Dareh and 8 from Tepe Abdul Hosein (*SI Appendix*, Table S11).

## AN Zagros Goat Form Distinct Wild-Affinity and Domestic-Affinity Clusters

**Fig. 3.**
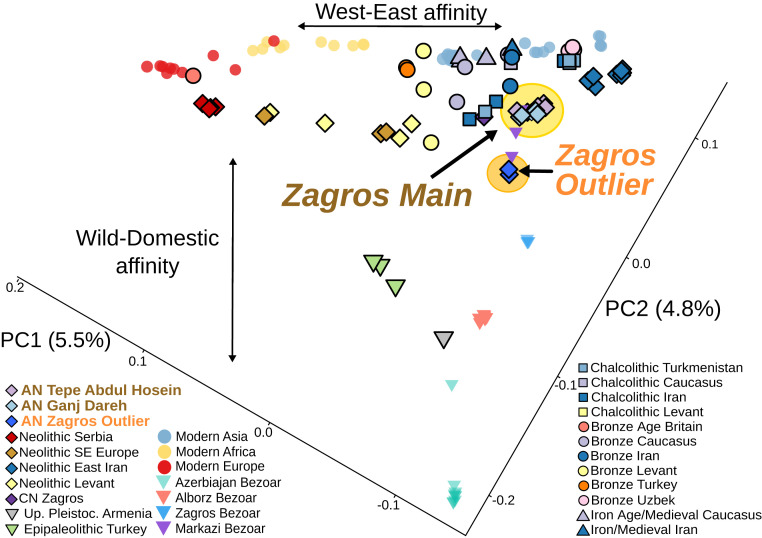
Principal components analysis of wild and domestic goat through time. Variation (%) explained by each PC is indicated in parentheses. Two clusters are observed from the AN Zagros goat, a large group with domestic affinity and a small group with wild affinity.

These genomic data allowed us to assess how early herds relate to wild and later domestic goats. We coanalyzed modern (35–37) and ancient (10) goat genomes (*SI Appendix*, Table S12 and S13) to place these AN Zagros sites in context. Plotting the first two principal components (PC) (Fig. 3), constructed using genotype likelihoods (38), shows a clear linear gradation distinguishing wild modern bezoar ibex from domestic goat. The closest clearly unadmixed modern wild population to domestic goats are from the Zagros Mountains (“Zagros Bezoar”) (*SI Appendix*, *Supplementary Text* and Fig. S9). An orthogonal trend discriminates East–West or Asian–African–European variation. The majority of goat genomes from Tepe Abdul Hosein and Ganj Dareh fall in a single group (“Zagros Main”), close to ancient and modern domestic goats from central and eastern Asia, which is also reflected in affinities deduced from outgroup *f*_*3*_ statistics (*SI Appendix*, Fig. S10). These have affinity to other eastern ancient and modern goat genomes, including later CN populations circa 6000 cal BC, evidenced by a bias in derived allele sharing (39) measured using *D* statistics (*D* ranging from 0.045 to 0.094, *z*-scores > 8) (*SI Appendix*, Fig. S11). Thus, the east–west distinction between goat populations emerges early, likely reflecting the differentiating effects of separate inputs from regional wild populations (10, 37).

A second cluster (“Zagros Outlier”), composed of two genomes (Abdul4 and Ganjdareh35), plots nearby the Zagros Main group but further along the gradation toward wild variation. We explicitly tested for greater wild affinity in Zagros Outlier using the ancient predomestic Armenian bezoar Hovk1 (*SI Appendix*, Table S12) and *D* statistics. Computing *D* (H1, H2; Hovk1, sheep) for pairwise combinations of Zagros Main and Zagros Outlier, we found the majority of tests (21 of 26) with a Zagros Outlier genome show a significant excess of derived alleles shared with the ancient wild Hovk1 (*SI Appendix*, Fig. S12). In contrast, equivalent tests with pairs of Zagros Main genomes are largely not significant (56 of 78). This greater affinity of Zagros Outlier genomes with wild populations is mirrored in ancestry modeling (*SI Appendix*, Fig. S9); furthermore, additional *D* statistics show that Zagros Main goats, but not Zagros Outlier goats, share more derived alleles with Neolithic Serbian domesticates than with ancient wilds (*SI Appendix*, Fig. S13). Together, these analyses indicate that the goat of the AN Zagros formed two distinct genetic clusters, distinguished by their wild and domestic affinities.

These results suggest that Zagros Main genomes derive from herded stock and that Zagros Outlier genomes are hunted from a wild population. Alternatively, Zagros Outlier could represent hybrids between the Zagros Main group and wild bezoar, the result of incidental gene flow or active restocking of herds. To investigate this, we employed *f*-statistics (40) using an iterative qpAdmix approach to model the Zagros Outlier group as deriving from one or many source populations (*SI Appendix*, *Supplementary Text*). Although all two-way mixture models using the Zagros Main cluster and a wild source fit the data, a one-way model with Zagros Outlier forming a clade with modern Zagros Bezoar also fits (*SI Appendix*, Table S14). While reliant on modern wild populations as proxies for past groups, this result supports the Zagros Outlier cluster representing local wild goats, and reflects a persistence of caprine hunting by early herding communities (41), with hunting activities evidenced at Ganj Dareh (24). The managed goats of the central Zagros region were therefore genetically distinct from wild goats by circa 8200 cal BC.

## AN Zagros Goat Are Basal to All Other Domestic Goat

**Fig. 4.**
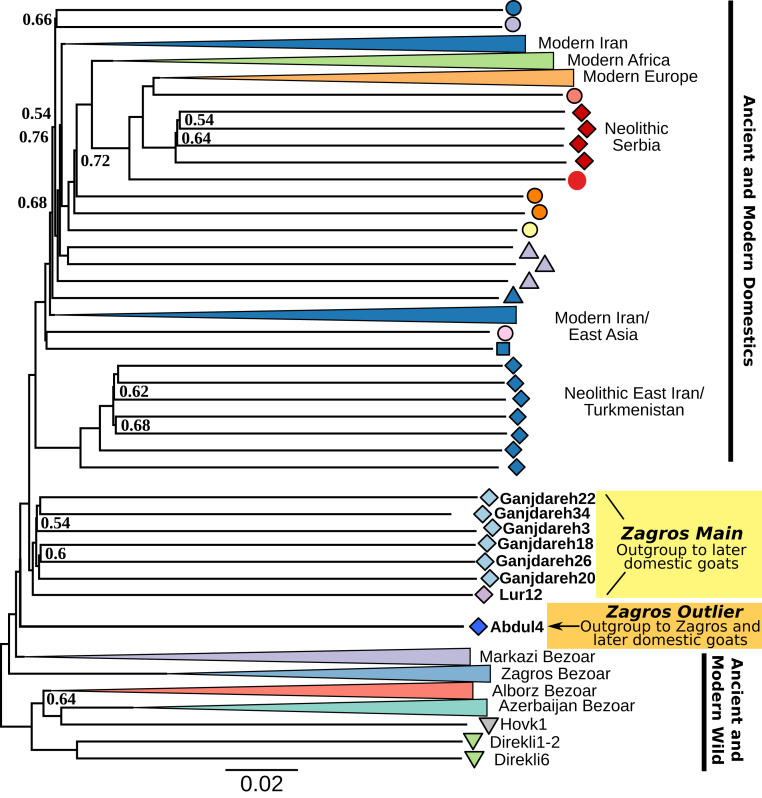
Neighbor-joining tree based on pairwise IBS and with 50 bootstraps and 5-Mb windows, using genomes with ≥1X mean coverage. AN Zagros goats are highlighted, with Zagros Main in yellow and Zagros Outlier basal group in orange. Modern clades are collapsed when possible. Node support values are shown when less than 0.75. The phylogeny demonstrates that the two groups are successive sister groups to later domestic groups, and basal to domestic variation.

**Fig. 5.**
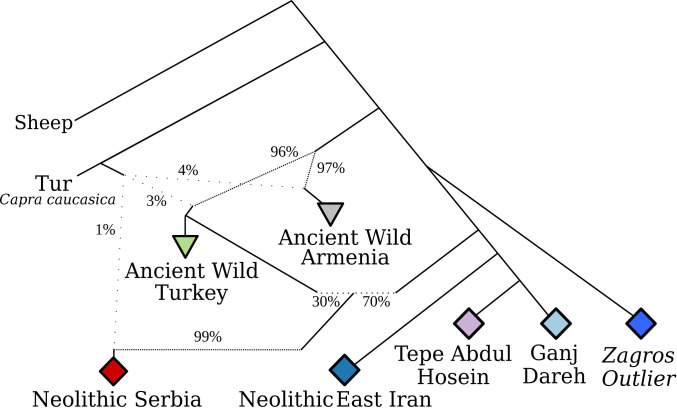
Simplified admixture graph modeling the ancestries of Neolithic and pre-Neolithic goats, with the inclusion of the Tur *C. caucasica* and sheep as an outgroup. Mixing events are represented by dotted lines, with a dash frequency proportional to the contribution of that clade. Drift statistics are present in *SI Appendix*, Fig. S17. Ancient wild turkey = Epipaleolithic Direkli Cave; Ancient Wild Armenia = Upper Pleistocene Hovk 1 Cave. Neolithic East Iran (Tappeh Sang-e Chakhmaq) includes a nearby contemporaneous sample from Monjukli Depe, Turkmenistan (*SI Appendix*, Table S12).

To examine how the two Zagros groups relate to domestic and wild goats in a phylogenetic context, we calculated pairwise identity-by-state (IBS) values and built neighbor-joining trees with, alternately, >1X (Fig. 4 and *SI Appendix*, Fig. S14) and >0.01X genomes (*SI Appendix*, Fig. S15). Both phylogenies place the Zagros Main group as sister with, but basal to, all later domestic goats. Additionally, the Zagros Outlier samples fall as the immediate outgroup to this managed clade, (Fig. 4 and *SI Appendix*, Fig. S14) also observed in Treemix analyses (*SI Appendix*, Fig. S16). We then explicitly modeled the relationships of AN Zagros goats to other populations by exploring admixture graph space (*SI Appendix*, *Supplementary Text*), producing a model without *f*_4_ outlier values (Fig. 5 and *SI Appendix*, Figs. S17 and S18). In this model, Zagros Outlier is an outgroup to other Iranian Neolithic genomes, including those from East Iran/Turkmenistan and the Zagros Main group. Goat from Neolithic Serbia also receive a majority (∼70%) of their ancestry from this clade after their divergence from Zagros Outlier, but derive additional ancestry related to both Epipaleolithic Turkish wild goat (∼30%) and the Caucasian Tur *Capra caucasica* (∼1%) (10, 37). In contrast, the Zagros Main and later East Iranian/Turkmeni Neolithic goat populations—separated by ∼1,800 y and ∼680 km—are modeled as differentiated by drift alone rather than by introgressing wild ancestry, a result supported by qpWave modeling using modern wild goats (*SI Appendix*, Table S15), although *D* statistics do not rule out differential wild affinities among Neolithic Iranian goat (*SI Appendix*, Fig. S19).

Together, these analyses indicate that the managed goats found at the AN sites of Ganj Dareh and Tepe Abdul Hosein are of a type ancestral to domestic goat variation. Wild gene flow contributed to differentiation in later western populations (Fig. 5 and *SI Appendix*, *Supplementary Text*), but our analyses show domestic goats being primarily monophyletic. This monophyly contrast with the deep divergence between Zagros Neolithic human genomes from these sites (16, 42) versus Levantine and Anatolian Neolithic farmers, and points to cultural exchange underlying the initial spread of goat management. It is possible that this managed clade did not originate in the Zagros highlands given evidence of goat management in the central and western Fertile Crescent during the ninth millennium (43, 44). However, the well-documented focus on goat hunting in Late Pleistocene/Early Holocene sites in the central Zagros and the basal position of Zagros Outlier wild goat indicate this region as a focal point for understanding the origin of domestic goat ancestry and management.

## Genomic Indications of Management but Evidence against a Domestication Bottleneck in AN Zagros Goats

**Fig. 6.**
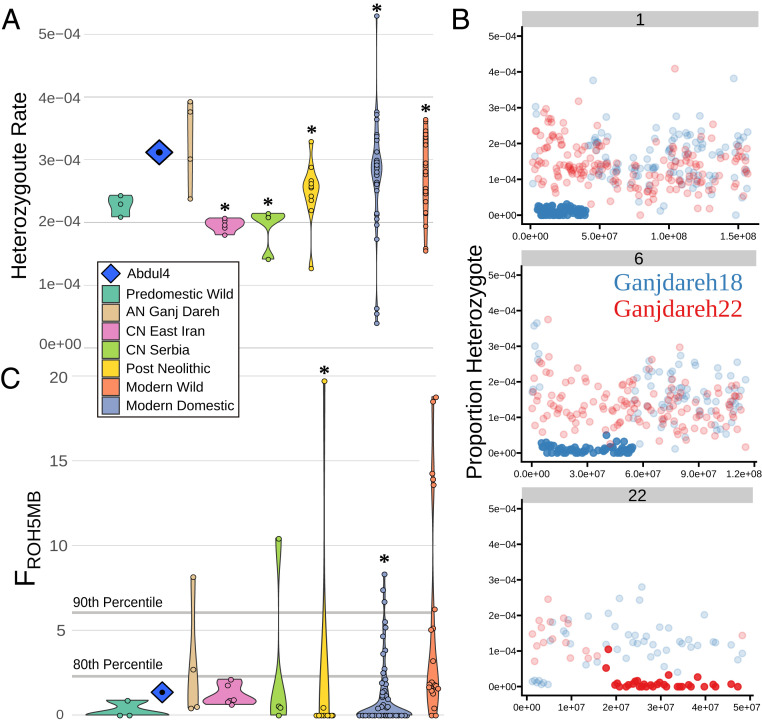
Goat genetic diversity through time. (*A*) Error-corrected transversion heterozygosity as violin plots for modern and ancient genomes downsampled to 2X. A single Zagros Outlier genome, Abdul4, is displayed as a point and symbol. Asterisk (*) indicates significant differences in medians between a group and Ganj Dareh genomes (Wilcoxon rank-sum, *P* value cutoff 0.05). (*B*) Transversion heterozygosity rate in 500-kb windows on chromosomes 1, 6, and 22 for Ganjdareh18 and Ganjdareh22. Windows called as within long ROH regions are displayed in full color. (*C*) *F*_ROH5Mb_ estimates for ancient and modern goats. The 80th and 90th percentiles of *F*_ROH5Mb_ values are indicated. Asterisk (*) indicates difference in group median values with Ganj Dareh. The Ganj Dareh genomes show a combination of high heterozygosity with the presence of long ROH.

We investigated whether an imprint in patterns of diversity from the onset of demographic manipulation and husbandry is detectable in early managed goats. We first assessed transversion heterozygosity in goat through time, limiting our analyses to genomes downsampled to 2X (five genomes from this study) to control for coverage effects and correcting for error (*SI Appendix*, Fig. S20). We found that genetic diversity is high in the early managed Zagros Main goat, declines in CN goats from Serbia and East Iran/Turkmenistan, and rises again in more recent populations (Fig. 6*A*). Similarly, pairwise kinship estimates (45) indicate that our sampled goats from Ganj Dareh and Tepe Abdul Hosein had little close direct kinship (mean π-hat = 0.063) (*SI Appendix*, Fig. S21) despite their general affinity, while the later CN goat from both Serbia and East Iran show higher pairwise kinship (means of 0.234 and 0.242). Together these results indicate that the onset of management was not associated with a dramatic decline in genetic diversity. However, this is apparent circa 2,000 y later in the CN when domestic goats were moved beyond their natural range. The subsequent recovery of heterozygosity in post-Neolithic goats likely reflects the onset of greater interregional gene flow (10, 46–48).

We then assessed the AN Zagros goat genomes for long runs of homozygosity (ROH), which are indicative of kin mating, under the hypothesis that the prevalence of long ROH (≥5 Mb) could be a marker of early animal husbandry. Using male X chromosomes to set a transversion heterozygosity threshold, we observed that all four Zagros Main genomes downsampled to 2X coverage possess at least one ROH ≥ 5 Mb (Fig. 6*B* and *SI Appendix*, Figs. S22–S25), with two of the four samples (Ganjdareh18 and Ganjdareh22) having long ROH (*SI Appendix*, Fig. S26) encompassing notable proportions of their total filtered windows (inbreeding coefficient from ROH regions ≥5 Mb only, *F*_ROH5Mb_; 2.7% and 8.2%), exceeding the 80th percentile of all assessed genomes (Fig. 6*C*). The single tested Zagros Outlier genome (Abdul4) (*SI Appendix*, Fig. S27), and other ancient wild samples, show comparatively low *F*_ROH5Mb_ values (Fig. 6*C*). Modern wild bezoar from Iran show the highest rates of extreme *F*_ROH5Mb_, consistent with documented recent population decline (49) and lower levels of diversity compared to domesticates (36).

The interpretation of ROH patterns in Neolithic goat would be further informed by data from a range of predomestication wild populations. Their frequently fragmented distributions (50), long-distance dispersal ability, and male intrasexual competition for mates (51) may influence ROH distributions, which have been detailed in modern populations elsewhere (52), fully leveraging available genomic data. However, outbred human genomes do not tend to show ROH > 4 Mb in size (53), which is lower than the threshold of ROH reported here; while this should not be overgeneralized due to the potential wild goat-specific population structure, it supports the interpretation of recent inbreeding loops within AN Zagros goat herds. Although our sample size is limited, the data suggest that the onset of goat management may have been accompanied by a greater frequency of inbreeding events, and that long ROH are a possible genomic marker for the early domestication process. The contrast between the heterozygosity and long ROH prevalence in AN Zagros goat could reflect a crucial phase of management: after the beginnings of caprine husbandry, but prior to their removal beyond their wild range.

## Mitochondrial and Y Chromosome Markers Show Contrasting Diversity Patterns

**Fig. 7.**
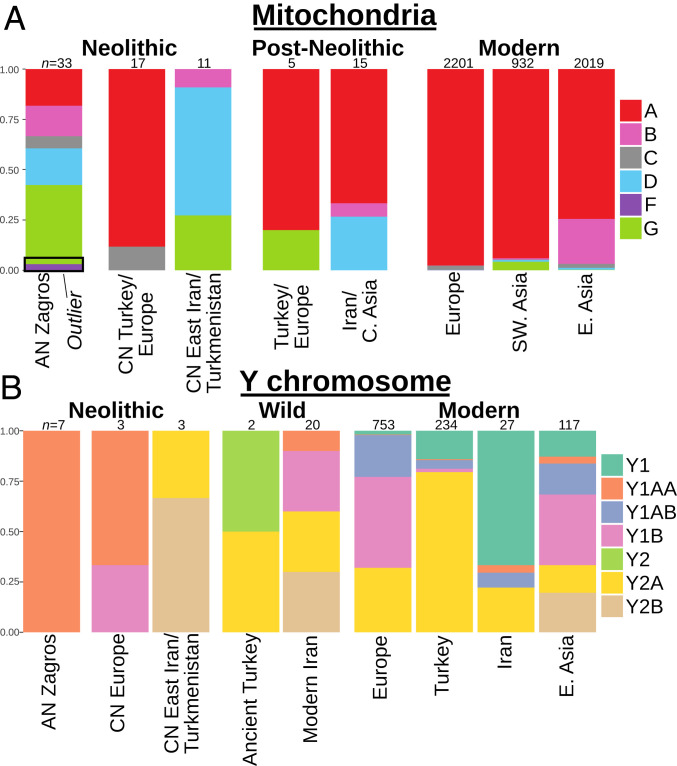
Uniparental haplogroup frequencies for (*A*) mtDNA and (*B*) Y chromosomes. Zagros Outlier mtDNA are indicated by a black box. Frequencies for modern populations were obtained from refs. 54 and 55. Distinct diversity patterns are seen in the AN Zagros goat uniparental markers, with greater diversity observed in the maternal mtDNA pool.

A characteristic of livestock mtDNA diversity is its partitioning into small numbers of distinct clades, or haplogroups, with shallow within-group coalescence. mtDNA of modern goats are overwhelmingly (∼92%) of the A haplogroup (Fig. 7*A*) (54). Haplogroups at minor global frequency today (B, C, D, G, and the primarily wild F) were dominant in specific locales in the later Neolithic, with this geographic structure lost in post-Neolithic periods (10). The two AN Zagros sites (Fig. 7*A* and *SI Appendix*, Fig. S28) show pronounced mtDNA diversity. At Ganj Dareh and throughout its occupation (*SI Appendix*, Table S8), the entire range of haplogroups associated with modern domestic goats are observed, with a similar (*SI Appendix*, Table S16) broad range of haplogroups at Tepe Abdul Hosein. Bayesian analysis indicates that sequences from the two sites have high affinity (*SI Appendix*, Fig. S29), in many instances forming intersite clades that coalesced recently (∼11000 to 8000 BC, 95% highest posterior density estimates). However, within each haplogroup Zagros Main samples form clades with both wild- and domestic-derived sequences (*SI Appendix*, Fig. S28). In comparison, the wild Zagros Outlier samples Abdul4 and Ganjdareh35 have mtDNA (G and F, respectively), which do not form clades with any modern domesticates.

This high matrilineal diversity contrasts sharply with AN Zagros patrilines. We constructed maximum-likelihood trees from Y chromosome variants with and without transitions (*SI Appendix*, Figs. S30 and S31), and assigned haplogroups based on modern sequences (Fig. 7*B* and *SI Appendix*, *Supplementary Text* and Table S17). All sequences from the AN Zagros fall within a single lineage, Y1AA, now at a low frequency in wild and domestic Iranian goats and absent from sampled Zagros wild males (55). In comparison, sampling of small numbers in predomestic goats from the Taurus Mountains show distinct haplotypes, while males from CN Serbia and CN East Iran display two different lineages (Fig. 7*B*).

This contrast between patrilineal and matrilineal markers is striking, and mirrors the differential survivorship between males and female goats at Ganj Dareh (Fig. 2*A*). AN Zagros goat harbor all mtDNA haplogroups observed in modern domestic goat, a diversity spectrum not observed in any modern or ancient population (10, 54), and have high intragroup diversity levels as measured by average number of pairwise differences (*SI Appendix*, Table S18). In comparison, their Y chromosome lineages are restricted to a single haplogroup, with later Neolithic populations adjacent to the Fertile Crescent showing greater lineage diversity (Fig. 7*B*). This disparity is consistent with the onset of goat husbandry with its inherent bias in survival between sexes and a marked restriction in breeding male population size (Fig. 2*A* and *SI Appendix*, Tables S4 and S6).

## Absence of Selection Signals or Morphological Change in Early Managed Goat

Previous work has indicated evidence, from an *F*_*ST*_ outlier scan, of selection in goat populations in Serbia and East Iran by 6000 cal BC, in particular at pigmentation-associated genes (10), with two loci encompassing the *STIM1-RRM1* and *MUC6* genes also under selection during the past 8,000 y (37). We replicated this analysis using the Zagros Main genomes (10) and identify 45 genomic regions overlapping or neighboring 48 annotated genes (*SI Appendix*, Table S19), but found no evidence for pigmentation or domestication-associated genes, or evidence of gene ontology enrichment. Similarly, an alternate approach examined genes in the top 0.1% of population branch statistic windows, but these showed limited evidence of functional enrichment (solely conjugated hyperbilirubinemia, *P*_adj_ = 0.0416). Additionally, we did not detect the derived *MUC6* haplotype in the genomes of AN Zagros goat (*SI Appendix*, Fig. S32), consistent with a hypothesis of its introduction via introgression to later populations (37), but did find the derived *STIM1-RRM1* haplotype at frequencies similar to CN Serbia (∼19 to 21%), and in a single Zagros Outlier genome. As *STIM1* may have a role in anxiety-like behavior and learning (37, 56), managed goats derived from a wild population that may have already carried the standing variation which would be later selected during the domestication process.

Together, these results indicate that the selection events that define the later evolution of goats were absent in the first managed herds circa 8000 BC. The lack of signals of later selective sweeps or functional enrichment is concordant with the wild-like morphology and horn shapes of goats at both sites (*SI Appendix*, Fig. S4), indicating that the phenotypic and genetic changes commonly associated with domestication had not yet occurred in the managed goat of the central Zagros AN. Positive selection for traits during early domestication may therefore have required additional perturbations other than initial demographic manipulation by humans, such as the cessation of wild gene flow from the area of their initial recruitment, increased herd size, gene flow from diverged wild populations or related species (37), or a change in the management practices of early farmers.

## Conclusion

We find evidence for a duality in goat genomes in the early Zagros Neolithic that likely reflects diverged hunted and herded animals. Thus, both archaeogenomic and archaeozoological methods reveal a majority of Ganj Dareh and Tepe Abdul Hosein goats that were genetically diverged but morphologically indistinguishable from wild bezoar ibex, while being subject to culling practices consistent with animal management. At Ganj Dareh these archaeological signals are clear from the earliest occupation level (Fig. 2*A*) and also at Tepe Abdul Hosein (*SI Appendix*, Table S3), and are complemented by genetic evidence of management with kin matings (i.e., long ROH) (Fig. 6*B*), an ancestral position within domestic goat genetic diversity (Fig. 4), and sex-biased diversity patterns implying strong restriction of the male mating pool (Fig. 7).

Together, these analyses demonstrate that management of a genetically distinct herd was already practiced by the time the sites were established circa 8200 cal BC, and push back the beginning of goat management in the Zagros well into the ninth millennium. The earliest date for caprine management is bracketed by demographic evidence for exclusively wild goat exploitation at the earlier site of Asiab (12, 15), placing its onset within 9300 to 8200 cal BC. The close affinity of the domestic-like Zagros Main goat with the wild-like Zagros Outlier, their basal position with goat variation, and the breadth of mtDNA haplogroups (Fig. 7*A*) are suggestive of the region being a major source of domestic goat variation as established by ∼8200 cal BC. The high matrilineal haplogroup spectrum fits with scenarios preserving genome diversity during early goat management, including recruitment from one or several distinct populations, on-going wild gene flow, or existence within a larger metapopulation distributed among early herding communities (3), likely interacting via trade (1, 57). Within the context of the southwest Asian Neolithic, the Zagros may represent a key pole among interacting early herding communities. Indications of caprine herding in more western Anatolia (43) roughly concurrent with the study period and the genomic evidence of Anatolian wild goat recruitment (Fig. 5) (10) fit with a more diffuse concept of the establishment of the domestic gene pool in the Zagros and its surrounding regions, rather than a discrete event in time and space. Observations of persistent modification to livestock populations in cattle (58) and pig (59) suggest that this was not limited to goats, but was a feature of Neolithic farming practices. The occurrence of the *STIM1-RRM1*–selected variant—with its possible neuronal and behavioral implications (56)—in Neolithic Zagros goat also hints that managed goats derived substantial ancestry from a population that, unlike tested wild bezoar (37), possessed variation selected for later in their domestication history.

Our combined archaeozoological and genetic analyses—demonstrated as complementary leading-edge markers for early management—show that the goat assemblages from Ganj Dareh and Tepe Abdul Hosein capture a key phase of domestication: after the initiation of management practices, which left their genomic imprint on early goat herds, but before the selective pressures responsible for their distinctive traits, such as altered body size and changes in horn morphology. Together with contemporaneous indications of goat, sheep, and pig management in the Upper Euphrates and Tigris valleys (43), the Levant (60), and Anatolia (61, 62), future research must examine earlier sites within the Zagros for initial signals of the beginnings of animal management, particularly those founded at the dawn of the Holocene (Fig. 1). Here, we show that the genetic domestication of goats was well underway by 8200 cal BC in the Zagros region, and that the goats of Ganj Dareh and Tepe Abdul Hosein thus represent the oldest yet-reported livestock genomes.

## Materials and Methods

A full description of the materials and methods used are provided in *SI Appendix*, *Supplementary Text*. Briefly, species identification of caprine were made following criteria in refs. 63–65, sexing criteria was derived from refs. 66 and 67, and metric followed ref. 68. We extracted DNA using a dilute bleach wash and proteinase digestion (69), treated DNA with USER enzyme (70), and constructed dsDNA libraries (71). Sequencing was performed on Illumina HiSeq 2500 (1 × 100 bp) or NovaSeq (2 × 50 bp), and reads aligned to the ARS1 goat reference (72) using bwa aln (73) relaxing parameters (74). Transversion biallelic sites were determined from modern wild and domestic genomes (*SI Appendix*, Table S13), and pseudohaploid genotypes for ancient samples (reported here and in ref. 10) generated by random read sampling. Pseudohaploid genotypes (56,682 sites) were used for Treemix, kinship (45), outgroup *f*_*3*_ (pooled wild goat as outgroup), and Admixtools analyses (qpGraph, qpWave, qpAdm) (40). *D* statistics (39), IBS, heterozygosity and error estimation, population branch statistic, and *F*_ST_ outlier analyses were performed using the ANGSD toolkit (75) with transversion variants only. PCAngsd was used for principal components analysis and ancestry estimation (38). Providence and groupings used for each ancient sample are displayed in *SI Appendix*, Table S12. Reads were aligned using bwa to the goat mtDNA reference (NC_005044.2), before realignment to a circularized haplogroup representative, and consensus sequences generated using ANGSD; mtDNA were aligned using MUSCLE (76). Contamination was determined by heteroplasmic rate at known mtDNA variant positions (54). Samples were aligned to Y contigs (NW_017189563.1, NW_017189685.1, NW_017189885.1), transversion variants ascertained in moderns and called in ancient samples, and fasta sequences generated after quality control. mtDNA diversity and *F*_ST_ was determined with Arlequin (77). Uniparental trees were built with phyML3.0 (78) and BEAST2 (79).

For long ROH (≥5 Mb), we followed an observation-based approach by calculating the rate of transversion heterozygous sites in 500-kbp nonoverlapping windows using bcftools v1.5, downsampling genomes to 2X to control for varying coverage. Windows (filtered for total called sites >50 kbp) were assessed in sliding groups of 10, one 500-kbp window steps, to identify putative long ROH (≥5 Mb), using a threshold calculated from the nonpseudoautosomal region of 2X-downsampled male X chromosomes to account for error (mean plus 2 SDs, 5.66 × 10^−5^ transversion heterozygous sites per base pair). If at least 9 of the 10 500-kbp windows were below this threshold, allowing for 1 nonterminal window above the threshold, the group of 10 was assigned as putative long ROH. If the distance from the most-recent window end and the next window start was >10 Mb, the previous windows were discarded and a new group of 10 were assessed. For *F*_ST_ outlier detection, we pooled genomes from the Zagros Main cluster with >2X mean coverage and estimated genetic diversity (θ_Watterson_) and *F*_ST_ with modern bezoar in 50-kbp windows and 10-kbp steps. We filtered for windows showing both high divergence (*F*st ≥ 0.99 quantile) and low genetic diversity compared to modern wilds [log(θ_Zagros_/θ_Wild_) < 0]. For known selected variants (37), we determined the genotype with the highest likelihood using ANGSD. Sequencing reads, bam files, and mtDNA fasta sequences are available at the European Nucleotide Archive under accession no. PRJEB40573.

## Supplementary Material

Supplementary File

Supplementary File

## Data Availability

The data have been deposited in the European Nucleotide Archive, https://www.ebi.ac.uk/ena/browser (accesson no. PRJEB40573). Previously published data were used for this work (10, 35–37, 54).

## References

[1] J. J. Ibáñez, J. González-Urquijo, L. C. Teira-Mayolini, T. Lazuén, The emergence of the Neolithic in the Near East: A protracted and multi-regional model. Quat. Int. 470, 226–252 (2018).

[2] O. Bar-Yosef, R. H. Meadow, “The origins of agriculture in the Near East” in Last Hunters, First Farmers: New Perspectives on the Prehistoric Transition to Agriculture, T. D. Price, A.-B. Gebauer, Eds. (School of American Research Press, 1995), pp. 39–94.

[3] S. K. Kozłowski, O. Aurenche, Territories, Boundaries and Cultures in the Neolithic Near East (BAR, 2005).

[4] K. Roustaei, M. Mashkour, “Foreword” in The Neolithic of the Iranian Plateau: Recent Research and Prospects, SENEPSE., K. Roustaei, M. Mashkour, Eds. (Ex Oriente, 2016), pp. i–vii.

[5] P. L. Morrell, M. T. Clegg, Genetic evidence for a second domestication of barley (Hordeum vulgare) east of the Fertile Crescent. Proc. Natl. Acad. Sci. U.S.A. 104, 3289–3294 (2007). 17360640 10.1073/pnas.0611377104 PMC1805597

[6] A. Pankin, J. Altmüller, C. Becker, M. von Korff, Targeted resequencing reveals genomic signatures of barley domestication. New Phytol. 218, 1247–1259 (2018). 29528492 10.1111/nph.15077 PMC5947139

[7] S. Riehl, M. Zeidi, N. J. Conard, Emergence of agriculture in the foothills of the Zagros Mountains of Iran. Science 341, 65–67 (2013). 23828939 10.1126/science.1236743

[8] M. Savard, M. Nesbitt, M. K. Jones, The role of wild grasses in subsistence and sedentism: New Evidence from the northern Fertile Crescent. World Archaeol. 38, 179–196 (2006).

[9] M. A. Zeder, The origins of agriculture in the Near East. Curr. Anthropol. 52, S221–S235 (2011).

[10] K. G. Daly ., Ancient goat genomes reveal mosaic domestication in the Fertile Crescent. Science 361, 85–88 (2018). 29976826 10.1126/science.aas9411

[11] P. E. L. Smith, Ganj Dareh Tepe. Paéorient 2, 207–209 (1974).

[12] H. Darabi, T. Richter, P. Mortensen, Neolithization process in the central Zagros: Asiab and Ganj Dareh revisited. Documenta Praehistorica 46, 44–57 (2019).

[13] J. Pullar, A. Hastings, R. Hubbard, G. Wilcox, Tepe Abdul Hosein: A Neolithic Site in Western Iran: Excavations 1978 (BAR, 1990).

[14] M. A. Zeder, B. Hesse, The initial domestication of goats (Capra hircus) in the Zagros mountains 10,000 years ago. Science 287, 2254–2257 (2000). 10731145 10.1126/science.287.5461.2254

[15] M. Zeder, “Animal domestication in the Zagros: An update and directions for future research” in Archaeozoology of the Near East VII, E. Vila, L. Gourichon, A. Choyke, H. Buitenhuis, Eds. (Travaux de la Maison de l’Orient et de la Méditerranée, Lyon, 2008), vol. 49, pp. 243–277.

[16] F. Broushaki ., Early Neolithic genomes from the eastern Fertile Crescent. Science 353, 499–503 (2016). 27417496 10.1126/science.aaf7943 PMC5113750

[17] H. Darabi, P. Mortensen, An Introduction to the Neolithic Revolution of the Central Zagros (BAR, Iran, 2015).

[18] C. A. Reed, “A review of the archaeological evidence on animal domestication in the prehistoric Near East” in Prehistoric Investigations in Iraqi Kurdistan, R. J. Braidwood, B. Howe, Eds. (University of Chicago Press, Studies in Ancient Oriental Civilization, 1960), pp. 119–146.

[19] S. Bökönyi, The Animal Remains from Four Sites in the Kermanshah Valley (BAR, Iran, 1977).

[20] H.-P. Uerpmann, Probleme der Neolithisierung des Mittelmeerraums (Reichert, 1979).

[21] R. H. Meadow, “Animal domestication in the Middle East: A view from the eastern margin” in Animals and Archaeology, Volume 3: Early Herders and Their Flocks, J. Clutton-Brock, C. Grigson, Eds. (BAR International Series, BAR, 1984), pp. 309–337.

[22] J. Peters, D. Helmer, A. von den Driesch, M. Saña Segui, Early animal husbandry in the northern Levant. Paéorient 25, 27–48 (1999).

[23] B. C. Hesse, Evidence for Husbandry from the Early Neolithic Site of Ganj Dareh in Western Iran (Columbia University, Ann Arbor, MI, 1978).

[24] B. Hesse, “These are our goats: The origins of herding in west central Iran” in Animals and Archaeology, Volume 3: Early Herders and Their Flocks, J. Clutton-Brock, C. Grigson, Eds. (BAR International Series, BAR, 1984), pp. 243–264.

[25] S. Payne, Kill-off patterns in sheep and goats: The mandibles from Aşvan Kale. Anatolian Studies 23, 281–303 (1973).

[26] J. P. Digard, Techniques des Nomades Baxtyâri d’Iran (Editions de la Maison des sciences de l’homme, Cambridge University Press, Cambridge, 1981).

[27] R. W. Redding, “Decision Making in Subsistence Herding of Sheep and Goats in the Middle East.” PhD thesis, University of Michigan, Ann Arbor, MI (1981).

[28] B. Hesse, Slaughter patterns and domestication: The beginnings of pastoralism in western Iran. Man (Lond.) 17, 403–417 (1982).

[29] G. Fournié, D. U. Pfeiffer, R. Bendrey, Early animal farming and zoonotic disease dynamics: Modelling brucellosis transmission in Neolithic goat populations. R. Soc. Open Sci. 4, 160943 (2017). 28386446 10.1098/rsos.160943 PMC5367282

[30] W. Matthews, “Investigating early Neolithic materials, ecology and sedentism: micromorphology and microstratigraphy” in The Earliest Neolithic of Iran: 2008 Excavations at Sheikh-E Abad and Jani: Central Zagros Archaeological Project, Volume 1, R. Matthews, W. Matthews, Y. Mohammadifar, Eds. (British Institute of Persian Studies, Archaeological Monograph Series, Oxbow Books, 2013), pp. 67–104.

[31] W. Matthews, L.-M. Shillito, S. Elliott, I. D. Bull, J. Williams, “Neolithic lifeways: Microstratigraphic traces within houses, animal pens and settlements” in Early Farmers: The View from Archaeology and Science, Proceedings of the British Academy, A. Whittle, P. Bickle, Eds. (Oxford University Press, 2014), pp. 251–279.

[32] P. E. L. Smith, An interim report on Ganj Dareh Tepe, Iran. Am. J. Archaeol. 82, 537–540 (1978).

[33] H. Darabi, Revisiting stratigraphy of Ali Kosh, Deh Luran plain. Pazhohesh-Ha-Ye Bastanshenasi Iran 8, 27–42 (2018).

[34] F. Hole, K. V. Flannery, J. A. Neely, Prehistory and Human Ecology of the Deh Luran Plain: An Early Village Sequence from Khuzistan (University of Michigan Press, Iran, 1969).

[35] B. Benjelloun .; NextGen Consortium, Characterizing neutral genomic diversity and selection signatures in indigenous populations of Moroccan goats (Capra hircus) using WGS data. Front. Genet. 6, 107 (2015). 25904931 10.3389/fgene.2015.00107 PMC4387958

[36] F. J. Alberto ., Convergent genomic signatures of domestication in sheep and goats. Nat. Commun. 9, 813 (2018). 29511174 10.1038/s41467-018-03206-y PMC5840369

[37] Z. Zheng ., The origin of domestication genes in goats. Sci. Adv. 6, eaaz5216 (2020). 32671210 10.1126/sciadv.aaz5216 PMC7314551

[38] J. Meisner, A. Albrechtsen, Inferring population structure and admixture proportions in low-depth NGS data. Genetics 210, 719–731 (2018). 30131346 10.1534/genetics.118.301336 PMC6216594

[39] R. E. Green ., A draft sequence of the Neandertal genome. Science 328, 710–722 (2010). 20448178 10.1126/science.1188021 PMC5100745

[40] N. Patterson ., Ancient admixture in human history. Genetics 192, 1065–1093 (2012). 22960212 10.1534/genetics.112.145037 PMC3522152

[41] M. C. Stiner ., A forager-herder trade-off, from broad-spectrum hunting to sheep management at Aşıklı Höyük, Turkey. Proc. Natl. Acad. Sci. U.S.A. 111, 8404–8409 (2014). 24778242 10.1073/pnas.1322723111 PMC4060719

[42] M. Gallego-Llorente ., The genetics of an early Neolithic pastoralist from the Zagros, Iran. Sci. Rep. 6, 31326 (2016). 27502179 10.1038/srep31326 PMC4977546

[43] D. Helmer, L. Gourichon, H. Monchot, J. Peters, M. S. Seguí, “The upper Euphrates-Tigris basin: Cradle of agro-pastoralism?” in First Steps of Animal Domestication: New Archaeozoological Approaches, J. Peters, A. von den Driesch, D. Helmer, Eds. (Oxbow Books, 2005), pp. 86–124.

[44] C. Makarewicz, N. Tuross, Finding fodder and tracking transhumance: Isotopic detection of goat domestication processes in the Near East. Curr. Anthropol. 53, 495–505 (2012).

[45] M. Lipatov, K. Sanjeev, R. Patro, K. Veeramah, Maximum likelihood estimation of biological relatedness from low coverage sequencing data. bioRxiv [Preprint] (2015). 10.1101/023374 (Accessed 15 February 2016).

[46] L. Colli .; AdaptMap Consortium, Genome-wide SNP profiling of worldwide goat populations reveals strong partitioning of diversity and highlights post-domestication migration routes. Genet. Sel. Evol. 50, 58 (2018). 30449284 10.1186/s12711-018-0422-x PMC6240949

[47] Y. Cai ., Ancient genomes reveal the evolutionary history and origin of cashmere producing goats in China. Mol. Biol. Evol. 37, 2099–2109 (2020). 32324877 10.1093/molbev/msaa103 PMC7306693

[48] T. R. Hermes ., High mitochondrial diversity of domesticated goats persisted among Bronze and Iron Age pastoralists in the Inner Asian Mountain Corridor. PLoS One 15, e0233333 (2020). 32437372 10.1371/journal.pone.0233333 PMC7241827

[49] D. M. Shackleton, Wild Sheep and Goats and Their Relatives: Status Survey and Conservation Action Plan for Caprinae (IUCN, 1997).

[50] T. Kuemmerle ., Identifying priority areas for restoring mountain ungulates in the Caucasus ecoregion. Conservat. Sci. Prac. 2, e276 (2020).

[51] O. Macar, B. Gürkan, Observations on behavior of wild goat (Capra aegagrus, Erxleben 1777). Hacettepe J. Biol. Chem. 37, 13–21 (2009).

[52] C. Grossen, F. Guillaume, L. F. Keller, D. Croll, Purging of highly deleterious mutations through severe bottlenecks in Alpine ibex. Nat. Commun. 11, 1001 (2020). 32081890 10.1038/s41467-020-14803-1 PMC7035315

[53] M. Kirin ., Genomic runs of homozygosity record population history and consanguinity. PLoS One 5, e13996 (2010). 21085596 10.1371/journal.pone.0013996 PMC2981575

[54] L. Colli ., Whole mitochondrial genomes unveil the impact of domestication on goat matrilineal variability. BMC Genomics 16, 1115 (2015). 26714643 10.1186/s12864-015-2342-2 PMC4696231

[55] I. J. Nijman ., Phylogeny and distribution of Y-chromosomal haplotypes in domestic, ancient and wild goats. bioRxiv [Preprint] (2020). https://www.biorxiv.org/content/10.1101/2020.02.17.952051v1 (Accessed 18 August 2020).

[56] Ł. Majewski ., Overexpression of STIM1 in neurons in mouse brain improves contextual learning and impairs long-term depression. Biochim. Biophys. Acta Mol. Cell Res. 1864, 1071–1087 (2017). 27913207 10.1016/j.bbamcr.2016.11.025

[57] O. Barge ., Diffusion of Anatolian and Caucasian obsidian in the Zagros Mountains and the highlands of Iran: Elements of explanation in “least cost path” models. Quat. Int. 467, 297–322 (2018).

[58] M. P. Verdugo ., Ancient cattle genomics, origins, and rapid turnover in the Fertile Crescent. Science 365, 173–176 (2019). 31296769 10.1126/science.aav1002

[59] L. A. F. Frantz ., Ancient pigs reveal a near-complete genomic turnover following their introduction to Europe. Proc. Natl. Acad. Sci. U.S.A. 116, 17231–17238 (2019). 31405970 10.1073/pnas.1901169116 PMC6717267

[60] N. D. Munro ., The Emergence of animal management in the southern Levant. Sci. Rep. 8, 9279 (2018). 29915348 10.1038/s41598-018-27647-z PMC6006362

[61] A. Ervynck, K. Dobney, H. Hongo, R. Meadow, Born free? New evidence for the status of Sus scrofa at Neolithic Çayönü Tepesi (southeastern Anatolia, Turkey). paleo 27, 47–73 (2001).

[62] J. T. Abell ., Urine salts elucidate Early Neolithic animal management at Aşıklı Höyük, Turkey. Sci. Adv. 5, eaaw0038 (2019). 31001590 10.1126/sciadv.aaw0038 PMC6469938

[63] M. A. Zeder, H. A. Lapham, Assessing the reliability of criteria used to identify postcranial bones in sheep, Ovis, and goats, Capra. J. Archaeol. Sci. 37, 2887–2905 (2010).

[64] D. Helmer, M. Rocheteau, Atlas de Squelette appendiculaire des Principaux Genres Holocenes de Petits Ruminants du Nord de la Mediterranee et du proche-Orient: (Capra, Ovis, Rupicapra, Capreolus, Gazella) (APDCA, 1994).

[65] J. Boessneck, “Osteological differences between sheep (Ovis aries Linné) and goat (Capra hircus Linné)” in Science in Archaeology: A Comprehensive Survey of Progress and Research, D. R. Brothwell, E. S. Higgs, Eds. (Thames and Hudson, 1969), pp. 331–358.

[66] M. A. Zeder, “Reconciling rates of long bone fusion and tooth eruption and wear in sheep (Ovis) and goat (Capra)” in Recent Advances in Ageing and Sexing Animal Bones, D. Ruscillo, Ed. (Oxbow Books, 2006), pp. 87–118.

[67] I. A. Silver, “The ageing of domestic animals” in Science in Archaeology: A Survey of Progress and Research, D. R. Brothwell, E. S. Higs, Eds. (Thames & Hudson, 1969), pp. 283–302.

[68] A. von den Driesch, A Guide to the Measurement of Animal Bones from Archaeological Sites (Peabody Museum Press, 1976).

[69] S. Boessenkool ., Combining bleach and mild predigestion improves ancient DNA recovery from bones. Mol. Ecol. Resour. 17, 742–751 (2017). 27790833 10.1111/1755-0998.12623

[70] A. W. Briggs ., Removal of deaminated cytosines and detection of in vivo methylation in ancient DNA. Nucleic Acids Res. 38, e87 (2010). 20028723 10.1093/nar/gkp1163 PMC2847228

[71] M. Meyer, M. Kircher, Illumina sequencing library preparation for highly multiplexed target capture and sequencing. Cold Spring Harb. Protoc. 2010, pdb.prot5448 (2010). 20516186 10.1101/pdb.prot5448

[72] D. M. Bickhart ., Single-molecule sequencing and chromatin conformation capture enable de novo reference assembly of the domestic goat genome. Nat. Genet. 49, 643–650 (2017). 28263316 10.1038/ng.3802 PMC5909822

[73] H. Li, R. Durbin, Fast and accurate short read alignment with Burrows-Wheeler transform. Bioinformatics 25, 1754–1760 (2009). 19451168 10.1093/bioinformatics/btp324 PMC2705234

[74] M. Schubert ., Improving ancient DNA read mapping against modern reference genomes. BMC Genomics 13, 178 (2012). 22574660 10.1186/1471-2164-13-178 PMC3468387

[75] T. S. Korneliussen, A. Albrechtsen, R. Nielsen, ANGSD: Analysis of next generation sequencing data. BMC Bioinformatics 15, 356 (2014). 25420514 10.1186/s12859-014-0356-4 PMC4248462

[76] R. C. Edgar, MUSCLE: Multiple sequence alignment with high accuracy and high throughput. Nucleic Acids Res. 32, 1792–1797 (2004). 15034147 10.1093/nar/gkh340 PMC390337

[77] L. Excoffier, H. E. L. Lischer, Arlequin suite ver 3.5: A new series of programs to perform population genetics analyses under Linux and Windows. Mol. Ecol. Resour. 10, 564–567 (2010). 21565059 10.1111/j.1755-0998.2010.02847.x

[78] S. Guindon ., New algorithms and methods to estimate maximum-likelihood phylogenies: Assessing the performance of PhyML 3.0. Syst. Biol. 59, 307–321 (2010). 20525638 10.1093/sysbio/syq010

[79] R. Bouckaert ., BEAST 2.5: An advanced software platform for Bayesian evolutionary analysis. PLoS Comput. Biol. 15, e1006650 (2019). 30958812 10.1371/journal.pcbi.1006650 PMC6472827

