# Supplementary material for: Herded and hunted goat genomes from the dawn of domestication in the Zagros Mountains
Source: Proc Natl Acad Sci U S A. 2021 Jun 7;118(25):e2100901118. doi: 10.1073/pnas.2100901118 (PMC8237664; doi:10.1073/pnas.2100901118)
Supplement: Supplementary File [file pnas.2100901118.sapp.pdf]

PNAS
www.pnas.org

Supplementary Information for:

Herded and hunted goat genomes from the dawn of domestication in the Zagros Mountains.

Kevin G. Daly Valeria Mattiangeli, Andrew J. Hare, Hossein Davoudi, Homa Fathi, Sanaz Beizaee Doost, Sarieh Amiri, Roya Khazaeli, Delphine Decruyenaere, Jebrael Nokandeh, Tobias Richter, Hojjat Darabi, Peder Mortensen, Alexis Pantos,, Lisa Yeomans, Pernille Bangsgaard, Marjan Mashkour, Melinda A. Zeder, Daniel G. Bradley

Email: dalyk1@tcd.ie, zederm@si.edu, dan@palaeome.org

This PDF file includes:

Supplementary text

Figures S1 to S32

Tables S1 to S19

Legends for Dataset S1

SI References

Providence and groupings used for ancient genome samples: The geographic origin, identity of site, and cultural/temporal period associated with each ancient sample are displayed in Table S12. Sample identifier, molecular sex, mitochondrial haplogroup, and autosomal genome coverage (where applicable) are also shown.

Material: Material from the Smith excavations (1) were obtained, photographed and sampled (subsampled from surrounding petrous bone using a dentist drill and dremel saw) at the Ancient DNA facilities at the Smithsonian National Museum of Natural History, Washington DC; this material was exclusively from the petrosal part of the temporal bone. Material from the Iranian/Danish excavations were sampled from University of Copenhagen's Department of Cross-Cultural and Regional Studies, and were composed of a mixture of petrosal and postcranial bone elements. Sample identifiers, provenance information (i.e. Level for Smith excavation, Areas and Test Pits for Iranian/Danish excavations), and bone types are shown in Table S8.

Bone metric analysis: The analysis of the goats from earlier excavations from Ganj Dareh, Asiab, and Late Pleistocene sites was conducted in the late 1990s and early 2000s by Zeder, assisted by Heather Lapham and a large team of students, volunteers, and interns. Analysis of faunal remains from later excavations at Ganj Dareh were analyzed by Lisa Yeomans and Pernille Bangsgaard; and the analysis of Tepe Abdul Hosein faunal remains by Marjan Mashkour, Hossein Davoudi, Homa Fathi, Sanaz Beizaee Doost, Sarieh Amir and Roya Khazaeli. Species identification of caprine were made following criteria in (2–8), and sexing criteria was derived from (9) and (10); and metric followed (11). Bone elements used were humerus, radius, first and second phalanges, metacarpal, metatarsal, tibia, and calcaneus.

Note on log normalized metric profiles, Figure S7

Log normalized metric profiles from a range of modern and prehistoric goat assemblages from the upland Zagros region are displayed in Figure S7. Modern bezoars show strong sexual dimorphism in the marked difference between the size of male and female bones. Modern bezoars are also considerably smaller than goats from Late Pleistocene (g) and Early Holocene sites including Ganj Dareh (f and g). Size reduction in modern wild animals compared to those from Late Pleistocene/Early Holocene contexts has also been observed in wild sheep, boar, gazelle and foxes, and is thought to be a reflection of changing climatic conditions over time (12–14).

While there are more of the larger goats in the Paleolithic and Epi-paleolithic sites (g and f) than at Ganj Dareh (e), the overall size range of goats from these sites is roughly the same. Moreover, the difference between the sizes of goats from these sites is similar to that observed in modern male and female wild goats. These differences are likely a reflection of dimorphic differences between these assemblages. Assemblages from earlier sites are dominated by males as a result of a prime age male hunting strategy, while the assemblage from Ganj Dareh is dominated by those of females as a result of both a management strategy that culls young males and reserves the slaughter of females until they are passed peak reproductive years.

Goats from the sites that date to the end of the aceramic Neolithic and the beginning of the Ceramic Neolithic (a and b) also appear to be dominated by smaller female animals consistent with managed herds. The leftward shift in the overall size range of these assemblages indicates that goats at this time have undergone significant reduction in overall body size. Sarab goats also show changes in horncore morphology consistent with domestication (15). Note, however, that the domestic goats from these later assemblages have the same size range as modern bezoar goats highlighting the difficulty of discriminating between domestication-induced body size reduction and the impact of changing climate since the Late Pleistocene/Early Holocene.

Correlation between phasing in Smith and Danish/Iranian excavations of Ganj Dareh: The original excavations at Ganj Dareh identified five phases Levels A-E. The earliest level (Level E) consisted of a series of pits and was subsequently followed by a phase of mudbrick architecture (Level D) that was subsequently burnt. Three levels of subsequent architecture were described as Levels C to A. The Danish/Iranian excavations re-excavated the original trenches and conducted additional excavations of *in situ* deposits to clarify the stratigraphic sequence in trenches A1, A2 and D. In Area A1 and A2 the earliest phase A2.2 corresponds to Smith Level E, Smith level D was subdivided into four sub-phases beginning with Phase A2.1 then A1.8, A1.7 and A1.6 the latest. Exact correlation between the individual phases Smith defined as Levels A-C and the Danish/Iranian excavations was not possible but the three architectural phases excavated by Smith (Level A-C) were divided into five phases with the earliest A1.5 and the latest A1.1. In Trench D, Phase D.7 corresponds to Smith Level E, Phase D.6 represents a later phase of Level E and Phases D.5-D.1 are subdivisions of the Smiths architectural levels.

Radiocarbon date calibration: Radiocarbon date calibration (2 sigma) was performed using Oxcal 4.4 (Ramsey, 2009) and IntCal20 (Reimer et al., 2020). The resulting calibrated age 95.4% distributions are displayed in Figure S3. Uncalibrated ages, standard deviations, and calibrated age distributions are shown in Table S2.

Sample Preparation: All pre-PCR steps were performed in dedicated aDNA facilities in Trinity College Dublin, Ireland, according to standard protocols (16).

Each caprid bone element was cleaned externally using a dentist drill and drill bit. To decontaminate the bone surface UV light applied on both sites for 15 minutes each. ~130mg of bone was cut from the sample using a dremel saw, and which was reduced to a fine powder using a Mixer Mill (MM 200, Retsch). Air control tubes were included for all subsequent steps.

Sample Extraction and Library Construction: Bone powder samples were then subjected to a 15 minute wash with 1ml of 0.5% sodium hypochlorite solution, modified from a previously reported method (17). Tubes were then spundown, the supernatant removed, and three successive washes with UVed H_2O performed.

Washed samples were subject to an EDTA-proteinase K digest at 37°C as previously described (18) with modifications (19–21). 16.25µl of the purified DNA was treated with Uracil-DNA-glycosylase (UDG) (22) as described previously (21), and the resulting DNA used to construct dsDNA NGS libraries (23), with modifications (20).

Control tubes were added at the extraction and library stages, and were kept for subsequent steps.

Screening and sequencing: Caprid samples were amplified and screened on an Illumina MiSeq platform (50bp, single-end; at TrinSeq, Trinity College Dublin, Ireland) as previously described (21). Species and endogenous DNA content was determined using Fastq Screen (24). Control tubes did not show evidence of substantive DNA presence.

To confirm residual ancient damage, identified goats were aligned using bwa aln (25) to the goat reference genome ARS1 (26), and assessed using mapDamage 2.0 (27). All samples showed evidence of 5' C>T and 3' G>A error (Figure S8), declining with distance from the read end, consistent with an ancient origin.

Caprid samples with >10% endogenous DNA were selected for deeper sequencing, either on a HiSeq 2500 platform (Single-end 100bp; Macrogen Inc., 1002, 254 Beotkkot-ro, Geumcheon-gu, Seoul, 153-781, Republic of Korea) or a NovaSeq 6000 (pair-end 50bp; TrinSeq, Trinity College Dublin, Ireland).

The remaining sample libraries were enriched for mitochondrial reads using an in-solution bait-and-capture method (28, 29), following the protocol reported (21, 30) and using custom RNA baits (MYcroarray, 5692 Plymouth Road, Ann Arbor, MI 48105, USA). Enriched libraries were sequenced using an Illumina MiSeq platform (50bp, Single-end; TrinSeq, Trinity College Dublin, Ireland).

Data processing - nuclear genome: Single-end (SE) reads were trimmed to remove adaptor sequence and filtered to remove reads with <30 bp using cutadapt1.1 (31) (cutadapt -a AGATCGGAAGAGCACACGTCTGAACTCCAGTCAC -O 1 -m 30).

For deeply sequenced samples SE reads were aligned to the RefSeq goat reference ARS1 (26) using bwa aln (25) with relaxed parameters (32) to account for residual damage. Using samtools v.1.4.1 (33), bam files were produced (samtools view) then filtered for minimum mapping quality 30 (samtools view -q 30), sorted, and then filtered for duplicates (samtools rmdup). Read groups were assigned based on sequencing index and PCR reaction.

Previously reported sequencing reads from ancient goat samples (21) and a historic *Capra caucasica* genome (34) were downloaded from ENA and aligned using the same pipeline.

For paired-end (PE) reads, AdapterRemoval v2.3.1 (35) was used to remove adapter sequences, remove reads with <30bp length, and to combine overlapping read pairs (--collapse --minadapteroverlap 1 --adapter1 AGATCGGAAGAGCACACGTCTGAACTCCAGTCAC --adapter2 AGATCGGAAGAGCGTCGTGTAGGGAAAGAGTGT --minlength 30 --trimns --trimqualities). Collapsed reads were treated as SE reads and processed as above. PE reads not collapsed or discarded were aligned using bwa aln with relaxed parameters, and processed as above but with bam files filtered for properly-pairing reads (view -f 2) and samtools rmdup run in default PE mode.

For each sample, individual read group bams were combined into a single bam file and indel realignment performed using GATK (36). Finally bam files were softclipped by setting the first and last 4bp of each read to base quality 0, using a custom python script. Autosomal coverage statistics were calculated with GATK's DepthOffCoverage, and are shown for all ancient goats in Table S12. Sequencing statistics and alignment to the goat nuclear genome reference are shown in Table S10.

Data processing - mitochondrial genome: Trimmed reads were initially aligned to the RefSeq goat mtDNA reference (NC_005044.2) (37), using the same steps as described above, to determine which of the main haplogroups (A, B, C, D, F, G, T) each sample was closest to. Once a haplogroup assignment was performed, reads were realigned to a circularized version of a representative mtDNA sequence of each of the haplogroups, as specified in (21). Consensus fasta files were generated using ANGSD (38): doFasta 2 -doCounts 1 -setMinDepth 3 -minQ 20 -minMapQ 30, and then decircularized (15bp at both ends removed) to produce final mtDNA sequences. Final alignment statistics for mtDNA and capture sequencing reads are displayed in Table S11.

Data processing - modern goat data: Sequencing data for published modern goat genomes and an outgroup sheep (Table S13) were downloaded from ENA and aligned to ARS1 using bwa mem (39). PE read mate coordinates and insert size were added with samtools fixmate, duplicates removed using Picard Tools (40) MarkDuplicates, indel realignment performed using GATK, and reads with mapping quality < 30 removed with samtools. Mitochondrial sequences were generated using the same pipeline as for ancient sequences, but using the alignment and filtering steps for modern data. Final coverage data is presented in Table S13.

Mitochondrial phylogeny: mtDNA sequences generated here were combined with a dataset of published previously ancient (21) and modern (41) sequences, and a Nubian ibex mtDNA (NC_020624.1) added as an outgroup. Sequences were aligned using MUSCLE (42) and ML phylogeny computed using PhyML 3.0 (43), selecting a substitution model using BIC (44), and performing 100 bootstrap replicates. The phylogeny was visualized with Figtree v1.4.2 (45) and is displayed in Figure S28.

Mitochondrial affinity of Neolithic Levantine mtDNA with mtDNA from Epipaleolithic Anatolia: as noted in the text and demonstrated in Figure S28, four mtDNA sequences from the

PPNC Levels of ‘Ain Ghazal show greater affinity with mtDNA from ancient wild genomes from Epipaleolithic site of Direkli Cave in Taurus Mountains, southern Anatolia (Direkli4), as well as the Late Pleistocene Armenian bezoar Hovk1. In comparison, the single F mtDNA sequence from the AN Zagros samples, Ganjdareh35, forms a clade with a F mtDNA from wild bezoar ibex from Iran (41), as well as F sequence from Azerbaijan and Qazvin/Alborz. This supports a role of (matrilineal) gene flow from wild populations diverged from the ancestral population of the AN Zagros goat, possibly closely related to Anatolian or unsampled Levantine bezoar.

AMOVA: Analysis of molecular variance was performed on Neolithic populations using Arlequin v3.5 (46). Samples with >25% missingness were not included in the analysis. The groupings used for Neolithic samples are displayed in Table S12. Maximum missing data per site was set at 0.05. Significance of variance components and Fixation Indices were computed using 1000 permutations. Arlequin estimates of F_{ST} and average number of pairwise differences are shown in Table S16 and S18 respectively.

BEAST: A Beast2 (47) analysis was performed on modern mtDNA sequences, ancient mtDNA with associated radiocarbon dates, and the mtDNA reported here. Nubian ibex was used as the outgroup. Partitions (1st+2nd codon position, 3rd codon position, D loop, remainder of molecule) were used for clock and site models, and are described in (21). A relaxed lognormal clock was used for the clock model. Normally-distributed tip uncertainty of ancient samples was set using either calibrated radiocarbon ages for the directly dated samples, setting mean to the midpoint of the 2-sigma calibrated range and sigma as one fourth of that range. For Ganj Dareh and Tepe Abdul Hosein samples without direct dates, normal distributions were used with the 2.5% quantiles matching the maximum and minimum 2-sigma age estimates of each site based on the dates reported here (Ganj Dareh: mean 9716 BP, sigma 87; Tepe Abdul Hosein: mean 9887 BP, sigma 169). Age prior for Hovk1 was set as an uniform distribution [40000, 200000], starting at 120000 yBP. Site models were estimated while accounting for uncertainty (48). Two independent runs of 500 million chains were performed using 50% burnin, with an initial run performed to calibrate operators. Run mixing was assessed using Tracer (49), with all parameters showing good mixing (ESS > 200) except the height of the Hovk1 sample (ESS=128); therefore the height of Hovk1 must be considered with some caution. Trees were combined by maximum clade credibility and median heights, with 50% burn-in and subsampled to 10000 trees, with the resulting tree presented in Figure S29.

ANGSD analyses: For all analyses using ANGSD, major and minor alleles were set using a sheep as ancestral genome (-doMajorMinor 5), which a consensus sheep genome generated using ANGSD (-doCounts 1 -minQ 20 -minMapQ 20 -skipTriallelic 1) using reads generated for the sheep genome project (SRS589386, 50). ANGSD analyses were restricted to the autosomes. When applicable triallelic sites (-skipTriallelic 1) and transition variants (-rmTrans 1 / -noTran 1) were ignored, and a SNP filter was applied (-SNP_pval 1e-6).

Genotype Likelihoods and PCA/ancestry estimation: To investigate how Aceramic Neolithic (AN) goat from the Zagros relate to modern and other ancient goat, genotype likelihoods were

computed with ANGSD (-GL 1 -doGlf 2) (38). Modern domestic goats were randomly subsampled to ten per geographic region (Table S13). Ancient goats were filtered for those with at least 0.01X coverage.

PCAngsd (51) was used to estimate a covariance matrix and ancestry profiles using genotype likelihoods. A minor allele frequency filter of 0.05 (-minMaf 0.05) was applied; otherwise default settings were used. PCAngsd ancestry estimation was run twice, once with i) all moderns and ancients >0.01X (to jointly assess all available goat genomes), and ii) modern bezoar and ancients >0.1X (to assess ancestry estimation of the AN Zagros groups without possible effects from low coverage individuals or recent genetic drift in moderns). K=5 and K=6 were selected as the number of ancestral populations respectively, based on estimated individual allele frequencies determining the optimal number of principal components which describe the data. Principal Components 1 and 2 for i) are displayed in Figure 3.

For ancestry estimation in i), displayed in Figure S9A, ancestry components were maximized in modern Azerbaijan bezoar (green), Alborz bezoar (purple), Zagros bezoar (red), east Asian domestic goat (blue), European goat (orange), and African goat (yellow). *Zagros Main* genomes from both Tepe Abdul Hosein and Ganj Dareh show similar ancestry profiles - primarily "Alborz bezoar" (purple) and "east Asian domestic" (blue) - alike to the profiles of the "Markazi bezoar" genomes from west (Markazi) and southwest (Fars) Iran. The *Zagros Outlier* genomes (Abdul4 and Ganjdareh35) differ from other AN Zagros genomes by their lower proportion of "east Asian domestic" and "African domestic" ancestries, and a greater amount of "Alborz bezoar" (purple) and "Azerbaijan bezoar" (green) ancestries. In the case of ii) (Figure S9B), ancestral components were maximized in Alborz bezoar (blue), Azerbaijan bezoar (orange), Zagros bezoar (purple), Neolithic east Iranian (Tappeh Sang-e Chakhmaq / Semnan) and Turkmenistan (Monjukli) goat (red), and Neolithic Serbian goat (Blagotin; green). *Zagros Outlier* (Abdul Hosein and Ganj Dareh) samples differed from *Zagros Main* by having less "Eastern Neolithic" (red) ancestry and more "Alborz Bezoar" (blue) and "Azerbaijan Bezoar" (orange) ancestries. Both datasets therefore support *Zagros Outlier* genomes having distinct ancestral makeup from *Zagros Main* genome, not dissimilar to modern wild goats from west/southwest Iran (Markazi and Fars) and different from modern Zagros goats.

Markazi/Fars bezoar ibex appear admixed: We note also that wild goats from southwest Iran ("Markazi Bezoar") show possible evidence of admixture with Asian domestics, reflected in their ancestry profiles being a mixture of wild (red and purple) and asian domestic (blue and yellow) components (Figure S9A). While similar to *Zagros Main* profiles, a previously published ADMIXTURE analysis using a wider SNP and sample set with modern genomes also identifies these samples as consistently carrying Southwest or East Asian-related domestic ancestry in each value of K (34). In addition, chromopainter analyses from the same paper show these three genomes (IRCA17, IRCA23, IRCA24 in that paper) as having reduced haplotype sharing with bezoar ibex from Alborz and Iranian Azerbaijan, and high haplotype sharing with Asian domestics. However, the latter of these may be confounded by wild admixture into the ancestry of west Eurasian goats, and assignment to multiple ancestry clusters in ADMIXTURE can be

due to several scenarios (including admixture and sampling bias, see (52)). As such, care should be taken in interpreting the PCA (Figure 3), IBS-nj phylogeny (Figure 4) or Treemix (Figure S16) position of the Markazi bezoar genomes. Here, we have erred on interpreting the position of the *Zagros Outlier* genomes as more reliable due to chronological placement prior to the suggested demographic events which could alter the genetic affinity of present-day Markazi bezoar ibex.

Identity-by-state (IBS) estimation: ANGSD was used to generate an IBS matrix, using random sampling of reads. Two datasets and approaches were used: i) modern bezoar and downsampled domestics and ancient genomes with >1X, with and without 50 bootstraps (5 Mb), and ii) >0.01X ancient genomes with no modern domestic genomes and no bootstrapping due to computational limitations. In both cases a minimum frequency filter was applied (-minFreq 0.05). IBS matrices were converted to nj trees using the r package ape (53). For i), bootstrap values were applied to the non-bootstrapped phylogeny using raxml (54): raxmlHPC -f b -t nonboot.newick -z boot_combined.newick -m GTRCAT -o Sheep -n out. Clades were collapsed using Figtree (45), and displayed in Figure 4 in its collapsed form. The uncollapsed consensus tree for i) is displayed in Figure S14, and the nj tree generated for the ii) data set is displayed in Figure S15.

Modern Genotype Calling: To generate genotype calls for modern genomes, samtools mpileup (33) was employed on a data set including all modern genomes >8X coverage, and the outgroup sheep genome. The following parameters used: " -C 50 -q 30 -Q 20 -s -O -u -l <bed.gz>", where bed.gz referred to a bed file describing autosome coordinates minus repeat and genic regions plus 50 kbp buffers. The resulting vcf was piped to bcftools v1.2 (http://samtools.github.io/bcftools/) and filtered for variants ("-v -mO z -f GQ,GP") and indels (bcftools filter --SnpGap 3 --remove-indels --recode --recode-INFO-all). Sites were limited to biallelic variants using vcftools (55). Using a custom script and for each individual, sites were set to missing if read depth was below 5 or above twice the sample mean coverage, if strand bias (SP) was below 13, or if heterozygous sites with < 0.3 or > 0.7 of reads supporting one of the two alleles. Individuals with missingness >0.10 were then filtered from the dataset, and the remaining vcf filtered for sites with no missingness (vcftools --max-missing 1), and transversion variants. Plink (56) ped and map files were generated with vcftools and filtered for MAF of ≥0.02 (--maf 0.02 --max-maf 0.98) and filtered for LD (--indep-pairwise 50 5 0.5).

Ancient Genotype Calling: Ancient and low coverage samples were then called at the sites described above using samtools mpileup as described above, filtering also indels and biallelic sites. For each individual, variant sites were pseudohaploidized by random base selection and setting that site as homozygous for the selected base. The ancient and low coverage vcf were combined with modern variants and filtered a final time for biallelic sites, producing a final set of 56,682 biallelic transversion variants pruned for LD.

Treemix: To reconstruct a phylogeny of ancient and modern goat modelling possible gene flow events, Treemix (57) was used on domestic and wild goat genomes with >2X coverage.

Samples were grouped based on the labels in Table S12. Sites were filtered such that each site was called in at least one individual in each group, producing a set of 32,944 biallelic transversion sites with no group-level missingness. Treemix was then run using blocks of 500 SNPs for values of *m* from 0 to 8, performing 50 bootstraps, rooting on Sheep and with sample size correction disabled (-k 500 -root Sheep --noss --boot --global). Optimal *m* was selected using optM's linear method (https://cran.r-project.org/web/packages/OptM/index.html), based on the change-point of log likelihoods across values of *m*, which favour a single migration edge model (Figure S16C). A consensus tree was built using phylip (58) consensus majority rule; the resulting tree was visualized with Figtree (45). Bootstrap values were added to a representative bootstrap tree with Inkscape (59); the tree is displayed in Figure S16A, along with residual values of the tree/migration model (Figure S16B). Note that the node relating wild goat to domestic goat, showing a bootstrap value of 0.46, differs among bootstrap iterations in the relative ordering of Epipaleolithic Taurus and the {Alborz Bezoar, Azerbaijan Bezoar, Paleolithic Armenia} wild clade, with the overall structure of the tree maintained.

***D* statistics:** *D* statistics were computed using ANGSD, using random read sampling (-doAbbababa 1). Sheep was used to define the ancestral allele. Biallelic sites were limited to transversions (-rmTrans 1), and analysis restricted to the autosomes. *D* statistic test results are displayed in Figure S11-13, and S19, and are reported in full in Dataset S1.

***f* statistics:** for *f*-statistic based analyses, ADMIXTOOLS (60) was used to convert plink format files to the eigenstrat format. For outgroup f_3 analyses, groupings were used (Tables S12, S13) and a pooled group of modern wild goat as the outgroup population. To construct a heatmap of the resulting values, the R function heatmap.2 was used (61), first removing all Levantine groups and the Ceramic/Pottery Neolithic Zagros groups due to low SNP number. The heatmap is displayed in Figure S10.

qpAdm: To explore models of the ancestral make-up of the AN *Zagros Outlier* cluster, qpAdm was employed in an iterative fashion (https://github.com/pontussk/qpAdm_wrapper) to explore all one-source and two-source models. We used a range of modern wild (Alborz Bezoar, Markazi Bezoar, Zagros Bezoar, Azerbaijan Bezoar) and ancient (*Zagros Main*, Epipaleolithic Taurus, Upper Pleistocene Armenia) populations as potential Sources, and including Sheep as an outgroup. Results are displayed in Table S14; models are accepted if i) p value <0.05, ii) non-negative admixture proportions, iii) admixture proportions +/- SE are >0. Among single-source models, only Zagros Bezoar was not rejected (p=0.58), consistent with it forming a clade with the *Zagros Outlier* group. Among two-source mixing models, four of the seven accepted models include *Zagros Main* and an ancient or modern wild population, while four of the seven include the modern wild Markazi Bezoar group. Although these data support the *Zagros Outlier* cluster being closely related to modern wild goats from the same region, we highlight the limitations of the reference data: few ancient wild populations of low sample size, and several modern wild populations which may be poor proxies for past populations. As such, we do not rule out more complex ancestries for the *Zagros Outlier* cluster, such as mixing between *Zagros Main* and a distinct wild population.

We also note that model acceptance/rejection can be affected by differing error rates among Sources and Targets, and by the use of single-genome populations (62). However, the SNP set used here is transversion-only and should not be affected by ancient-driven transition errors; to confirm such errors do not drive results we repeated the analysis using modern source populations only (Table S14). Two single-source models are accepted: Markazi Bezoar (p=0.9) and the outgroup (p=0.09). Two two-source models were also accepted: Alborz Bezoar + Markazi Bezoar (p=0.42) and Azerbaijan Bezoar + Markazi Bezoar (p=0.64). Although the significance of the outgroup result illustrates the limitations of the data set available, the remainder indicates that the Neolithic *Zagros Outlier* group may not be well represented by modern Zagros wild goats.

qpGraph: To explicitly model the ancestries of the two clusters of AN Zagros goat, *Zagros Main* and *Zagros Outlier*, using other ancient populations, we constructed admixture graphs and fit the expected *f* statistic values to those observed using qpGraph (60). A historic Tur (*Capra caucasica*) genome was included due to possible admixture into the domestic goat lineage (34). The allSNPs parameter was set to YES, otherwise all parameters were default. |Z| of ≥ 3 was taken to be outliers.

We initially constructed a base graph of Tur, {*Zagros Outlier*, {Pleistocene Armenia, Epipaleolithic Taurus}}, based on previous phylogenies, but this model was rejected (|Z|= 3.6), with f_4 outliers indicating affinity between Tur and Epipaleolithic Taurus wild goat. Modelling Epipaleolithic Taurus wilds as a mixture of Tur and Paleolithic Armenian-like was also rejected (|Z|= 3.0), but modelling both Paleolithic Armenia and Epipaleolithic Taurus as being admixed with Tur produced no outliers, and is in line with previous results (34). To this graph we iteratively placed *Zagros Main* Ganj Dareh as a sister group to each population; all produced outliers (|Z| = 10.3-60.6) except when placed as a sister branch to *Zagros Outlier* (|Z| = 1.9). We repeated this for *Zagros Main* Tepe Abdul Hosein, which was best modeled as a sister group to Ganj Dareh (|Z| = 2.3; alternative positions |Z| = 6.3-53.6) with *Zagros Outlier* being an outgroup to the *Zagros Main* clade. We repeated this process for the Neolithic East Iran/Turkmenistan group and did not fit it as a clade with any one group (|Z| = 3.0 - 62.3), but as a sister the *Zagros Main* clade and with *Zagros Outlier* as the outgroup (|Z|= 1.9). Finally, we iterated on positions to place Neolithic Serbian genomes and rejected any simple clade with other groups (|Z| = 10.5-59.1), with the lowest f_4 outlier found in the model with Neolithic Serbians as a clade with Epipaleolithic Taurus. As f_2 outliers indicated unmodelled affinity with Neolithic East Iran/Turkmenistan (|Z| = -1.3), we modelled Neolithic Serbia as a mixture of the two groups, which was rejected (four outliers, largest |Z| = 4.4), as was using an admixing clade basal to Neolithic Iranian groups (five outliers, largest |Z| = 3.6). Both models indicated unmodelled ancestry shared between Neolithic Serbia and Tur (f_2 = -0.35 and -0.43). When accounting for this in both models, the Neolithic East Iran/Turkmenistan-source model has a single outlier (|Z| = 3.3), while the basal Iranian-source model has no outliers (|Z| = 2.7). This model is presented in a modified form (drift edges removed) in Figure 5, and in an unmodified form in Figure S17, with intermediate population labels removed.

As a complementary approach, we explored admixture graph space in an automated fashion using qpbrute (63, 64). We searched graph space using the following populations: Epipaleolithic Taurus (South Anatolia/Turkey), Neolithic East Iran/Turkmenistan, *Zagros Main* Ganj Dareh, *Zagros Main* Tepe Abdul Hosein, and *Zagros Outlier*, resulting in a single graph which fit the data, recapitulating IBS-tree results. To this scaffold graph we added Neolithic Serbia, using the --qpgraph option of qpbrute to limit the search space to those starting from the previous graph. 7 out of 45 unique possible graph models fit without outliers, and all recapitulated the general phylogeny of Neolithic Serbia being modeled as a mixture of Iranian and Epipaleolithic Taurus wilds. Comparing graphs with the Bayes Factor K, a single model showed pairwise K ranging from 5.9-23.4 (Figure S18A), which models Neolithic Serbia as a mixture of populations related to Neolithic East Iranians to Epipaleolithic Taurus, with *Zagros Outlier* an outgroup to all Neolithic Iranian groups. Again using the --qpgraph option to add a population to the previously fitted graph, we added the *Capra caucasica* sample Tur1, found 7 fitting models by graph space searching, and compared pairwise K values. The best fitting graph (K=2.7-21.8) models Tur as being a mixture of Epipaleolithic Taurus wilds and a basal source (Figure S18B). We note that this graph is the product of successive limiting of the graph space, due to computational limitations. In comparison, the graph presented in Figure 5 is affected by greater user degrees-of-freedom, but better fits with predomestication admixture previously reported (34). A search for graphs fitting Epipaleolithic Taurus, Late Pleistocene Armenia, *Zagros Outlier*, and Tur, finds that a model of Epipaleolithic Taurus and Late Pleistocene Armenia carry ~9% of ancestry from a Tur-related population (Figure S18C) fits the data best (K=7.4-11.7), rather than Tur1 carrying *Capra aegagrus* ancestry. Additional ancient genomes, particularly ancient *Capra caucasica*, may help identify the most likely direction of admixture relating modern *Capra caucasica* to domestic and wild goats.

qpWave: To test the observation that Neolithic Iranian goat populations from the Zagros (*Zagros Main*, ~8,000-7,700 cal BC) and east Iran/Turkmenistan (~7,000 and ~6,000 cal BC) are differentiated by drift alone, we tested the number of “waves” of ancestry from source populations are required to explain the ancestry of both groups. We used four sets of source populations: Wilds (Azerbaijan, Alborz, Zagros, Markazi), Domestics (Iranian, Chinese, French, Moroccan), Wilds+Domestics, and Wilds+Ancient Wilds (addition of Pleistocene Armenian and Epipaleolithic Taurus to the “Wilds” group). The allsnp parameter was set to “YES”. Results are displayed in Table S15. When sources included only wild groups, a single pulse model was accepted (p=0.18 and 0.27 respectively), supporting drift rather than differential ancestry to wild populations to drive differentiation within Neolithic Iranian goats. The inclusion of domestic groups allowed Neolithic Iranian goats to be differentiated as single pulse models were rejected (p= 1.76×10^{-34} and 1.73×10^{-35}), suggesting that it is their relationship to modern domestic goats which allow the groups to be distinguished using the current data.

Molecular sex assignment: Molecular sex was assigned based on the ratio of the number of reads aligned to the X chromosome contigs (NW_017189516.1 and NW_017189517.1) and

their length, compared to that ratio for each autosome, and is explained in greater detail in (65) and (21).

Detecting long ROH: To detect long regions of homozygosity, we first downsampled modern and ancient genomes to 2X mean coverage in order to control for possible coverage effects (66) and noting the likely false positives and false negatives associated with calling heterozygous sites at this level of genomic coverage. Genomes were then called as above (using samtools mpileup v1.4.1 and bcftools 1.5), but calling invariant sites also (bcftools call -mO z), filtering for indels, and removing any called heterozygous sites which did not have at least one read of each allele. For each genome, we used custom scripts to determine the total number of called sites and transversion heterozygous sites in non-overlapping 500kb windows, and calculated the rate of transversion heterozygosity in each window (as the number of sites called as heterozygous in the window divided by the total number of sites called).

To determine a cutoff for transversion heterozygosity possibly indicative of a long region of autozygosity, we followed a similar approach to (67) and used X chromosome transversion heterozygosity in males. As the male X chromosome should be hemizygous, any site called as a (transversion) heterozygote should represent sequencing or alignment error. We repeated the above pipeline for modern male genomes downsampled to 2X, limiting calling to the non-Pseudo Autosomal Regions of the goat X chromosome contigs (all of NW_017189517.1 and NW_017189516.1:1-59449092). A transversion-heterozygous rate was then computed for each individual, which showed a mean of 3×10^{-5} and a standard deviation of 1.33×10^{-5} transversion heterozygotes per bp respectively. To account for variability in error among individuals, the average rate was added to twice the standard deviation to reach a final cutoff rate of 5.66×10^{-5}.

For each individual, 500kbp windows were filtered to remove those with less than 50kbp called sites. Windows were then assessed in blocks of ten (5Mb), sliding one window at a time. If at least nine of the ten windows showed sub-cutoff transversion heterozygosity rates (i.e. below 5.66×10^{-5} transversion heterozygotes per bp), and the (if present) above-cutoff window was not the first or last of the group (so not to erroneously add windows at the ends of a true ROH), each window was assigned as likely long ROH. The block was then moved forward one window and the block of ten windows reassessed; if the following window was more than 10Mb downstream of the previous window (as intervening windows may have been filtered for insufficient coverage), the previous windows were discarded and a new block of ten windows assessed. The distribution of filtered windows along the autosomes and their long-ROH state for each AN Zagros sample with >2X coverage are displayed in Figures S22-25,27, plotted using ggplot (68).

For each sample with coverage >2X, the proportion of windows in long ROH (F_{ROH5Mb}) was calculated as the numbers of assigned ROH windows divided by the total number of windows after filtering for sufficiently called sites. F_{ROH5Mb} values are presented in Figure 6C. Additionally, plotting the number of detected long ROH vs F_{ROH5Mb} indicates that Ganjdareh18 and particularly Ganjdareh22 have an excess of detected long ROH compared to their F_{ROH5Mb}, implying a

history of recent kin breeding (69). However the potential ROH detected here are ascertained on a length of at least 5Mb, and a majority of ROH detected in an individual is often sub 1Mb in length; an ROH profile more representative of the range of ROH length would be required to better infer the likely demographic and breeding histories of goat populations.

We note that HMM-based methods have been demonstrated to be effective in detecting long ROH (megabase-scale) in medium (>4-8X) coverage genomes (66, 70), and that the detection of longer ROH is challenged by the presence of error. By controlling for coverage effects and focusing on multi megabase-scale ROH using transversion we aimed to establish an estimate of kin-breeding prevalence in goat populations through time - likely under-estimated in the AN Zagros goat due to sequencing error (Figure S20) - which can be subsequently explored with methods exploiting known LD patterns (71) or higher coverage data (66, 70).

Y chromosome: To determine variant sites on the goat Y chromosome, modern male samples were called using samtools mpileup as above, but restricting the analysis to Y chromosome contigs (NW_017189563.1, NW_017189885.1, NW_017189685.1). After indel filtering and setting heterozygous sites to missing ("./."), sites were restricted to biallelic variants called in at least 90% of individuals, individuals removed if they had >10% missing genotypes, and a MAF 0.05 filter applied, producing 1,057 variant sites (525 transversions). These sites were then called in ancient male goats, with individuals with less than 10% called sites removed. A combined vcf file was filtered for transersion biallelic variants and converted to the fasta format with a custom script, which was subject to multiple sequence alignment of the concatenated contigs using MUSCLE (42) and a Maximum Likelihood phylogeny was constructed using phyML v3 (43) with 100 bootstrap replicates and selecting a substitution model based on BIC (44). To assign the observed clades to the major Y chromosome haplogroups in goat, the variant sites used to discriminate between the haplogroups (72) were assessed in modern genomes using samtools mpileup, and haplogroups were manually assigned to the phylogeny based on the observed variants (Figure S30). Additionally, ancient male goat with >90% but <99% missing data were included in individual multiple sequence alignments and ML phylogenies with modern samples, using both transition and transversion variants to increase the number of available sites for lower coverage samples (Figure S31); for those samples in both datasets, haplogroups and phylogenetics placement were consistent. Assigned haplogroups and the number of called sites per sample is displayed in Table S17. Assuming a binomial distribution of Y1AA/non-Y1AA haplogroups in AN Zagros goat, the minimum frequency of Y1AA is calculated to be at least ~0.65 at a 5% probability cutoff (binomial probability of 7 success in 7 trials, where "success" is sampling a Y1AA haplogroup and is assumed to be proportional to the population frequency); lower frequencies result in a binomial probability lower than 5%.

Error estimation: Error rates were calculated for ancient genomes with >0.01X coverage using ANGSD. An "error-free" genome was created using ANGSD (-doFasta 1 -doCounts 1 -setMinDepth 13 -setMaxDepth 76 -C50 -minQ 20 -minMapQ 30) based on a high coverage Old

Irish Goat sample. Overall error was then estimated using ANGSD -doAncError and the accompanying R script. Error rates are present in Figure S20.

Contamination estimation: Contamination estimation was performed by creating apileup file (36) at 64 mtDNA sites which discriminate among the main domestic goat mtDNA lineages (41). When more than 10 variant sites were available for a given haplogroup, sites were down sampled to a total of 10. Contamination was expressed as the percentage of bases at that site showing the minor allele, after filtering for base quality of 30. Contamination percentages were also calculated for heteroplasmic sites only i.e. those showing more than one base, to obtain the most conservative contamination estimates. Finally, both calculations were repeated filtering for transversion variant sites to account for the effect of ancient damage. mtDNA contamination estimates are presented in Table S9.

Heterozygosity estimation: To estimate the genome-wide heterozygosity levels of ancient goats, allele frequency likelihoods were estimated for genomes downsampled to 2X using ANGSD, restricting to transversion sites (-dosaf 1 -gl 1 -noTrans 1). Heterozygosity was then estimated using realSFS and the proportion of likely heterozygous sites calculated.

To account for varying error rates among ancient genomes (Figure S20) affecting heterozygosity estimates, error was recalculated on transversion variants only by removing transition variants from ANGSD -doAncError output files during error summation. As ANGSD -doAncError calculates error as an excess of derived alleles relative to the “perfect” genome, negative error rates can occur due to a sample genome having fewer derived alleles than the “perfect” genome. To account for this, a correction factor was applied such that the ancient sample with the lowest error had an error rate of 0. The corrected transversion error was then subtracted from transversion heterozygosity to obtain an error-corrected heterozygosity estimate for each sample. Corrected heterozygosity values are presented in Figure 6A.

Pairwise kinship coefficients: lcMLkin (73) was used to estimate kinship coefficients. Transversion variant sites called in modern goat and passing MAF and LD filters (see “Modern Genotype Calling”) were used as variant sites. For each sample, the highest 5 pairwise values are displayed in Figure S21, after filtering for pairwise comparisons with >1000 SNPs. The top kinship values for Ganj Dareh are with other Ganj Dareh and Tepe Abdul Hosein samples, and vice versa. The Aceramic Zagros samples have low maximum values (~0.1) compared to later Neolithic populations, while later CN goats from Serbia and east Iran have higher pairwise kinship values (means of 0.234 and 0.242, Wilcoxon rank-sum p= 9.725×10^{-5} and 1.37×10^{-15}).

Outlier scan: To detect putatively swept regions due to positive selection during the onset of management, we employed a θ outlier approach similar to the method employed in (21). Using ANGSD, we calculated $\theta_{Waterson}$ in AN *Zagros Ma*in goat with >2X coverage, and also in modern wild goat filtered for individuals with less than 8X coverage, and also those showing high genetic similarity with domestic goat in PCA space (Markazi and Zagros bezoar) due to possibility of domestic-to-wild gene flow. For moderns, the following parameters were used to estimate the

SFS: -C 50 -setMaxDepthInd 21 -minInd 1 -minIndDepth 4 -rmTrans 1. For AN Zagros, parameters used were: -rmTrans 1 -setMaxDepthInd 10 -minIndDepth 2 -minInd 2. Log ($\theta_{Wild}/\theta_{ZagrosMain}$) and F_{ST} between the two groups were computed in 50 kbp windows and 10 kbp steps. Windows were filtered to remove those with less than 5000 called sites in both groups, and were considered to be outliers if they were in the remaining bottom 5th percentile of log($\theta_{Wild}/\theta_{ZagrosMain}$) and <0, and in the top 1 % of Fst.

Additionally, to detect differentiated regions specific to AN Zagros goats, the Population Branch Statistic (PBS) was calculated among the three groups of >2X *Zagros Main*, modern bezoar, and modern domestic goat. For modern domestic goat, the following parameters were used in SFS estimation: -C 50 -setMaxDepthInd 42 -minInd 44 -minIndDepth 4 -rmTrans 1. Windows were filtered for those with at least 5000 called sites in each group, and the top 0.1% of PBS scores extracted. Protein coding genes (as defined by RefSeq annotations) found to overlap either the top 0.1% PBS windows, or overlap/are nearest neighbours to F_{ST}/θ outlier windows are displayed in Table S19.

Gene Ontology enrichment was performed using g:Profiler (74), and was applied to outlier windows detected using F_{ST}/θ and also windows in top 0.1% of PBS scores.

Previously selected genes (*MUC6*/*STIM1-RRM1*): To assess the genotypes of AN Zagros goat at genes identified as being under selection in goat during their domestication history (34), genotype likelihoods were computed at sites identified as close to fixed for the derived allele in modern goat as per Zheng et al (2020). For each AN Zagros genome, ANGSD was used to compute genotype log likelihoods at these sites (-GL 1 -doGlf 4), and the highest likelihood taken as the most likely genotype. Genotypes are displayed in Figure S32.

Bam files were also directly visualized with IGV (75) to assess low-confidence genotypes. For *MUC6*, Ganjdareh22 has a single heterozygous site from a one derived T read, and one ancestral C; as this is possibly due to transition-error, the sample is likely homozygous-ancestral for the locus. For *STIM1-RRM1*: Abdul1 has one derived homozygous call due to one read with the derived C variant; as this sample is poorly covered we cannot conclude whether the sample is heterozygous or homozygous ancestral with an error-containing read. Ganjdareh26 has a single site called as heterozygous due to one read containing the derived A and one with the ancestral G; given the ancestral genotypes at other called sites, the sample is likely homozygous ancestral. Abdul4 appears to have a heterozygous genotype, based on higher coverage of that sample and multiple occurrences of derived variants across the locus.

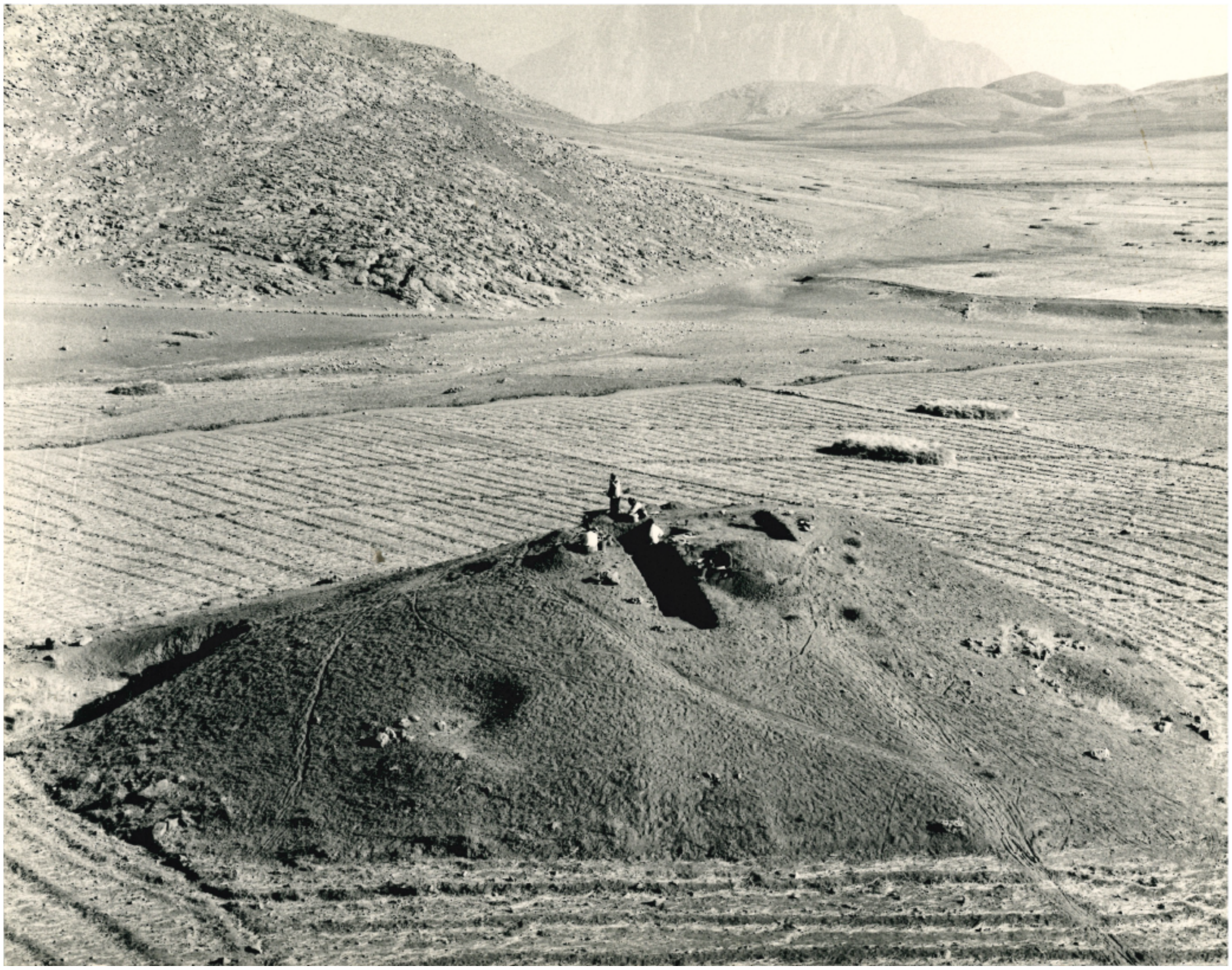

Fig. S1. View of Ganj Dareh during the first (1967) full season of excavation. Photograph GD-UdeM-Site-02-0001, Ganj Dareh Archive, Département d'anthropologie, Université de Montréal, courtesy of Julien Riel-Salvatore

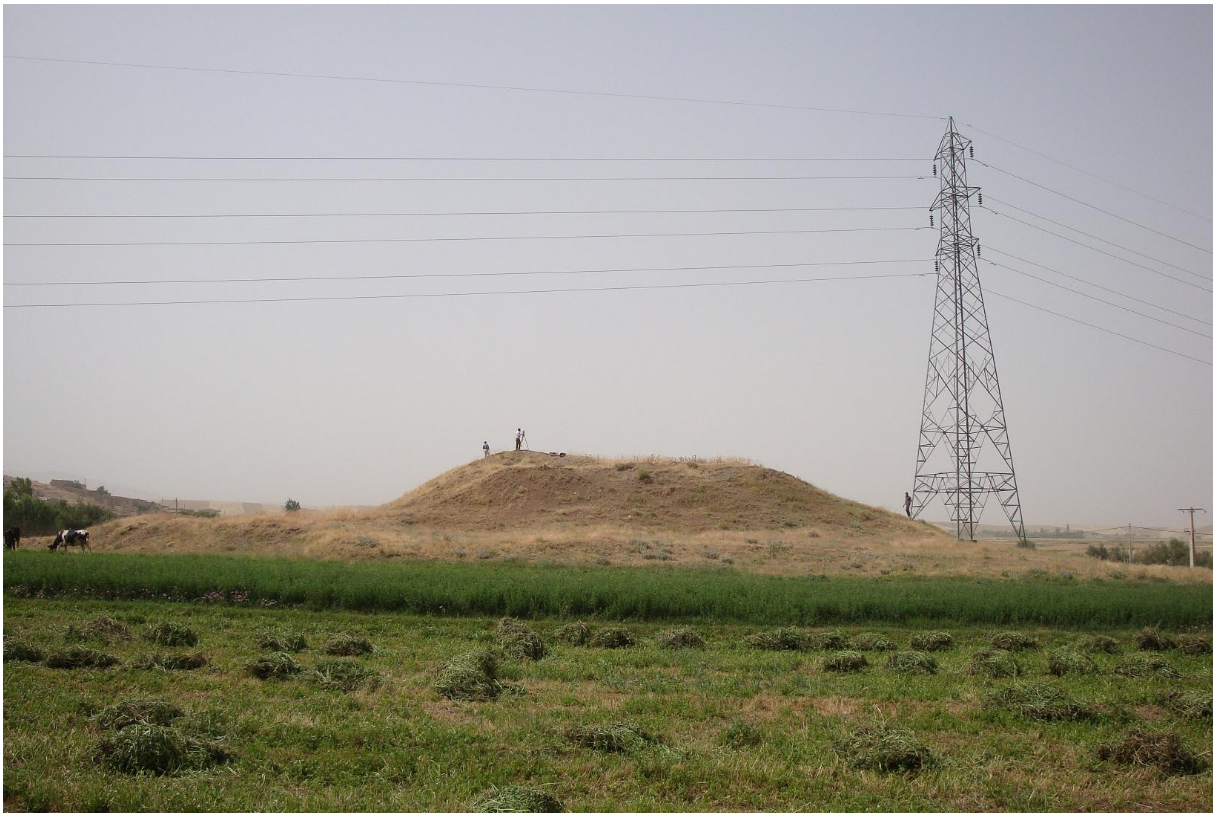

Fig. S2. General view of Tepe Abdul Hosein, looking to the south. Photo by Mousa Sabzi, 2016.

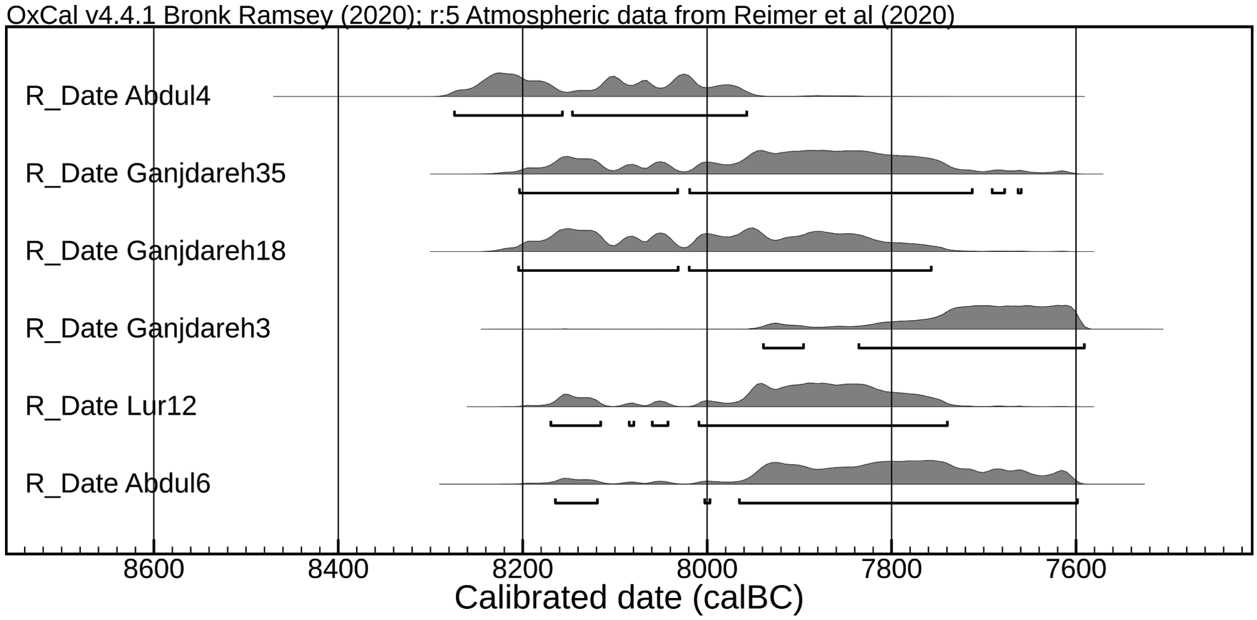

Fig. S3. C_{14} calibration curves for dates reported here, and a previously reported sample (Lur12) from Tepe Abdul Hosein (21). Calibration was performed using Oxcal 4.4 (76) and IntCal20 (77).

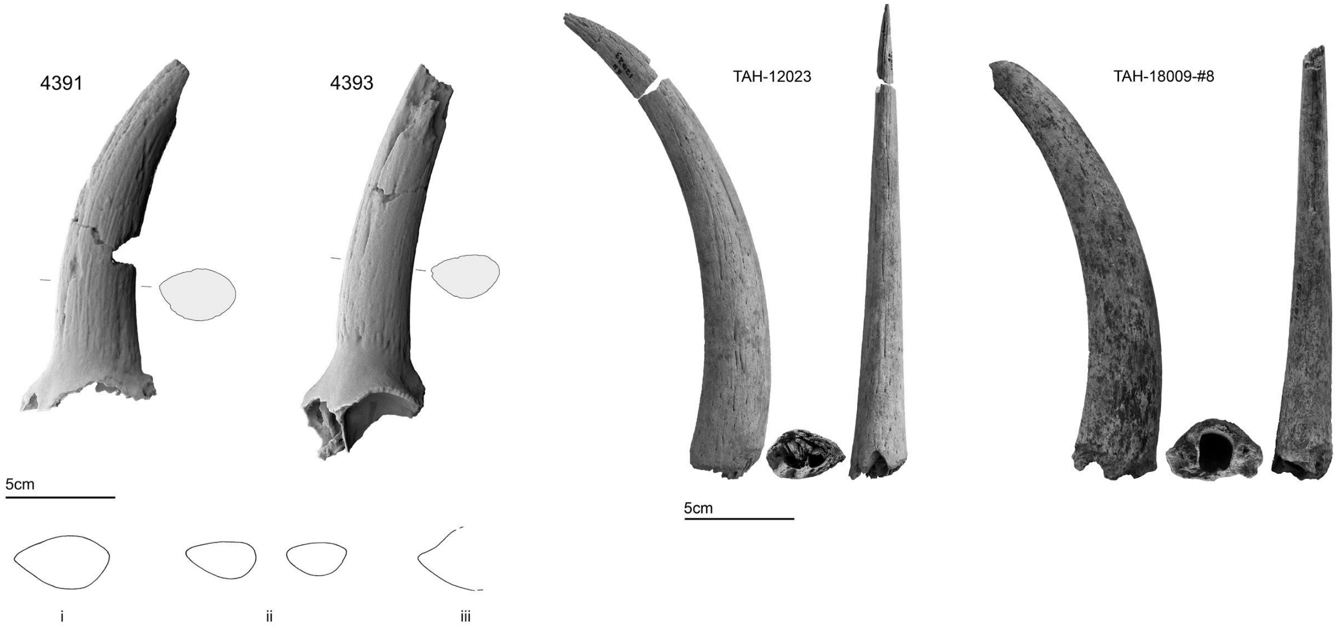

Fig. S4. Comparison of goat horncores from Ganj Dareh, Tepe Guran, and Tepe Abdul Hosein. Left side, top row - two morphologically wild horncores from Ganj Dareh with identifiers (from contexts (5063) and (5053) respectively, Trench D, phase D.3 from Danish/Iranian excavation, corresponding to Smith's Levels A-C). Left side, bottom row – i) morphologically wild horncore from Tepe Guran, ii) morphologically domesticated horncore from Tepe Guran showing size diminution and medial flattening and iii) morphologically domesticated horncore from Tepe Guran with twisting. All drawn as if from the left side. Tepe Guran horncore transects are derived from Figure 97 of (78). Right side: two right horncores with wild morphology from EPPN (12023) and LPPN (18009-#8) phases at Tepe Abdul Hosein 1978 (courtesy of National Museum of Iran).

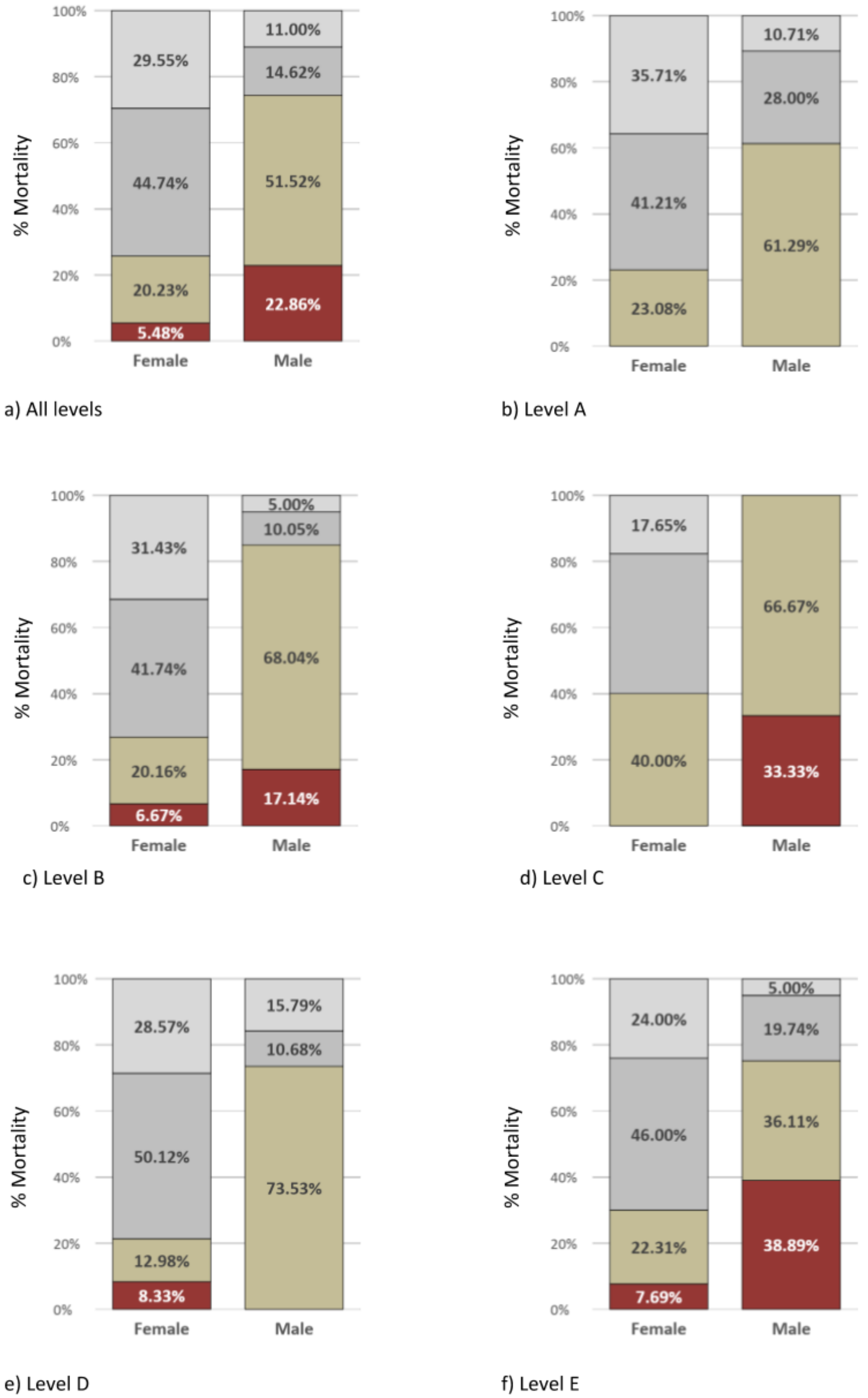

Fig. S5. Mortality data for females and male goats at Ganj Dareh showing percentage of animals slaughtered in each age grouping. a) Whole sample, b) Level A, c) Level B, d) Level C, e) Level D, f) Level E. Red = 0-18 months, Tan=18-30 months, Dark Grey=30-46 months, Light Grey=> 46 months.

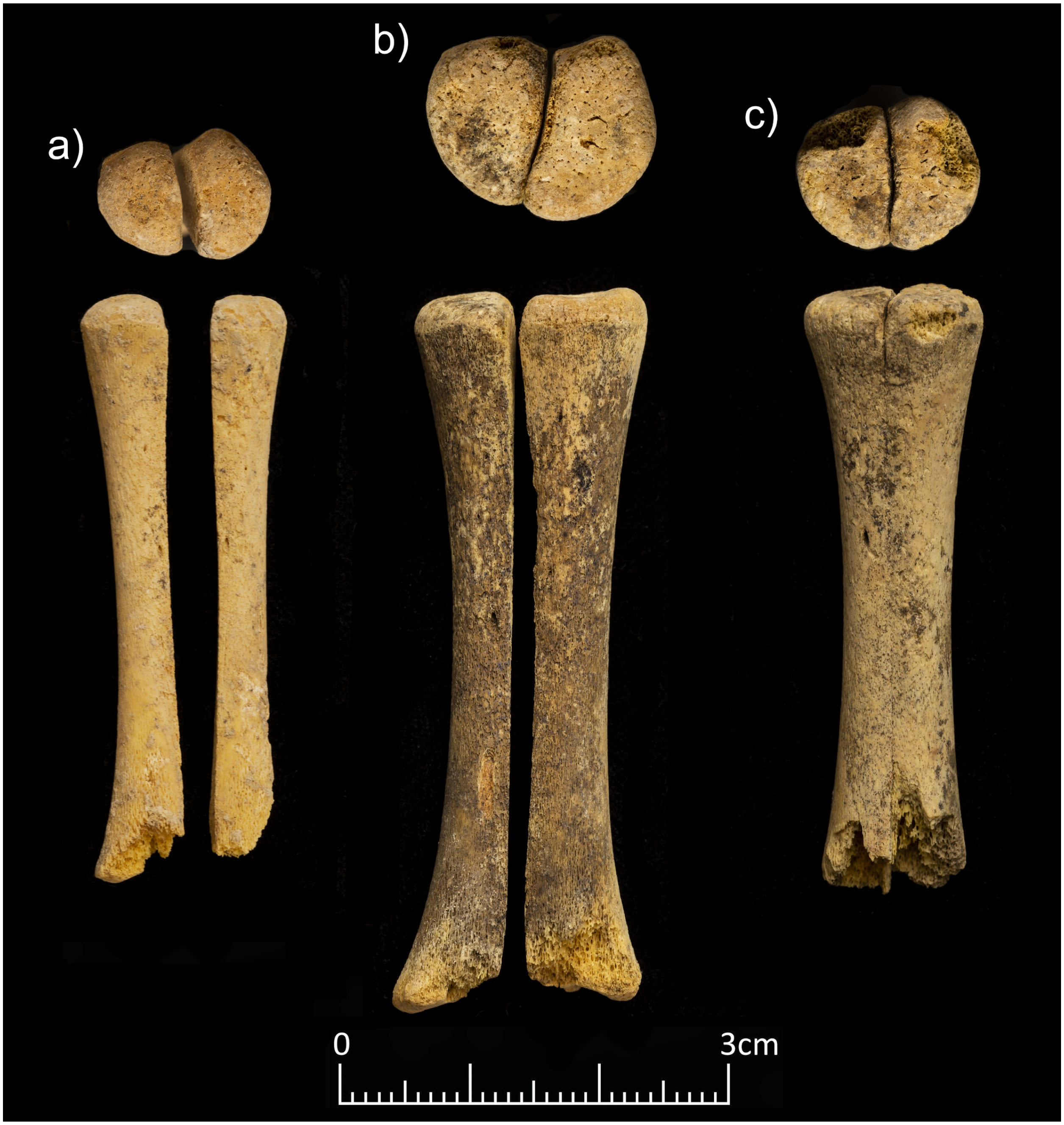

Fig. S6. Perinatal and neonatal caprine bones from Ganj Dareh. A) Foetal (stage I) metatarsals III and V with minimal indication of fusion between the bones from Phase A1.4; B) foetal metatarsals III and I with indication that fusion had started in the centre of the diaphysis (stage II) from Phase D.4; C) fused metatarsals from III and IV of a neonate from Phase D.1.

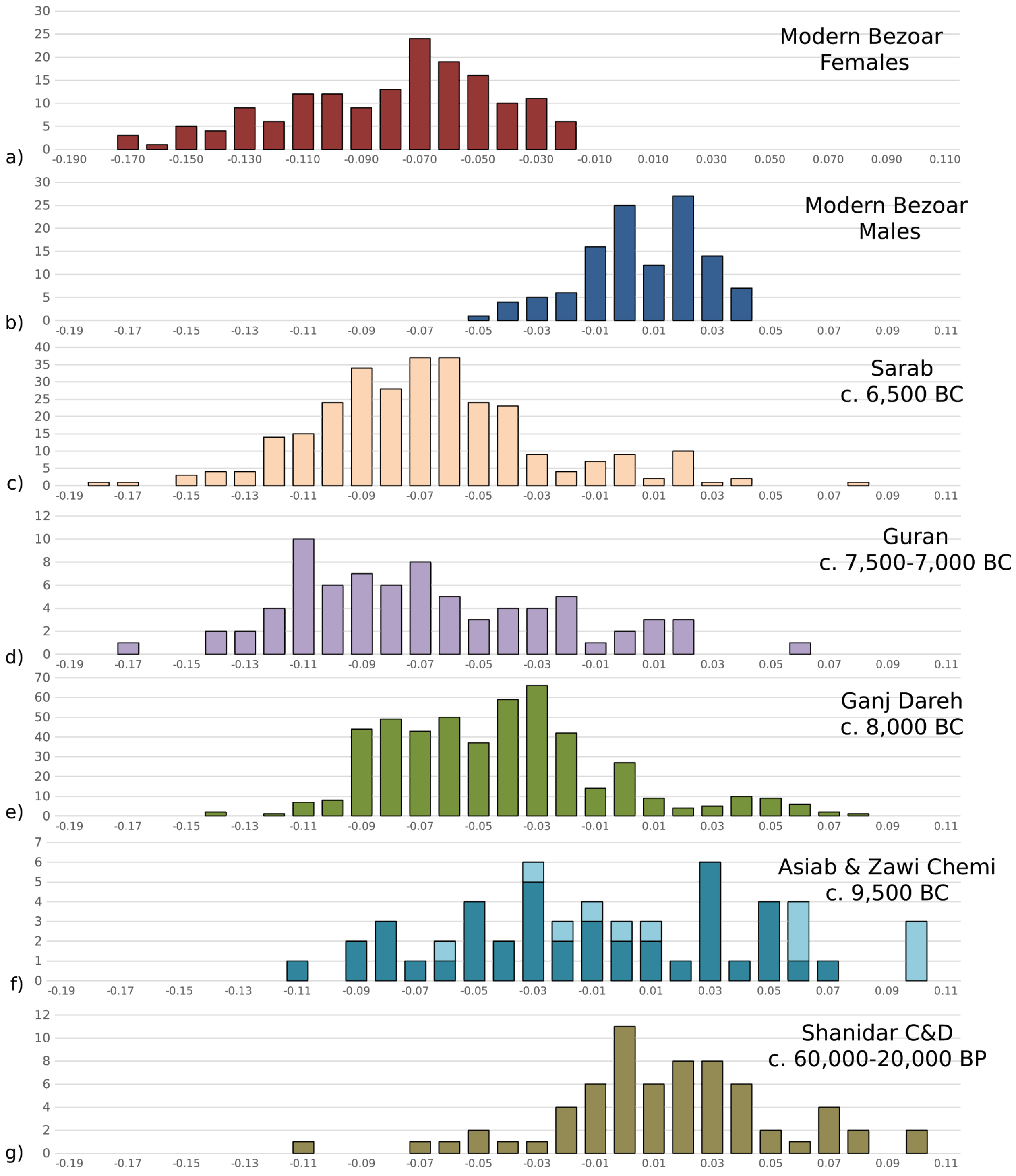

Fig. S7. Log normalized metric data of fused and fusing bones of modern wild and ancient goats from upland Zagros. a) Modern males; b) Modern females; c) Sarab c. 6,500 BC; d) Tepe Guran c. 7,500-7,000 BC; e) Ganj Dareh c. 8,000 BC; f) Asiab (dark blue) and Zawi Chemi (light blue) c. 9,500 BC; g) Shanidar Levels C and D c. 60,000-20,000 BP. LSI formula: x=log(y/z) where x= LSI value, y=measurement of specimen, z=measurement of standard. Standard used

is a modern male bezoar from Iraqi Kurdistan, part of the Zoology collections at the Field Museum of Natural History, Catalog number 44466. Measurements included (following von den Driesch 1979) with standard measurement: 1st phalanx GL 44.29 mm, 2nd phalanx GL 30.31 mm, Astragalus GLl 21.89 mm, Calcaneus Dp 24.11 mm, Humerus Bd 39.47 mm, Metacarpal Bd 34.18 mm, Metatarsal Bd 29.61 mm, Radius Bp 39.07 mm, Radius Dd 26.31 mm, Tibia Dd 23.16 mm. Metrics from early excavations at sites measured by Zeder and do not include elements from more recent excavations.

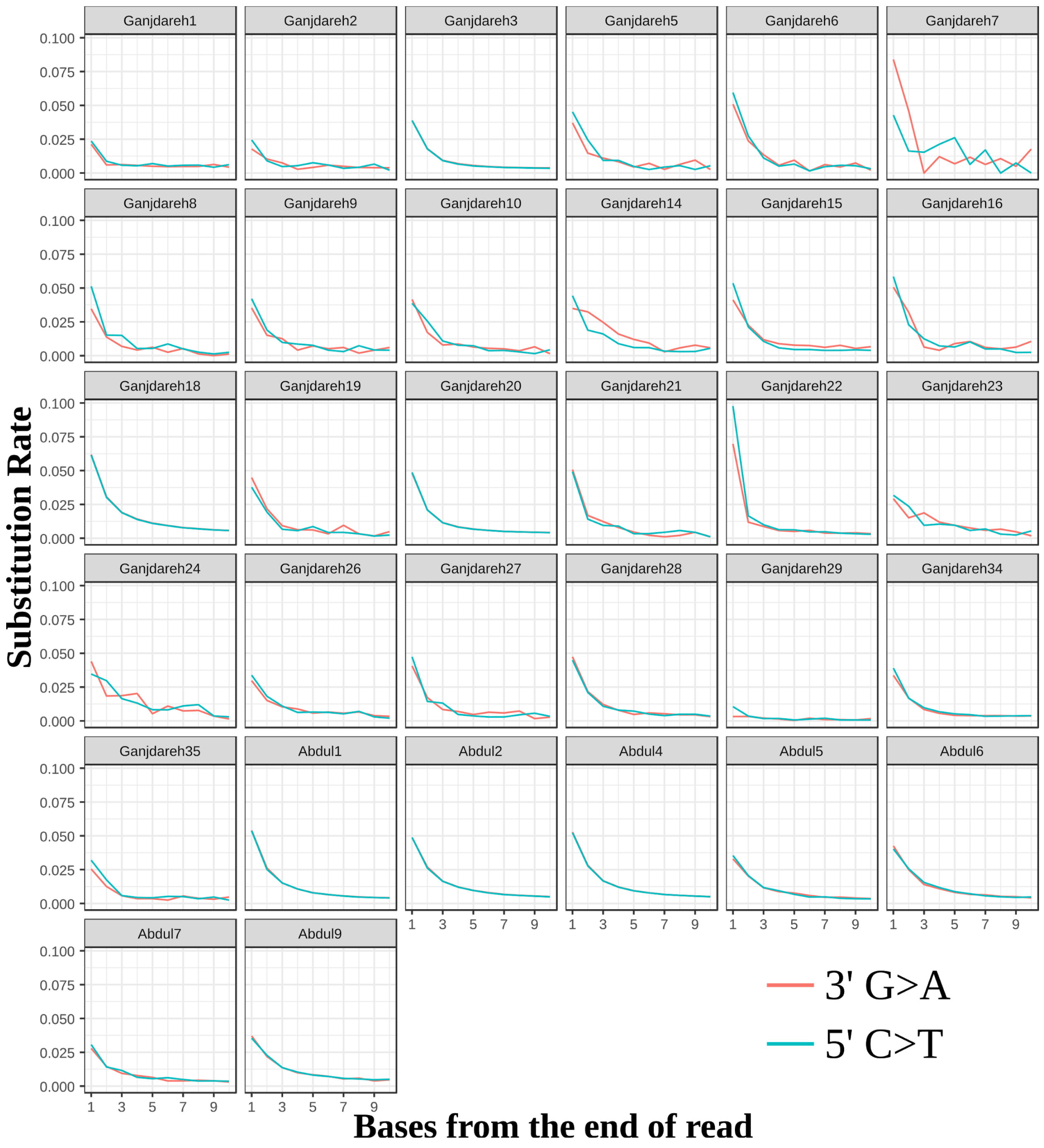

Fig. S8: 5’ C>T and 3’ G>A substitution rates of Neolithic Zagros goat samples sequenced here, relative to the distance from the read end.

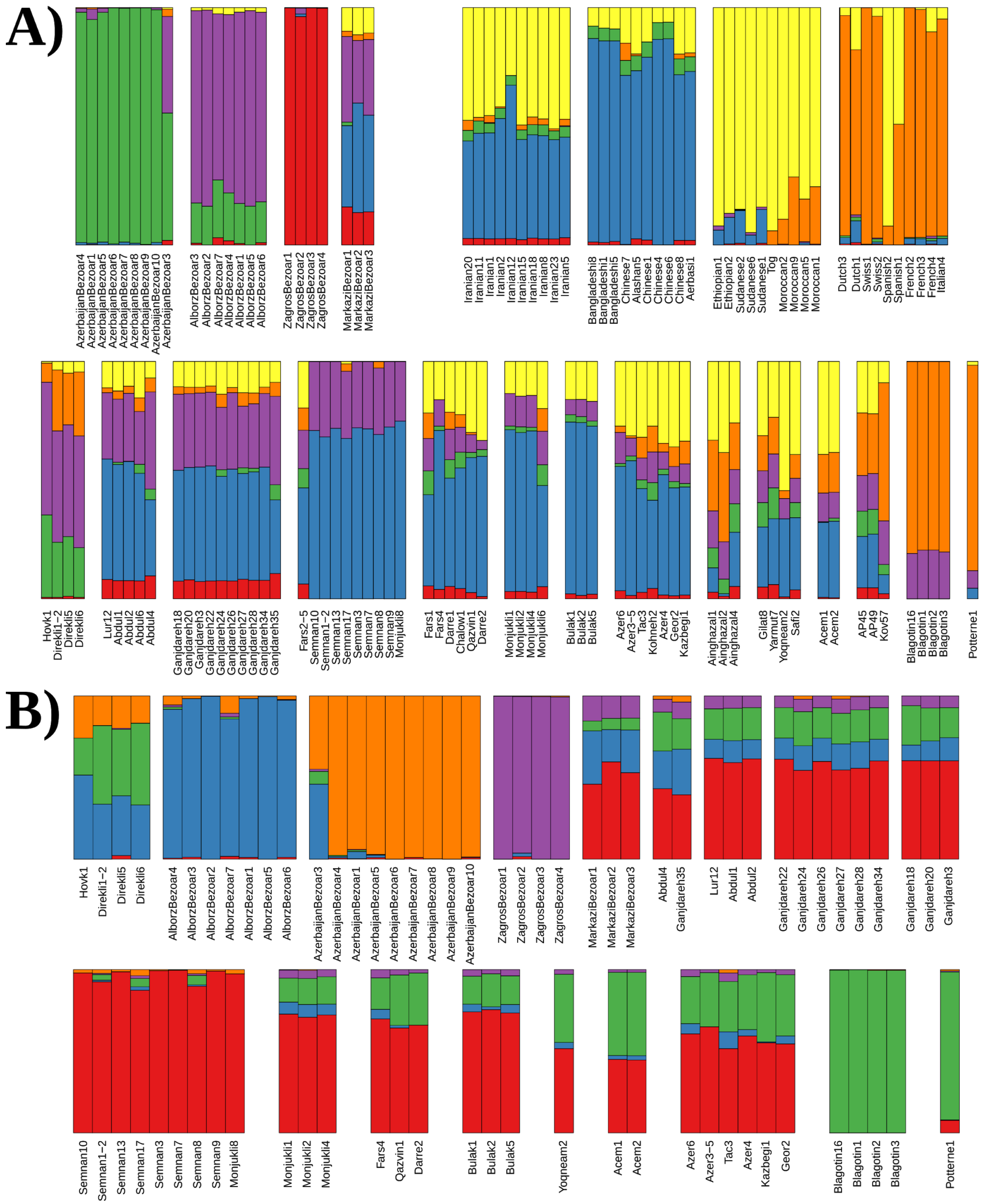

Fig. S9. PCAngsd estimation of ancestry proportions of A) modern bezoar, domestic, and ancient goat genomes with >0.01X mean coverage, and B) modern bezoar and ancient goat genomes with >0.1X mean coverage.

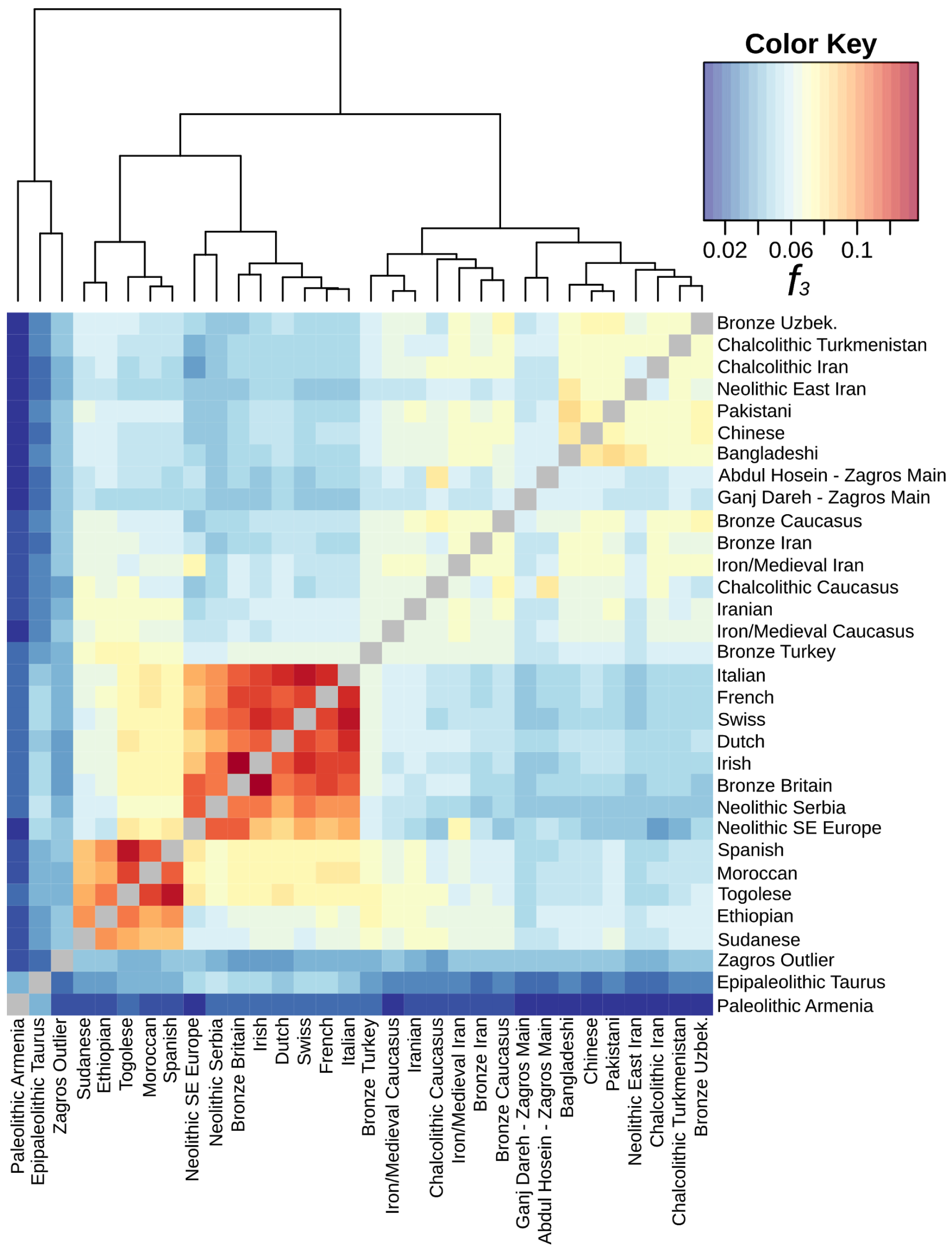

Fig. S10. Heatmap of outgroup f_3 values for groups, with CN Zagros and all Levantine groups removed due to low SNP coverage. Modern wild bezoar pooled as a single group was used as the outgroup.

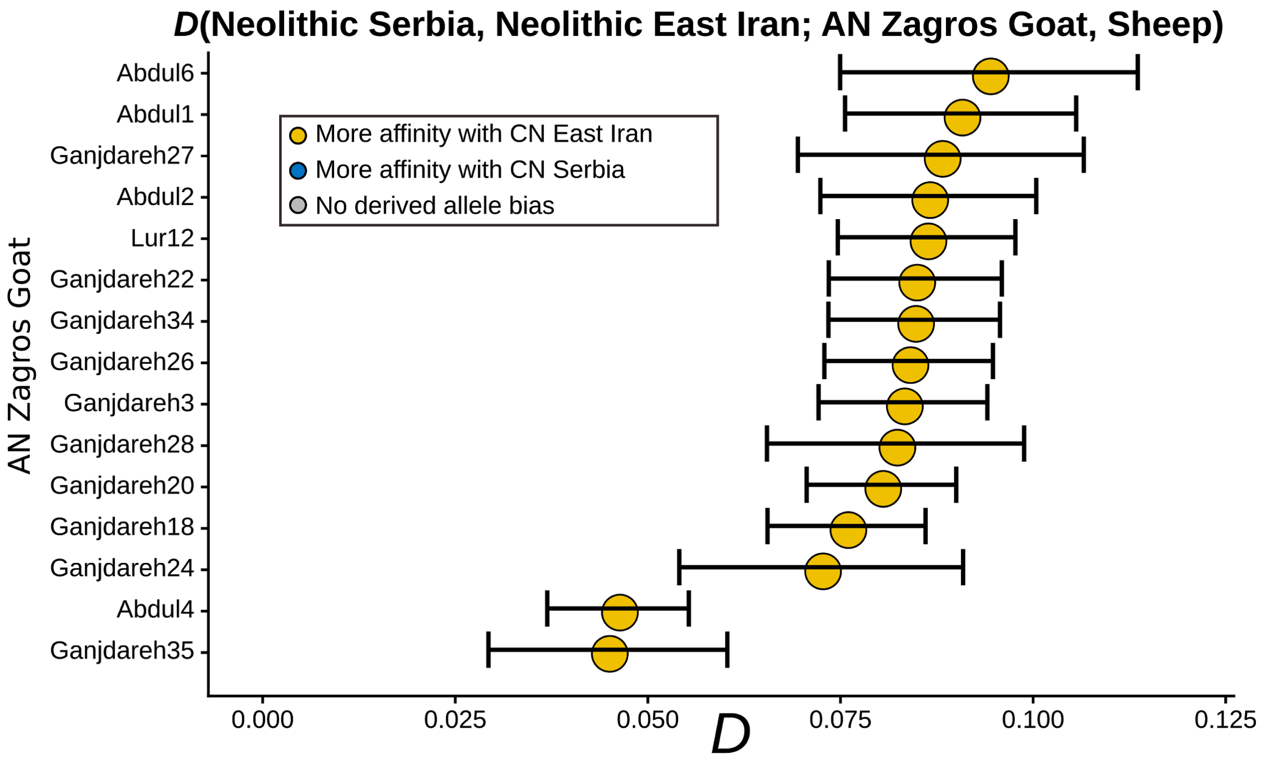

Fig. S11. *D* statistic test of relative affinity of an Aceramic Neolithic (AN) Zagros Goat genome to a representative Ceramic Neolithic (CN) East Iran genome (Semnan3) or CN Serbia genome (Blagotin3), using the test *D*(CN Serbia, CN East Iran; AN Zagros Goat, Sheep). Significant ($Z \geq 3$) positive values indicate a greater degree of derived allele sharing between AN Zagros goat and CN East Iran, compared to AN Zagros goat and CN Serbia.

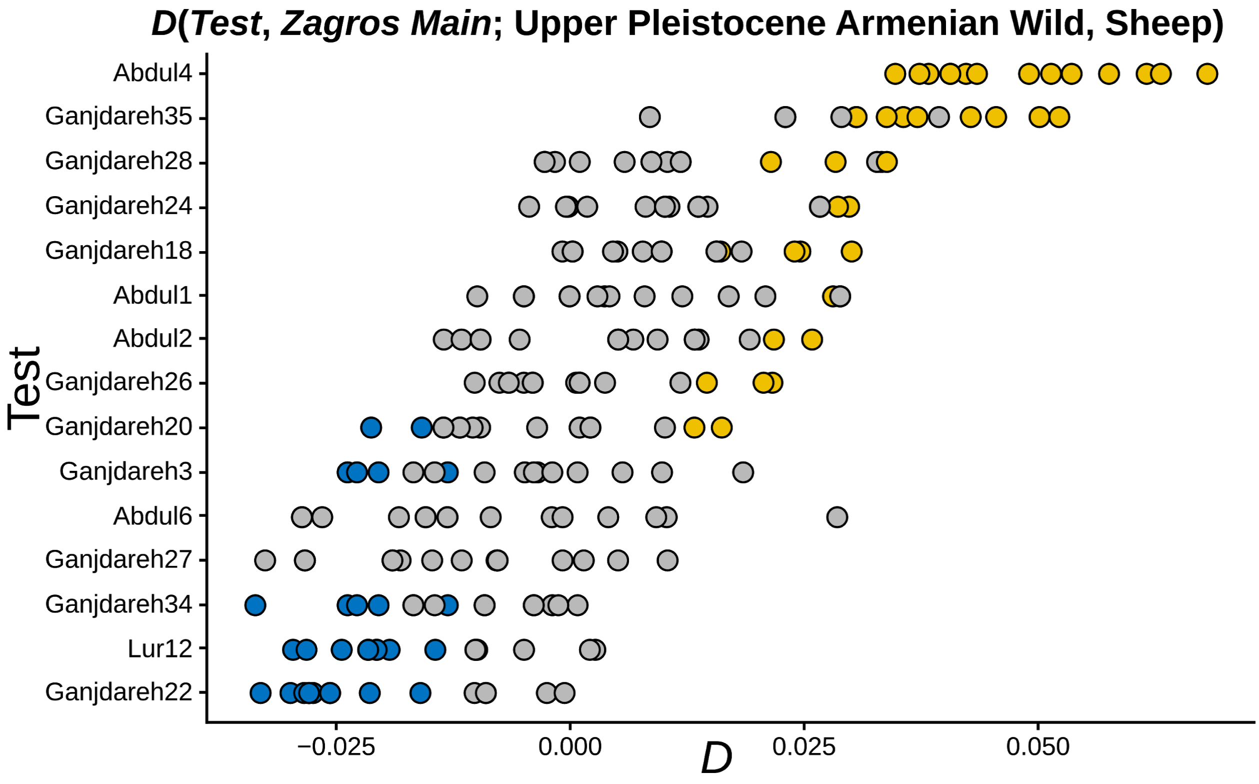

Fig. S12. *D* statistic of the form *D*(Test, *Zagros Main*; Upper Pleistocene Armenian Wild (Hovk1), Sheep), with *Zagros Main* genomes cycled through for each Test. Significant (Z ≥ 3) positive values of *D* in yellow indicate that the Test shares more derived alleles with the Upper Pleistocene Wild (Hovk1) than the Zagros Main genome and Hovk1 do.

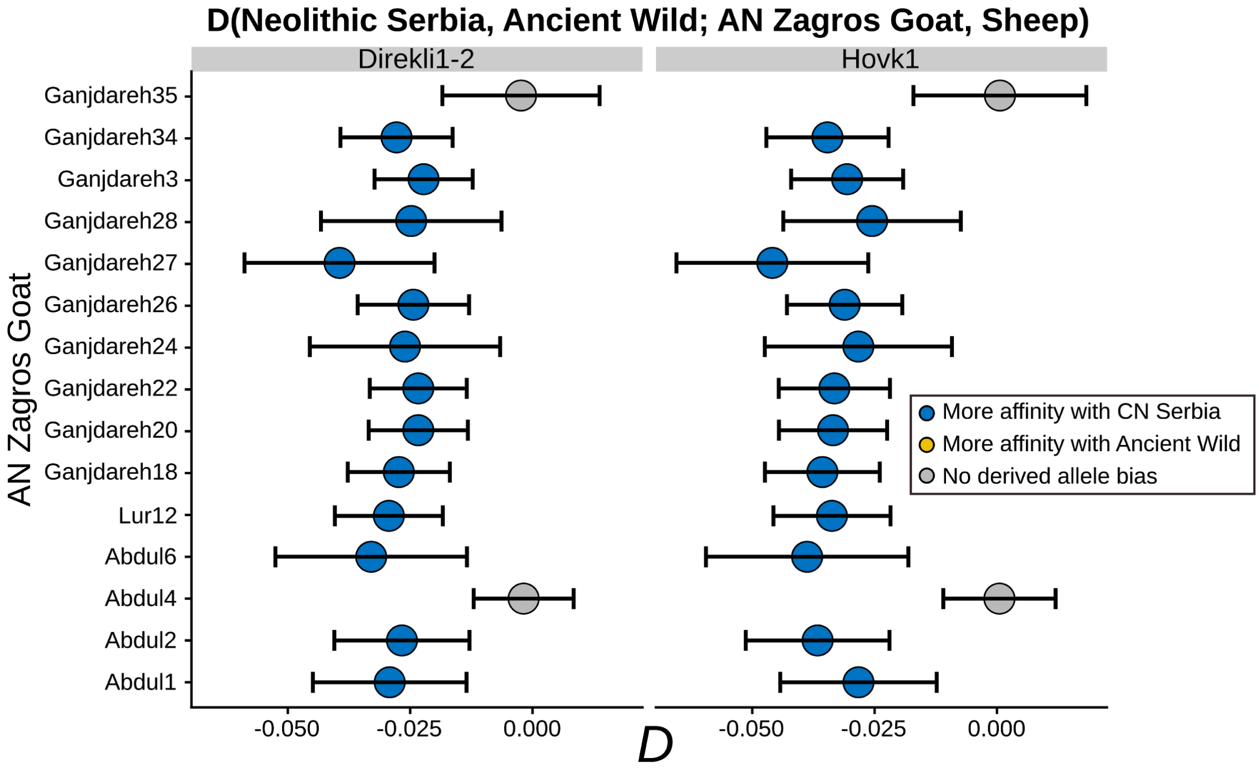

Fig. S13. *D* statistic test of relative affinity of an Aceramic Neolithic (AN) Zagros goat genome to a representative Ceramic Neolithic (CN) Neolithic East Serbian (Blagotin3) or ancient wild genome (Late Pleistocene Armenian, Hovk1, or Epipaleolithic Anatolian, Direkli1-2), using the test *D*(CN Serbia, Ancient Wild; AN Zagros Goat, Sheep). Significant ($Z \leq 3$) negative values indicate a greater degree of derived allele sharing between AN Zagros goat and CN East Serbia, compared to AN Zagros goat and the ancient wild genome. Non-significant values indicate no bias in the sharing of derived alleles of the AN Zagros goat with either the Neolithic Serbian or Ancient Wild genome.

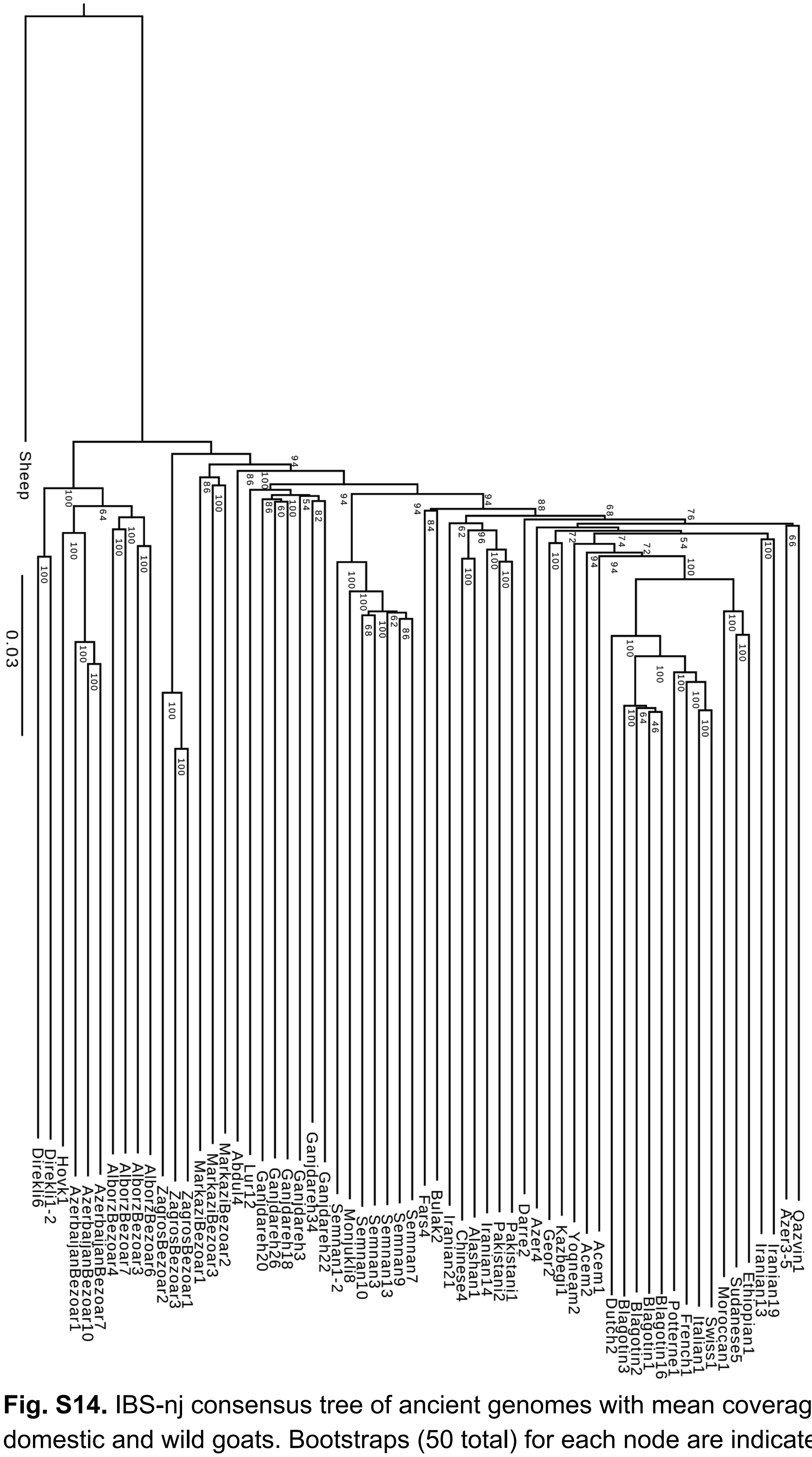

Fig. S14. IBS-nj consensus tree of ancient genomes with mean coverage >1X, and modern domestic and wild goats. Bootstraps (50 total) for each node are indicated. Tree is rooted on Sheep.

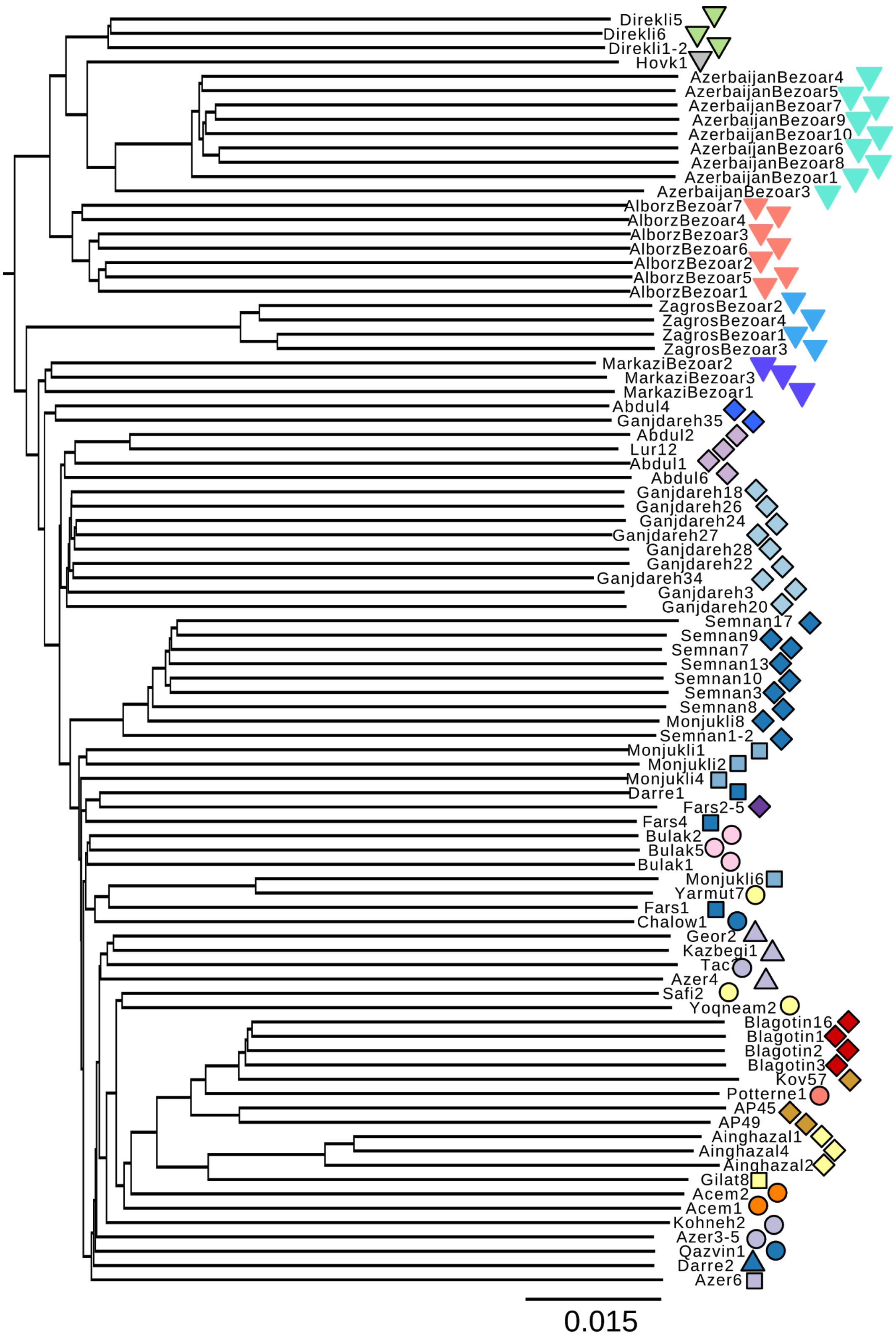

Fig. S15. IBS-nj tree of ancient genomes with mean coverage >0.01X, and modern bezoar genomes. Tree is rooted on Sheep and is not shown.

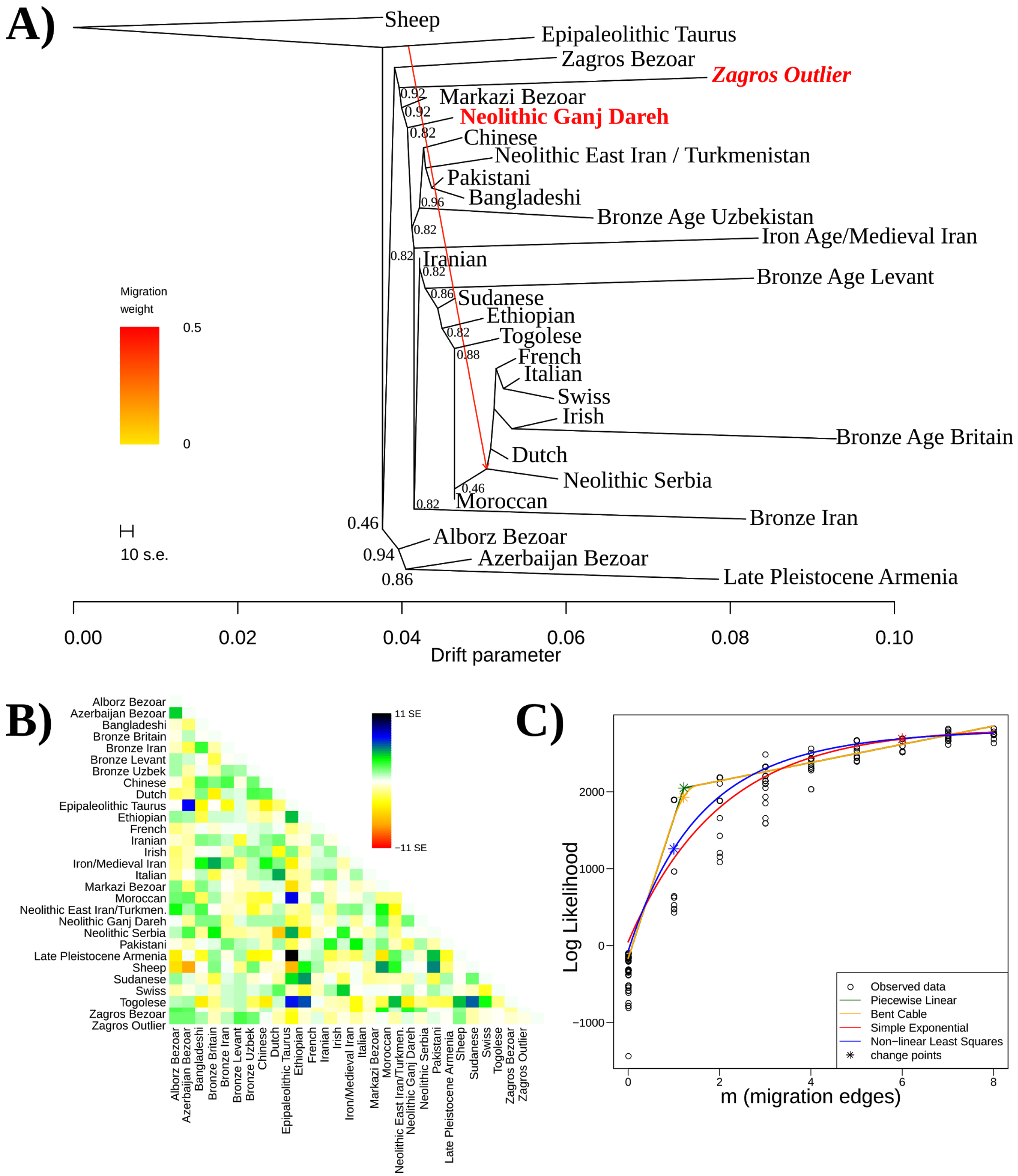

Fig. S16. Treemix results. A) Treemix of ancient and modern genomes with >2X coverage and residuals, *m*=1. Bootstrap support values for nodes less than 1 are displayed. B) Residual values for *m*=1. C) optM linear models of log-likelihood changes with number of migration edges.

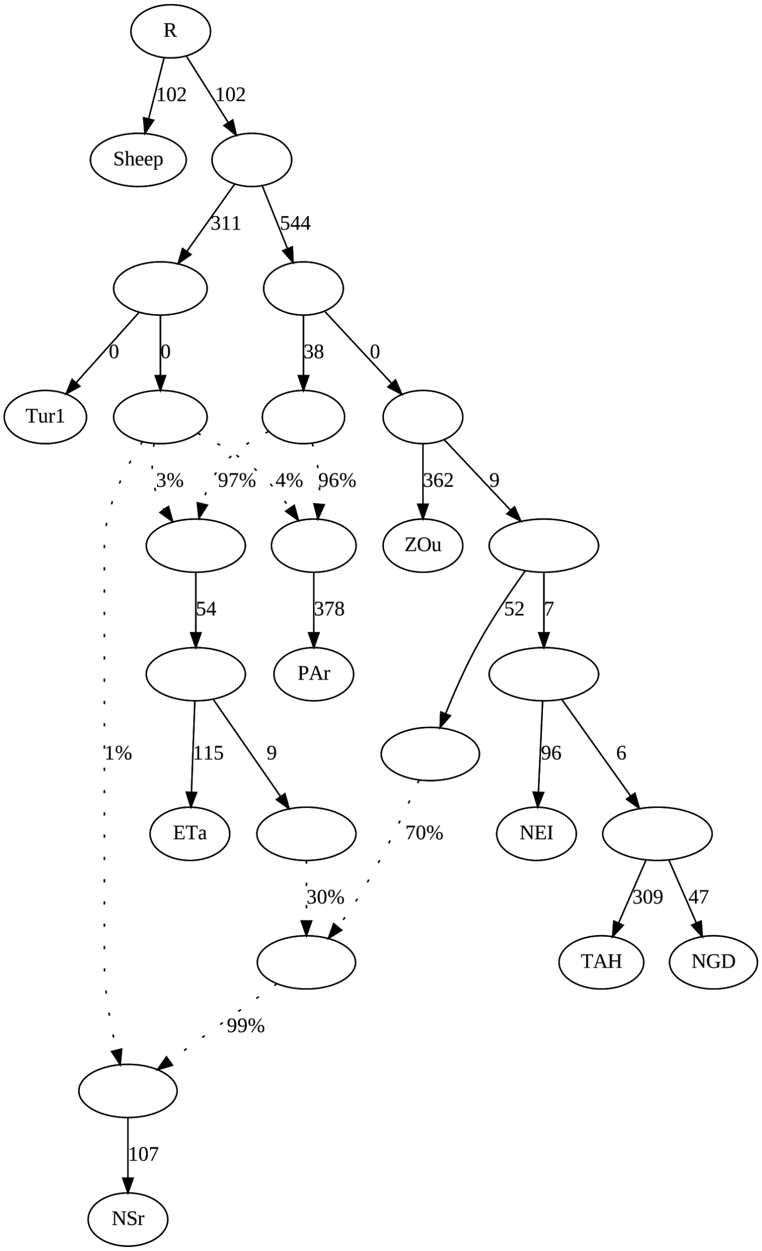

Fig. S17. Fitted qpGraph model used in Figure 5, with drift edges. **Key:** ZOu = Zagros Outlier; NGD = *Zagros Main* Ganj Dareh; TAH = *Zagros Main* Tepe Abdul Hosein; NEI = Ceramic Neolithic East Iran / Turkmenistan; NSr = Ceramic Neolithic Serbia; ETa = Epipaleolithic Taurus; PAr = Late Pleistocene Armenia; Tur1 = *Capra caucasica*.

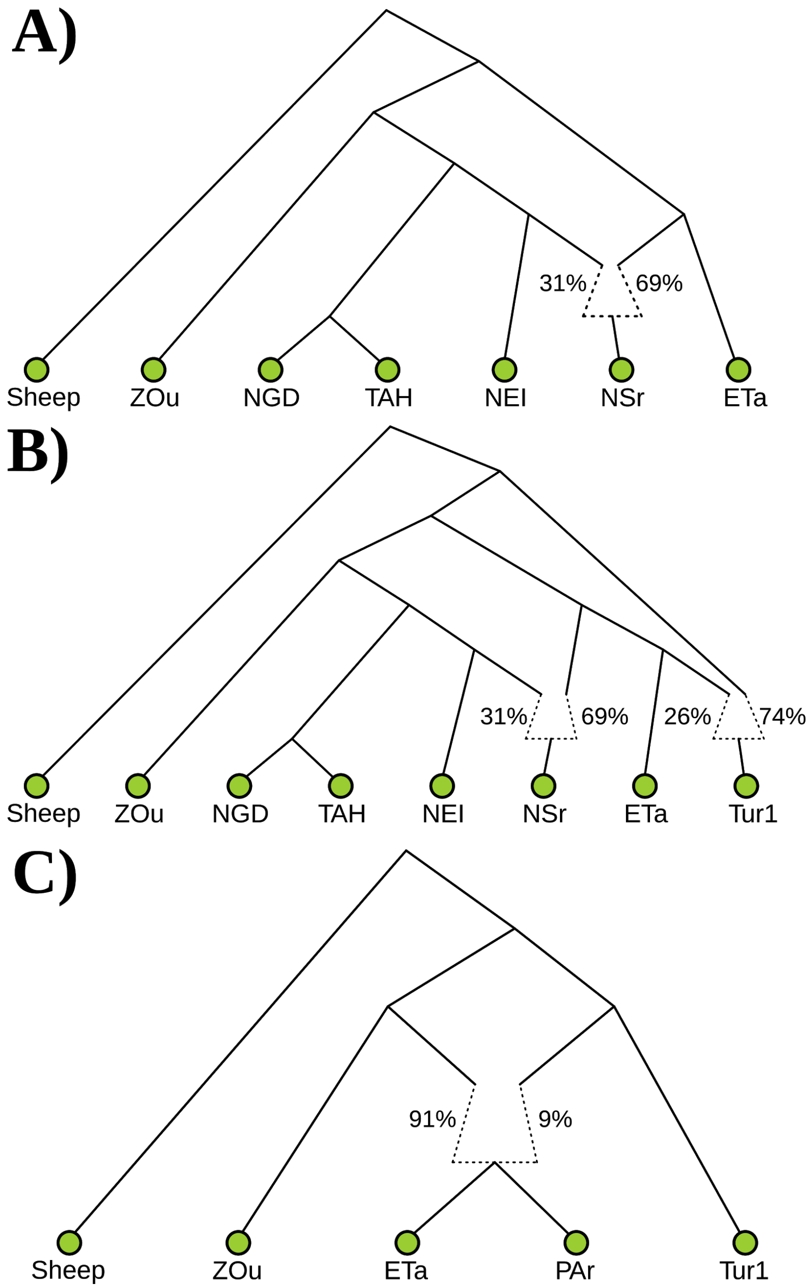

Fig. S18. Selected qpbrute graph results. A) qpbrute graph with highest pairwise *K* values, for the addition of NSr to the single fitting graph of ZOu, NGD, TAH, NEI. B) Addition of Tur1 to the graph in A). C) Best fitting graph of ZOu, Tur1, ETa, PAr. **Key:** ZOu = Zagros Outlier; NGD = *Zagros Main* Ganj Dareh; TAH = *Zagros Main* Tepe Abdul Hosein; NEI = Ceramic Neolithic East Iran / Turkmenistan; NSr = Ceramic Neolithic Serbia; ETa = Epipaleolithic Taurus; PAr = Late Pleistocene Armenia; Tur1 = *Capra caucasica*.

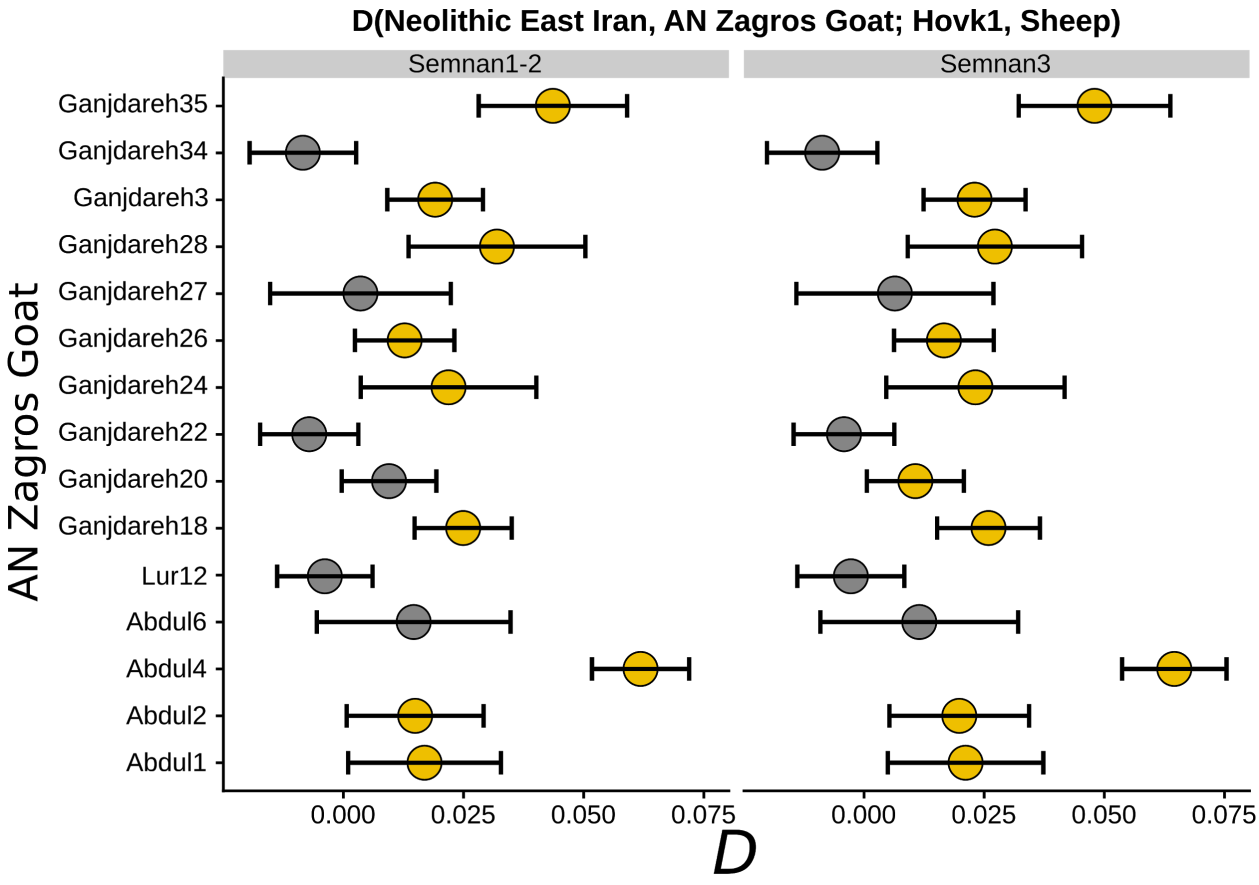

Fig. S19. *D* statistic test of relative affinity of the Late Pleistocene Armenian wild goat, Hovk1, to either an Aceramic Neolithic (AN) Zagros goat genome or a representative Neolithic East Iran, using the test *D*(Neolithic East Iran, AN Zagros Goat; Ancient Wild, Sheep). Significant ($Z \geq 3$) positive values indicate a greater degree of derived allele sharing between Hovk1 and a AN Zagros goat, compared to Hovk1 and a Neolithic East Iran goat. Non-significant values indicate no bias in the sharing of derived alleles of Hovk1 with either the AN Zagros or Neolithic East Iran genome.

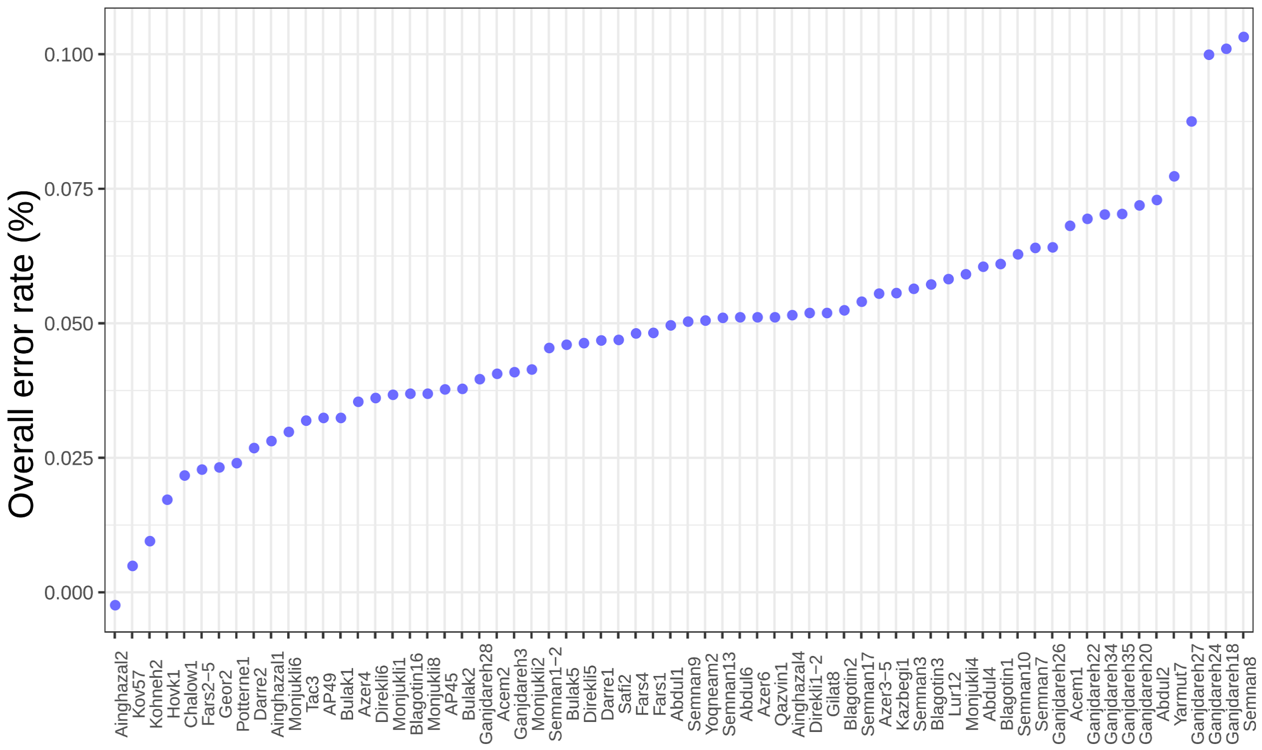

Fig. S20. Overall error rates of ancient genomes as estimated by the ANGSD “perfect genome” approach.

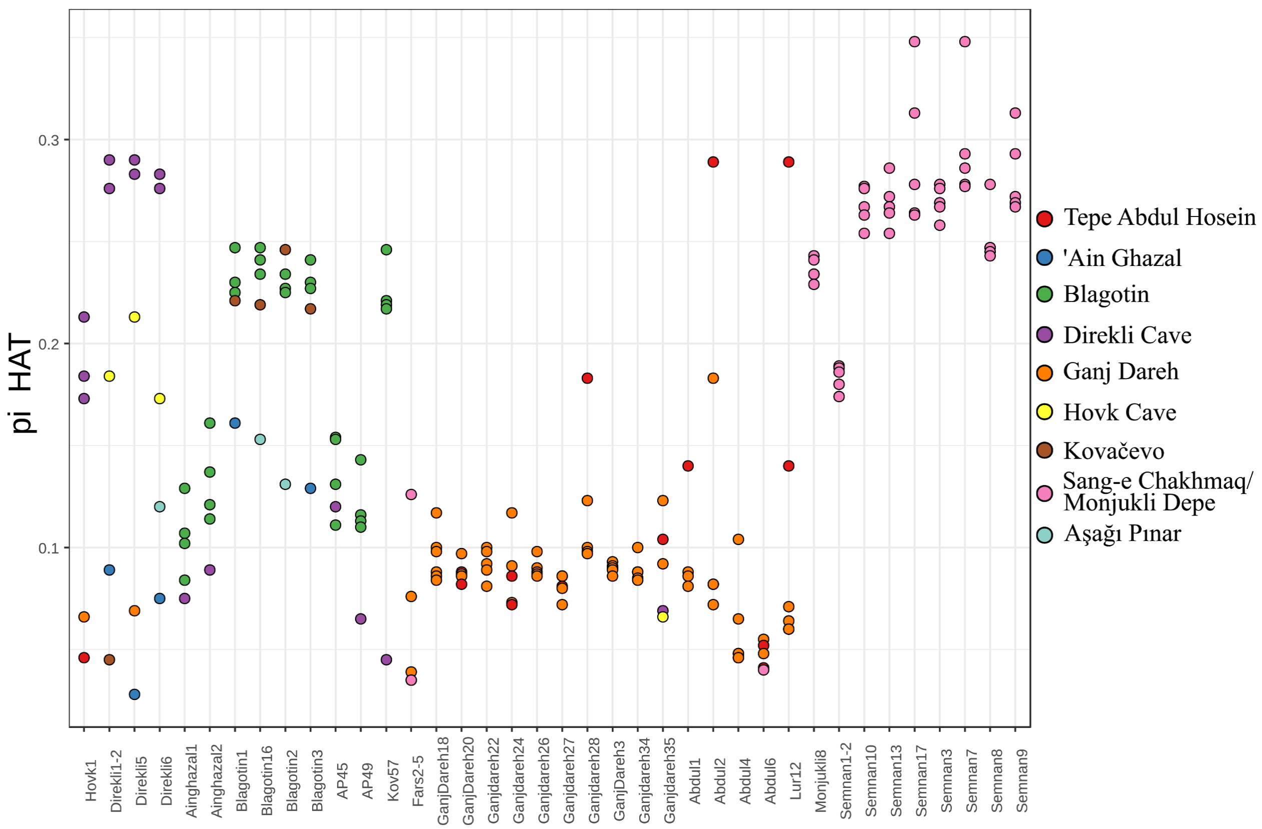

Fig. S21. Pairwise kinship coefficients among Neolithic genomes. For each sample, the top 5 pairwise coefficient values are displayed, coloured by their site of origin.

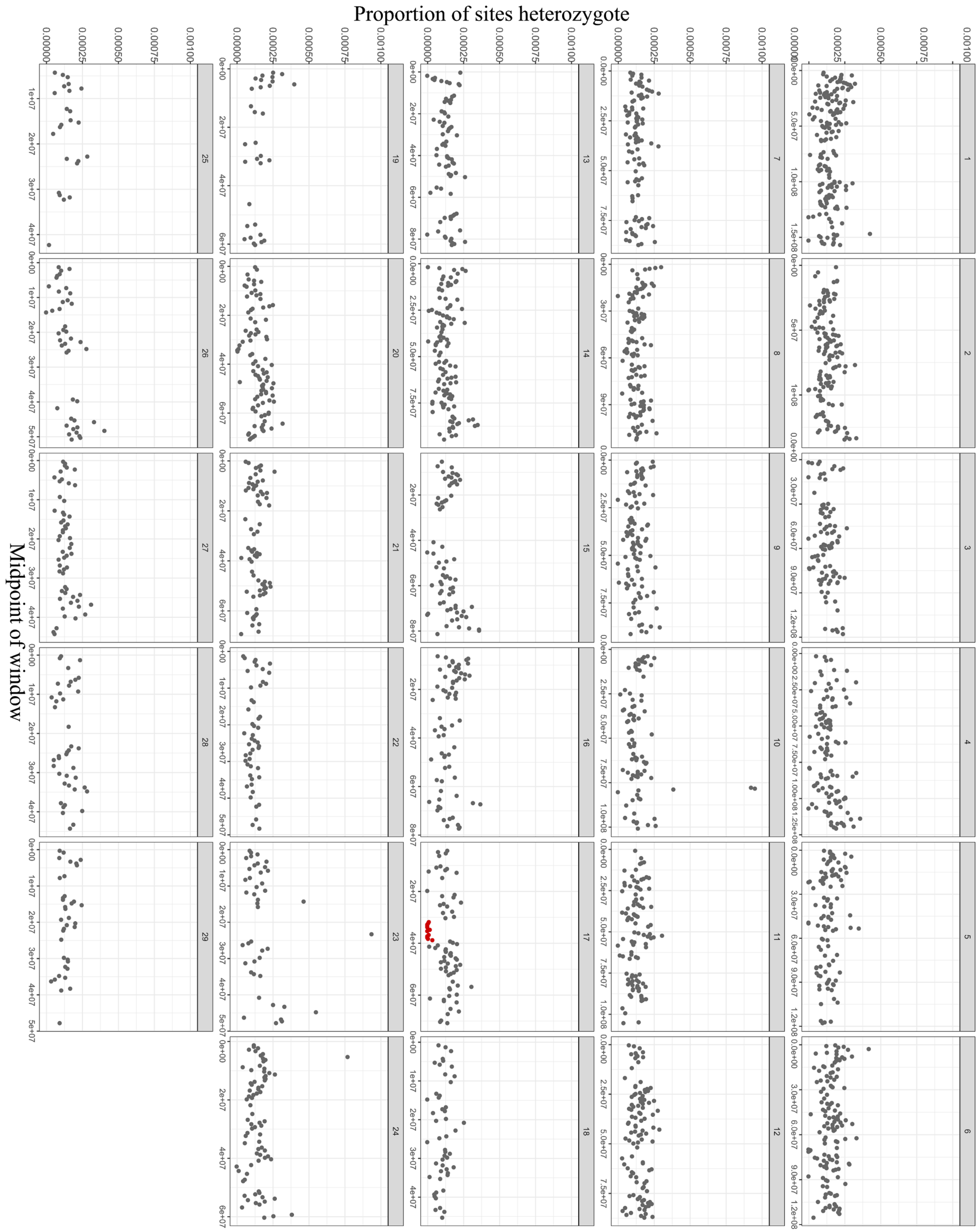

Fig. S22. Transversion heterozygosity rate of 50 kbp windows for Ganjdareh3 across the 29 goat autosomes. Windows assigned to long-ROH are displayed in red.

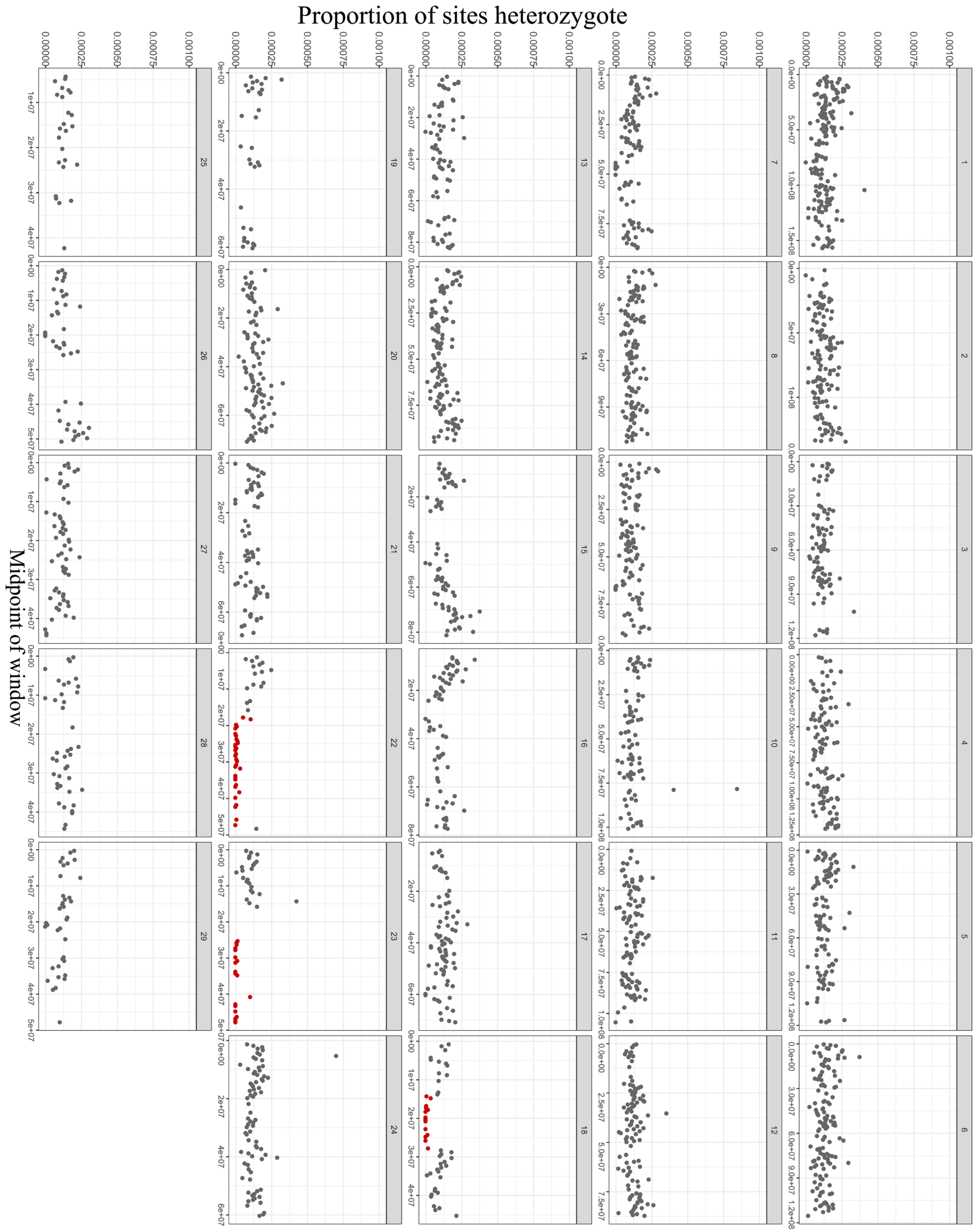

Fig. S23. Transversion heterozygosity rate of 50 kbp windows for Ganjdareh18 across the 29 goat autosomes. Windows assigned to long-ROH are displayed in red.

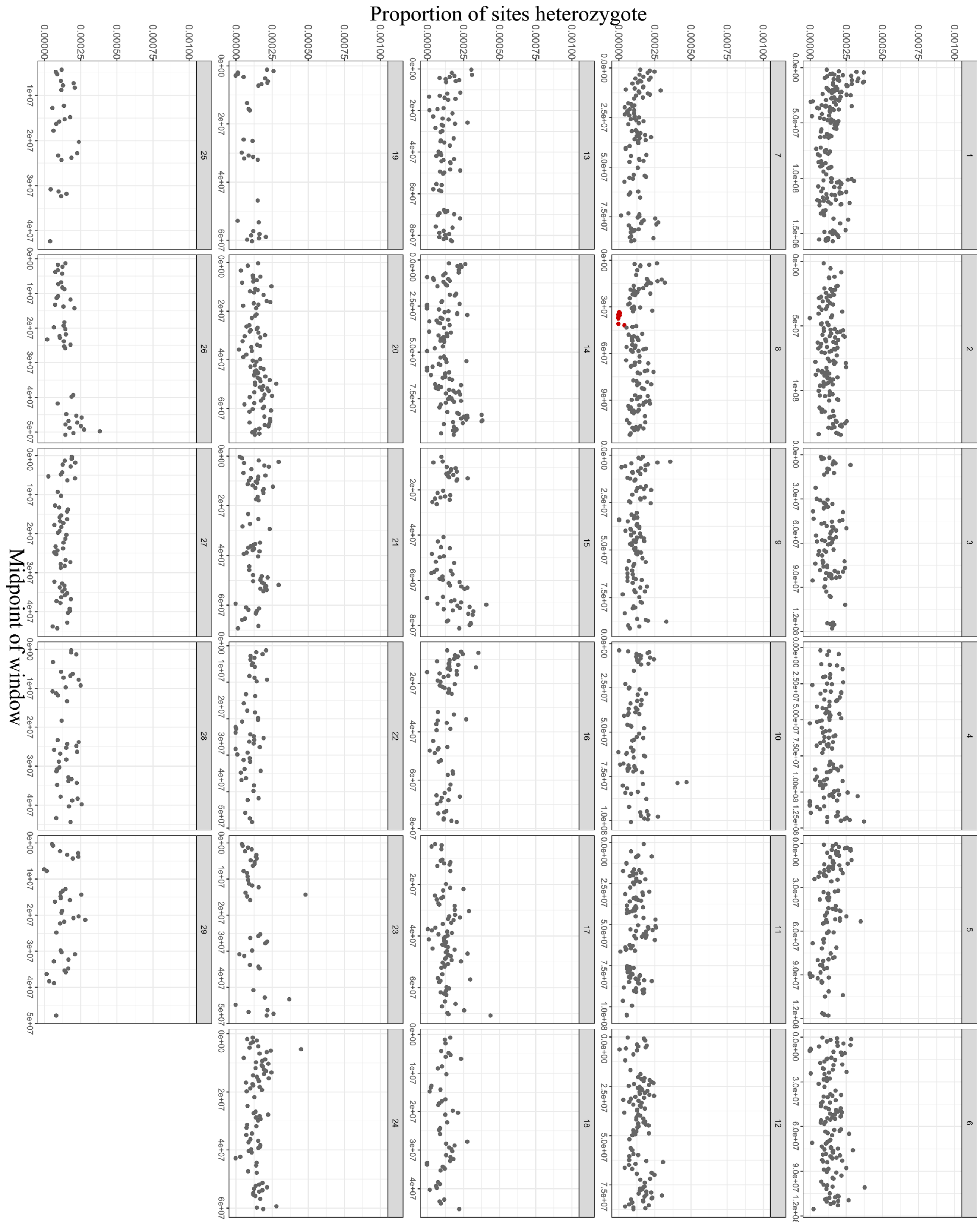

Fig. S24. Transversion heterozygosity rate of 50 kbp windows for Ganjdareh20 across the 29 goat autosomes. Windows assigned to long-ROH are displayed in red.

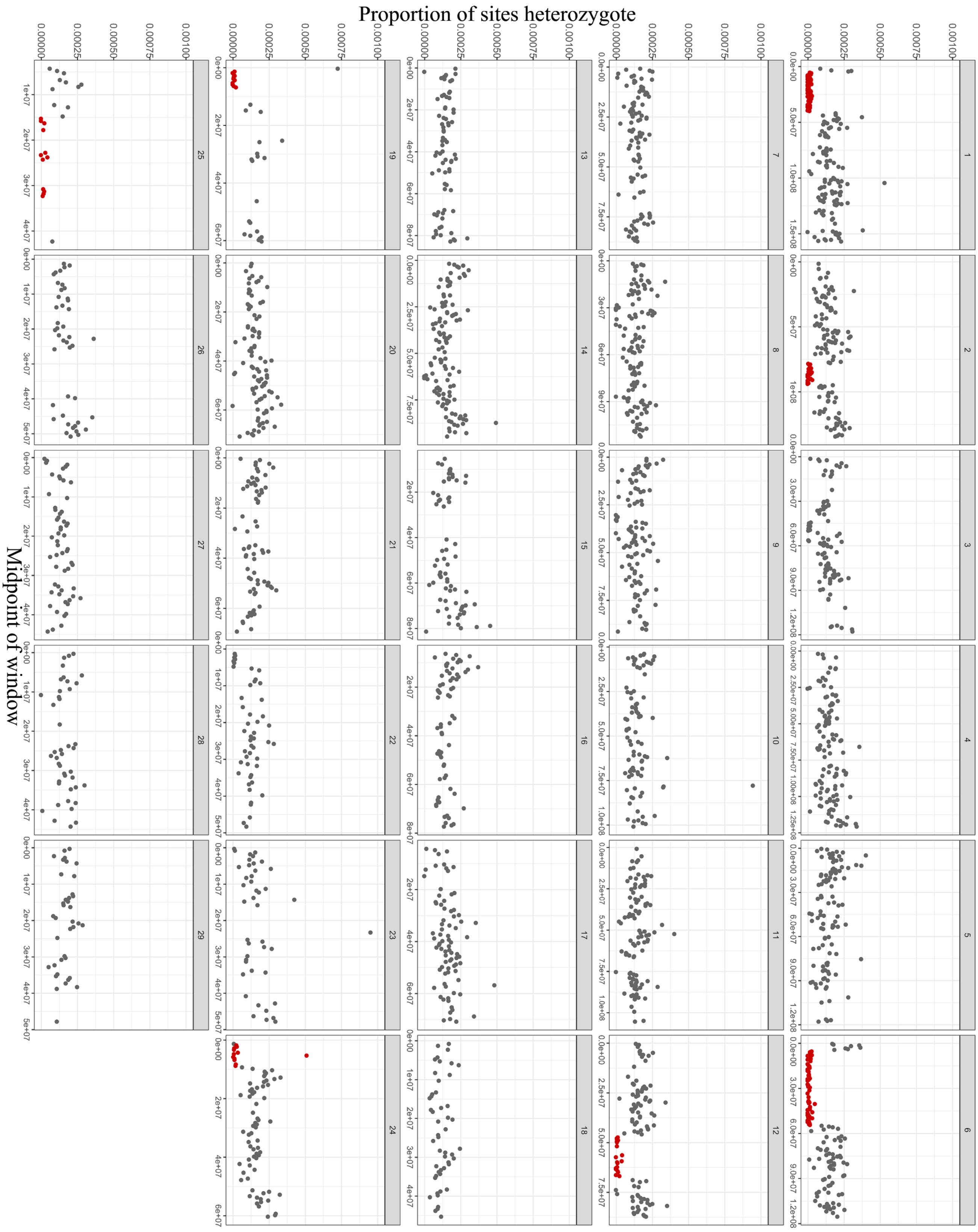

Fig. S25. Transversion heterozygosity rate of 50 kbp windows for Ganjdareh22 across the 29 goat autosomes. Windows assigned to long-ROH are displayed in red.

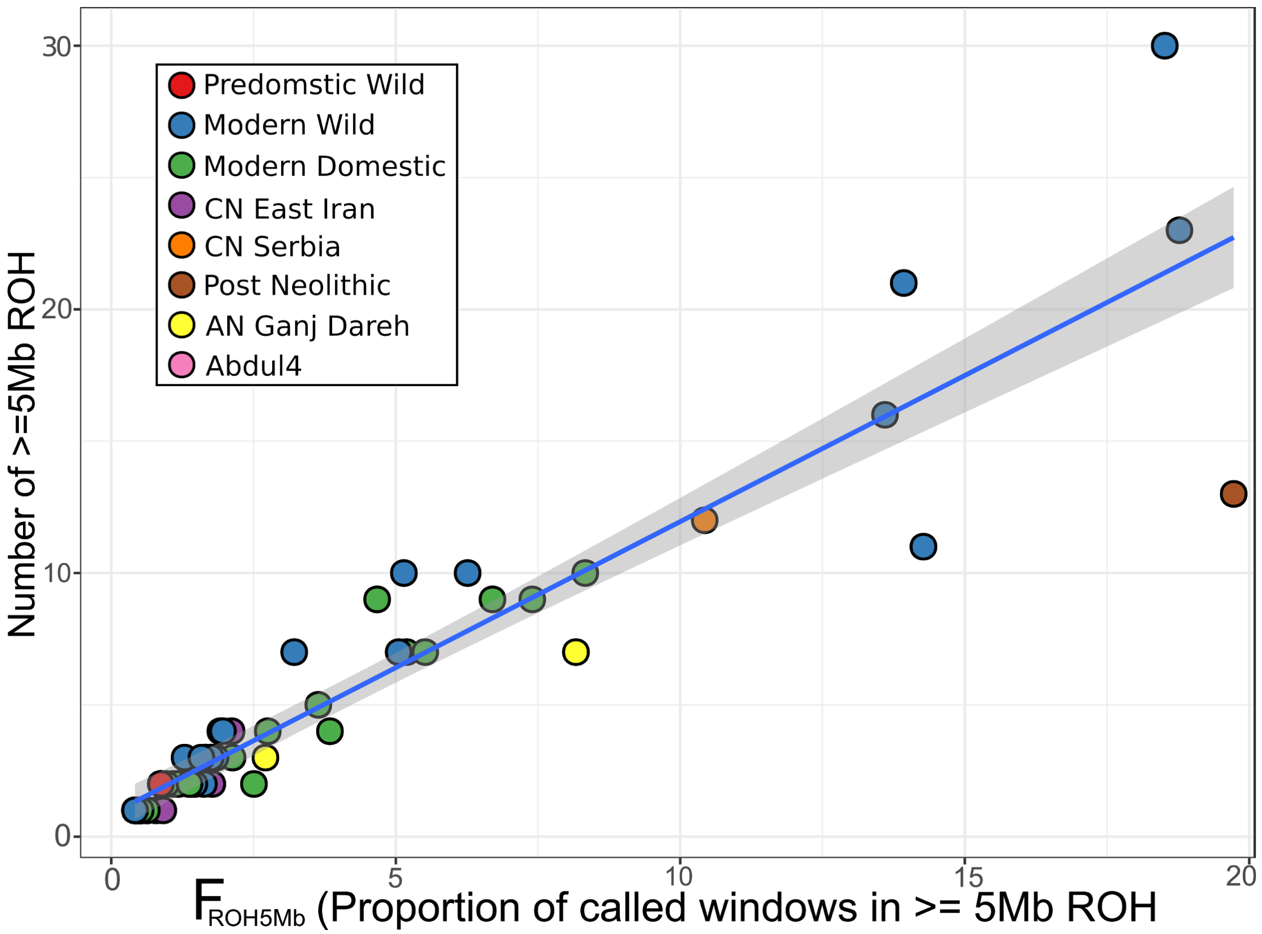

Fig. S26: Number of ≥ 5Mb ROH regions identified plotted against FROH5Mb for each sample, with a linear regression line added using the geom_smooth(method='lm') function of ggplot2 (Hadley, 2016) and excluding samples with no detected ≥ 5Mb ROH.

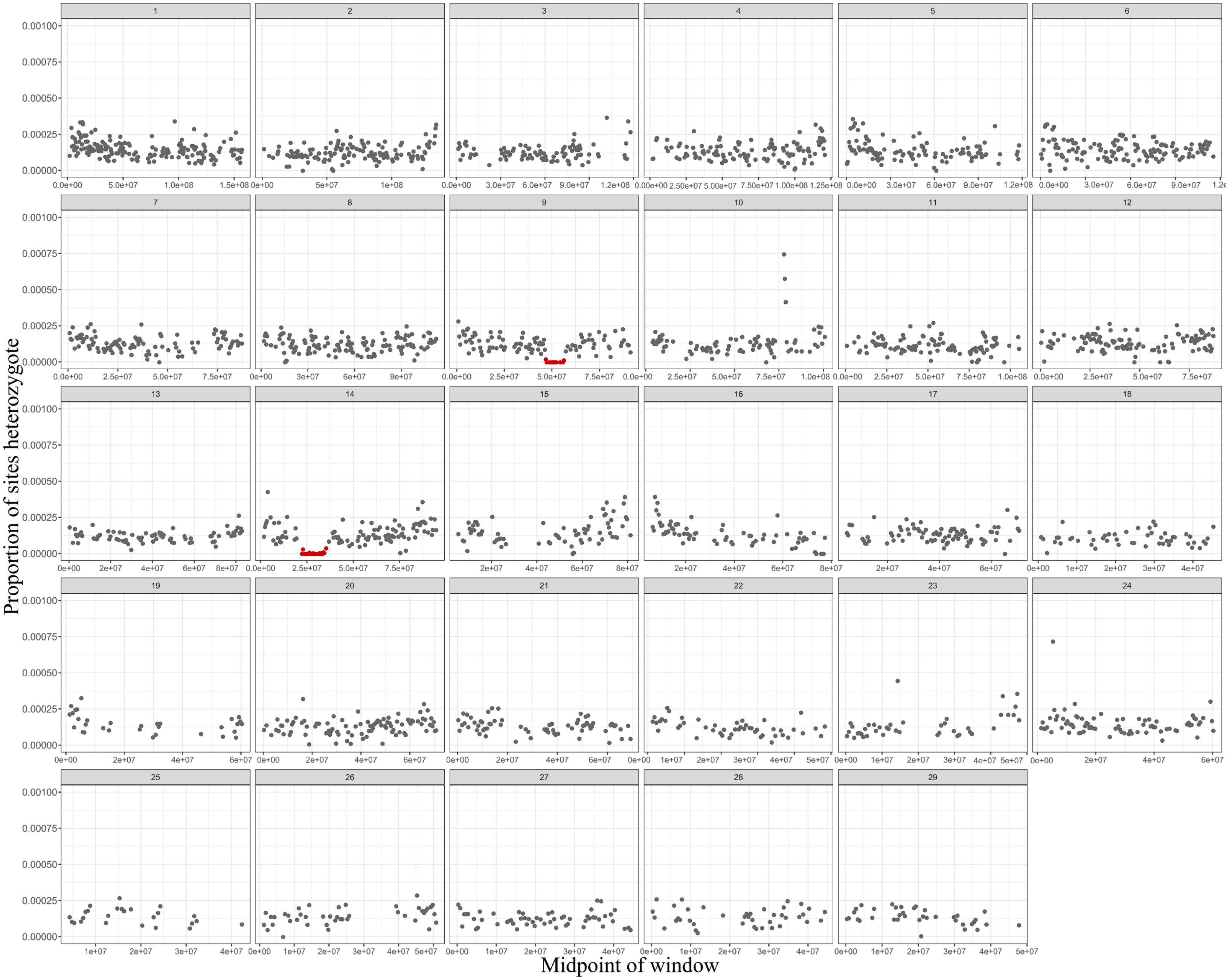

Proportion of sites heterozygote
Midpoint of window

Fig. S27. Transversion heterozygosity rate of 50 kbp windows for Abdul4 across the 29 goat autosomes. Windows assigned to long-ROH are displayed in red.

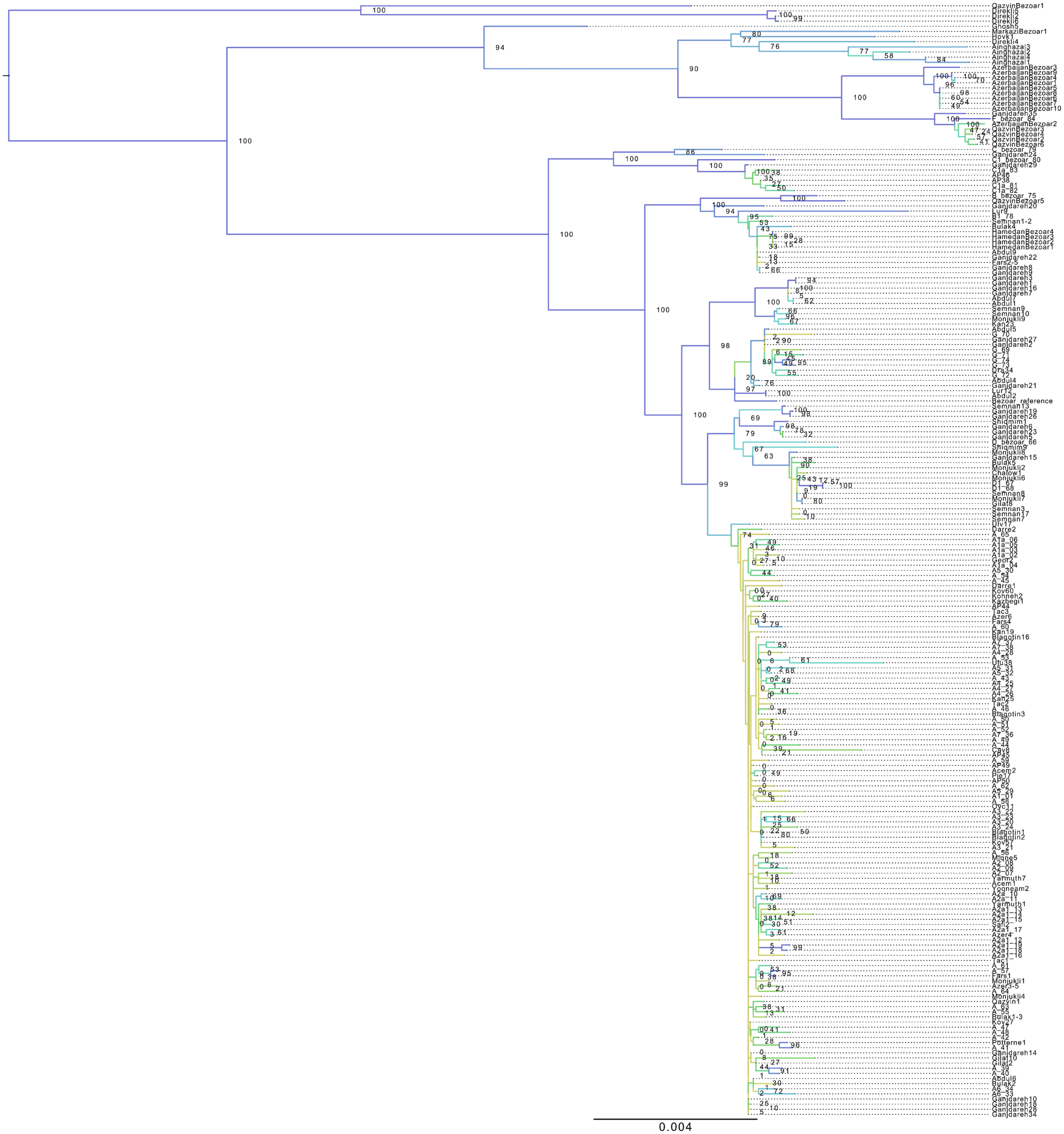

Fig. S28. Maximum Likelihood phylogeny with ancient and modern whole mitochondrial sequences. Branches coloured by their associated node's bootstrap support, which are also shown. The outgroup, Nubian ibex, is not shown.

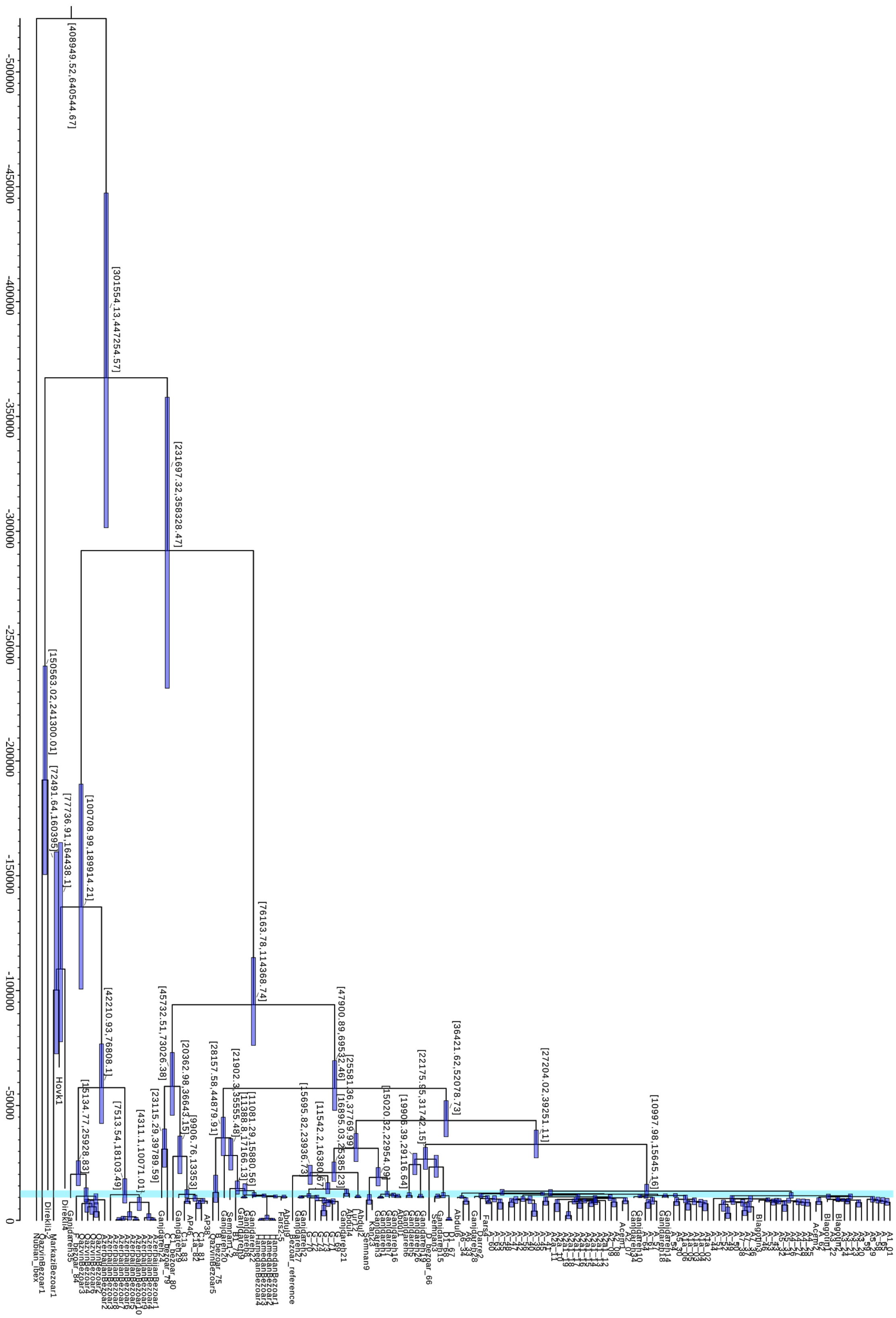

Fig. S29. Maximum clade credibility tree using Beast2. 95% HPD of node heights are shown (omitted for most basal node), displayed as text for key nodes. 11000-9500 BP indicated in cyan.

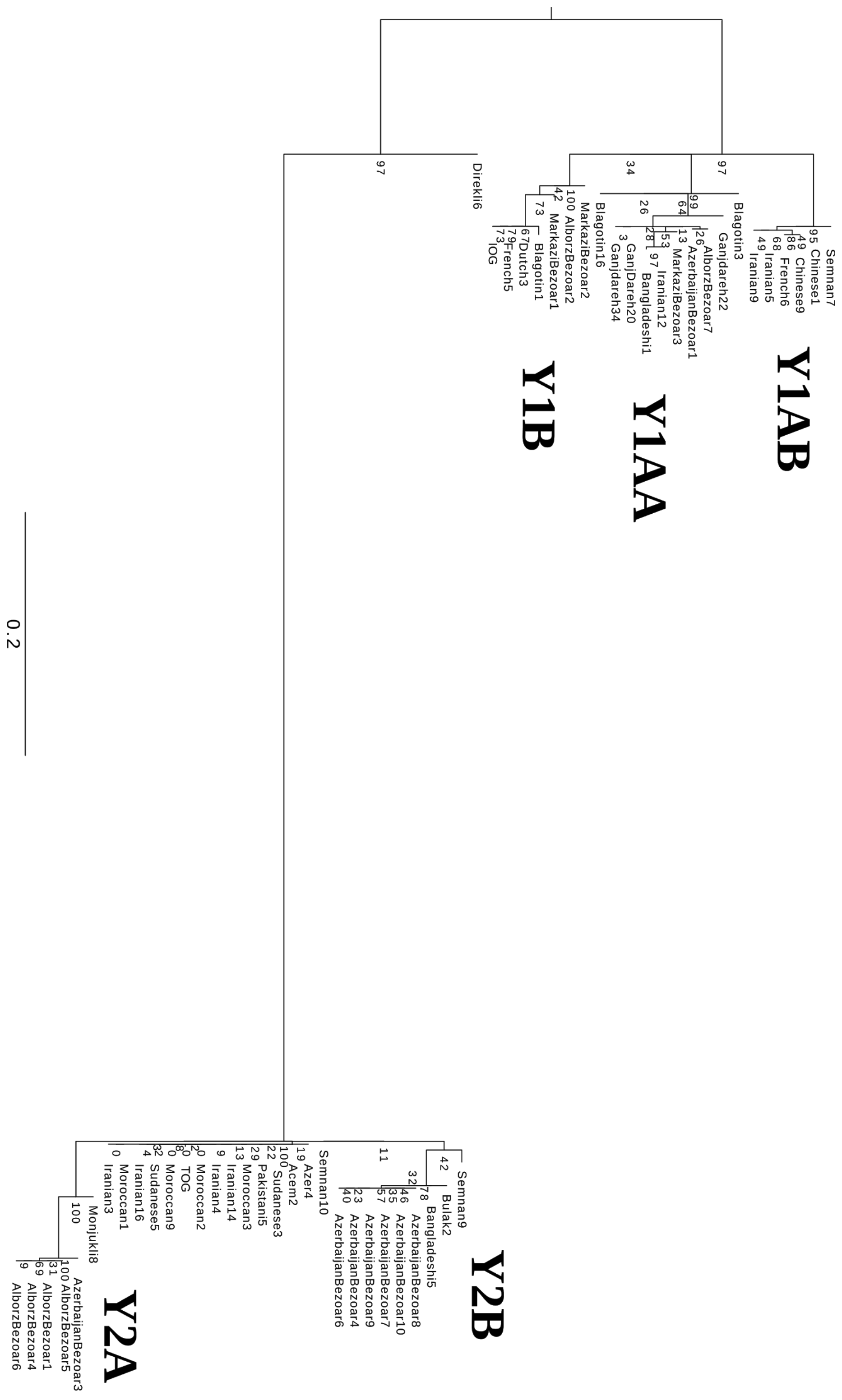

Fig. S30. Maximum Likelihood Y chromosome tree of modern samples and ancient samples <90% missing data, using transversions only. Bootstrap support values for nodes are displayed. Modern haplogroup clades are indicated in bold text, as per (72).

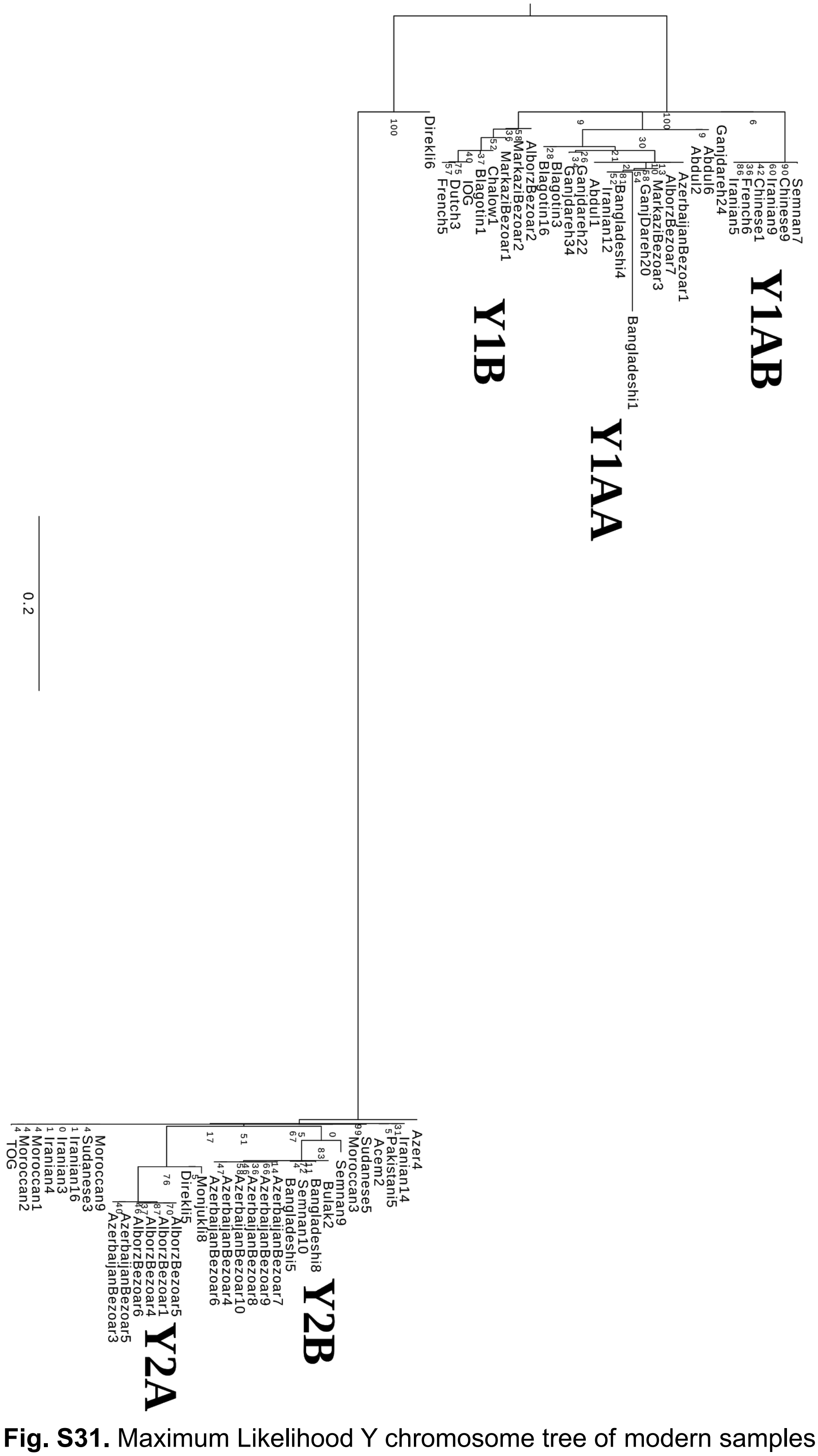

Fig. S31. Maximum Likelihood Y chromosome tree of modern samples and ancient samples <99% missing data, using transitions and transversions. Bootstrap support values for nodes are displayed. Modern haplogroup clades are indicated in bold text, as per (72).

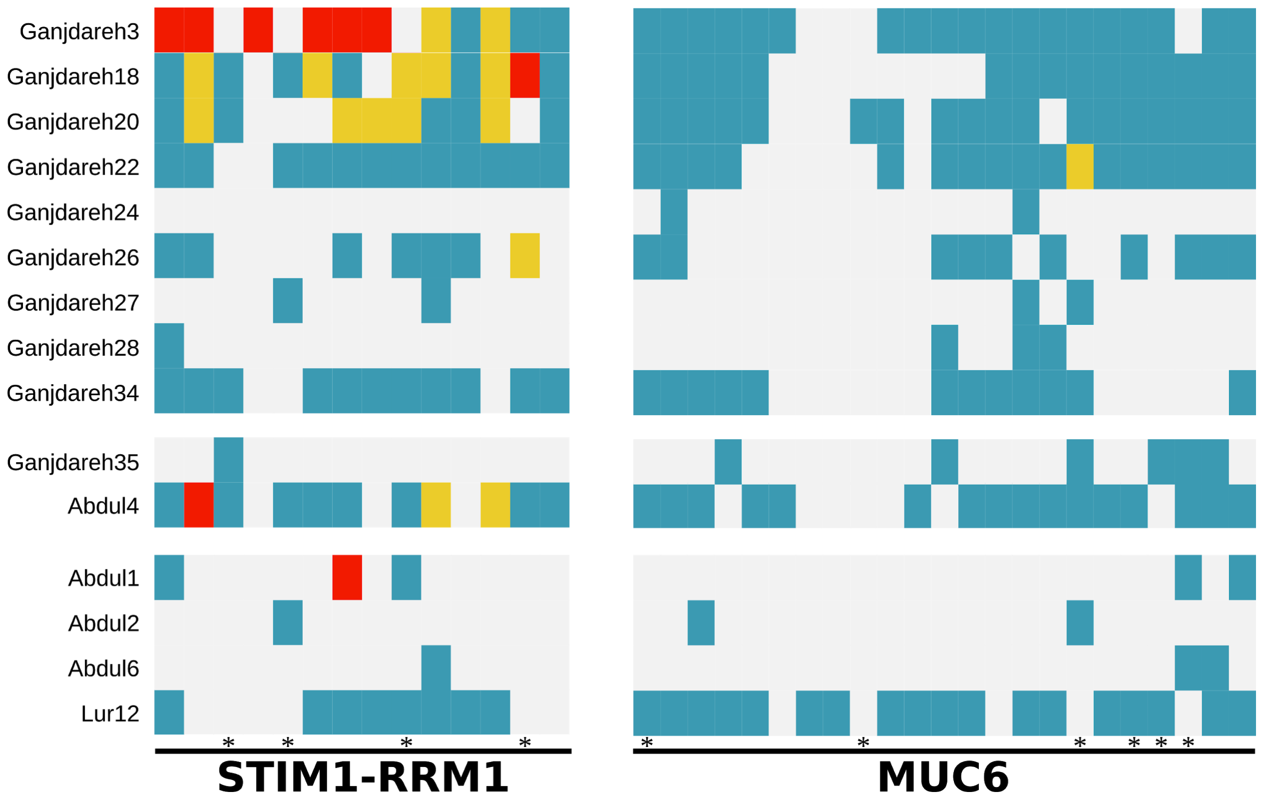

Fig. S32. Genotype of loci identified as being under selection in Zheng (2020). Genotypes are determined by highest likelihood. Asterix indicates ancestral>derived is C>T or G>A. Blue = homozygous ancestry. Yellow = heterozygous. Red = homozygous derived.

Table S1. Site key for Figure 1.

Key	Site(s)	Citation	Key	Site(s)	Citation
1	Hallan Çemi	(79)	13	Karim Shahir	(80)
2	Demirköy	(81)	14	Jarmo	(82)
3	Körtik	(83)	15	Sheikh-e Abad	(84)
4	Hasankeyf	(85)	16	Sarab	(86, 87)
5	Gusir	(88)	17	Guran	(89)
6	Shanidar & Zawi Chemi	(90, 91)	18	Jani	(92)
7	Nemrik	(93)	19	Chogha Golan	(94)
8	Gird Chai	(95)	20	East Chia Sabz	(96, 97)
9	M'lefaat	(98)	21	Kelek Asad Morad	(99)
10	Qermez Dere	(100)	22	Ali Kosh & Tepe Sabz	(101)
11	Bestansur	(102)	23	Chogha Bonut	(103)
12	Matarrah	(104)			

Table S2. Radiocarbon dating for Tepe Abdul and Ganj Dareh goat bone remains. 2 sigma calibration was performed using Oxcal 4.4 (76) and IntCal20 (77).
* reported in (21)

Sample	Uncalibrated Age (BP)	Calibrated C14 Age (95.4% Probability)	C14 Code
Abdul4	8946 ± 47	8275-7958 cal BC	UBA-39204
Abdul6	8759 ± 51	8165-7599 cal BC	UBA-38301
Lur12*	8810 ± 30	8170-7740 cal BC	Beta-470334
Ganjdareh3	8695 ± 47	7940-7592 cal BC	UBA-40250
Ganjdareh18	8839 ± 42	8205-7758 cal BC	UBA-40251
Ganjdareh35	8810 ± 50	8204-7660 cal BC	Poz-124942

Table S3. Caprine harvest profile from multiple sites shown as percentage of total surviving beyond each age class. Rounded to the nearest whole number. Age profiles computed following (9).

Age	Age Class	Asiab Goats	Ganj Dareh Goats - Old Excavations	Ganj Dareh Goats - New Excavations	Ganj Dareh Sheep - Old Excavations	Tepe Abdul Hosein Goats	Ali Kosh Goats
0-6 mo	A	100%	100%	100%	100%	83%	96%
6-12 mo	B	100%	97%	100%	96%	100%	88%
12-18 mo	C	63%	63%	57%	87%	83%	52%
18-30 mo	D	61%	36%	31%	74%	49%	57%
30-48 mo	E	67%	22%	23%	59%	33%	34%
Sample Size NISP		86	2874	162	198	508	1675

Table S4. Sex-specific harvest profiles for goats from multiple sites shown as percentage of total surviving beyond each age class (sample too small from new Ganj Dareh excavations to compute sex-specific harvest profiles). Classification of elements by sex follows (105) that defines ranges of female and male animals for specific dimensions of eight elements. 1st phalanx GL female < 40.98 mm, male > 42.98, 2nd phalanx GL, females < 28.19 mm, males > 30.19 mm; Calcaneus Dp, females < 23.77 mm, males > 25.77 mm; Humerus Dd, females < 28.88 mm, males > 30.88 mm; Metacarpal Bd, females < 30.29 mm, males > 32.29 mm; Metatarsal Bd, females < 26.95 mm, males > 28.95 mm; Radius Bd, females < 30.61 mm, males > 32.61 mm; Tibia Bd, females < 26.86 mm, males > 28.86 mm.

Age	Age Class	Asiab		Ganj Dareh - Old Excavations		Ali Kosh	
		Female	Male	Female	Male	Female	Male
6-12 mo	B	100%	100%	94%	98%	95%	88%
12-18 mo	C	100%	100%	95%	77%	98%	82%
18-30 mo	D	100%	100%	74%	26%	82%	57%
30-48 mo	E	0%	50%	30%	11%	47%	34%
Sample Size NISP		8	12	555	355	380	191

Table S5. Ratios of females to males for goats from multiple sites.

Females & Males	Shanidar Paleolithic	Asiab	Ganj Dareh - Old Excavations	Ganj Dareh - New Excavations	Ali Kosh
Females NISP	13	8	555	31	380
Males NISP	63	12	355	16	191
Total NISP	76	20	910	47	571
Ratio	0.21	0.67	1.56	1.94	1.99

Table S6. Sex-specific and overall harvest profiles for Ganj Dareh goats by level shown as percentage of total surviving beyond each age class (new Ganj Dareh excavations sample too small to compute sex-specific harvest profiles).

Age	Age Class	Level A (%)			Level B (%)			Level C (%)			Level D (%)			Level E (%)		
		Overall	Female	Male	Overall	Female	Male	Overall	Female	Male	Overall	Female	Male	Overall	Female	Male
6-12 mo	B	94.87	90.00	100.00	100.00	92.31	100.00	89.47	75.00	100.00	97.56	100.00	94.44	97.44	100.00	100.00
12-18 mo	C	63.16	100.00	100.00	66.90	93.33	82.86	64.86	100.00	66.67	60.29	91.67	100.00	59.65	92.31	61.11
18-30 mo	D	35.54	76.92	38.71	30.38	73.17	14.81	27.94	60.00	0.00	36.30	78.69	26.47	47.66	70.00	25.00
30-48 mo	E	17.50	35.71	10.71	20.00	31.43	4.76	20.00	17.65	16.67	20.45	28.57	15.79	27.78	24.00	5.26
Sample Size NISP		268	141	85	488	104	94	236	57	30	651	139	76	378	111	67

Table S7. Ratios of females to males for Ganj Dareh goats by level.

Females & Males	Old Excavations					New Excavations		
	Level A	Level B	Level C	Level D	Level E	Levels A-C	Level D	Level E
Females NISP	141	104	57	139	111	23	1	3
Males NISP	85	94	31	76	67	13	1	0
Total NISP	226	198	88	215	178	36	2	3
Ratio	1.66	1.11	1.84	1.83	1.66	1.77	1.00	no males

Table S8. Sample summary for samples reported here, and a Tepe Abdul Hosein sample (Lur12, indicated by *) reported previously (21). Level and Area refers to excavations at Ganj Dareh by Smith (1) and a more recent Iranian/Danish team (106). Ganj Dareh material from the Smithson (Washington DC, United States), Smithsonian NMNH Accession number 1125905, catalog number A587262, are indicated by ☩. Endogenous DNA was calculated by the number of reads passing Q30 and rmdup filters, divided by the number of trimmed reads. Autosomal Coverage was calculated for the 29 *Capra hircus* autosomes after quality filters. Endog. = Endogenous. TP9 = Test Pit 9. EPPN = Early Pre Pottery Neolithic. LPPN = Late Pre-Pottery Neolithic. Pre-Pottery Neolithic is a term traditionally used in reference to the Levant and Upper Mesopotamia region; it is roughly equivalent to the Early or Aceramic Neolithic of the Zagros region, although care must be taken as to how these terms are used across SW Asia, in specific Zagros region, and accounting for region-specific chronologies and mosaic, episodic nature of the development of Neolithic cultures and societies (107).

Sample	Archaeological ID	Level/Area	Bone Element	Sex	Endog. DNA (%)	mtDNA Haplogroup	mtDNA Coverage	Autosomal Coverage
Abdul1	AH78, 10G, 11015, #1	Level 27 /LPPN	Petrous	M	20.49	G'	47.91	0.16
Abdul2	AH78, 10G, 11015, #2.1	Level 27 /LPPN	Petrous	M	24.03	G	86.78	0.24
Abdul4	AH78, 10G, 11009	Level 30/ LPPN	Petrous	F	18.3	G	447.64	2.31
Abdul5	AH78, 10G, 11013	Level 28/ LPPN	Petrous	M	3.09	G	3.36	-
Abdul6	AH78, 18L, 18010	Level 2C/ LPPN	Petrous	M	9.02	A	6.29	0.08
Abdul7	AH78, 10G, 11015	Level 27 /LPPN	Petrous	M	0.78	G	4	-
Abdul9	AH78, 10G, 11051	Level 9/EPPN	Petrous	M	2.62	B	6.84	-
Lur12*	AH78, MM, AH1, 10G, 11064	Level 1 /EPPN	Petrous	F	9.01	G	480	1.01
Ganjdareh1☩	543; A; 18-M/N	A	Petrous	F	0.55	G'	153.05	-
Ganjdareh2☩	565; A; 15-L	A	Petrous	F	0.32	G	40.34	-
Ganjdareh3☩	131; A; 18-N	A	Petrous	F	29.3	G'	1006.27	2.17
Ganjdareh5☩	620; B; 15-Q (N)	B	Petrous	F	4.95	D	234.83	-
Ganjdareh6☩	311; B; 15/16/17P	B	Petrous	F	8.31	D	41.51	-
Ganjdareh7☩	316; B; 15/16P	B	Petrous	C.D.	1.01	G	27.7	-
Ganjdareh8☩	752; C; 19-M/N	C	Petrous	M	2.86	B	139.99	-
Ganjdareh9☩	775; C; 19-M/N	C	Petrous	M	4.26	B	56.29	-
Ganjdareh10☩	748; C; 15-I	C	Petrous	M	5.13	A	124.84	-
Ganjdareh14☩	846; D-TOP; 15-J	D	Petrous	F	11.65	A	66.77	-
Ganjdareh15☩	1425; E; /20-O	E	Petrous	F	7.16	D	40.86	-

Ganjdareh16☩	1536; E; 20-N(b)/20-O	E	Petrous	F	3.68	G'	51.76	-
Ganjdareh18☩	1318; E; 13-G	E	Petrous	F	50.31	A	189.08	2.48
Ganjdareh19☩	1281; E; 14-H	E	Petrous	F	4.69	D	18.61	-
Ganjdareh20☩	1601; E; 15-H	E	Petrous	M	50.54	B	192.34	2.81
Ganjdareh21	GD-2017-A1 1061 A1.5 637	A1.5	Petrous	M	7.27	G'	8.02	-
Ganjdareh22	GD-2018-A1 1067 A1.5 3663	A1.5	Petrous	M	44.75	B	340.37	2.55
Ganjdareh23	GD-2018-D 5055 D.5 2886	D.5	Petrous	M	7.79	D	4.22	-
Ganjdareh24	GD-2018-D 5052 D.5 4395	D.5	Petrous	M	17.13	C bez	49.07	0.10
Ganjdareh26	GD-2018-D 5087 D.6 3345	D.6	Petrous	F	37.19	D bez	150.67	1.20
Ganjdareh27	GD-2018-B 3012 B.4 1565	B.4	Petrous	F	11.97	G	11.83	0.10
Ganjdareh28	GD-2017-A1 1006 A1.1 800	A1.1	Petrous	F	22.27	A	38.81	0.12
Ganjdareh29	GD-2017-TP9 502 4234	TP9	Petrous	M	1.27	C	47.24	-
Ganjdareh34	GD-2018-D 5053 D.2 3186	D.2	Humerus	M	35.81	A	270.08	1.49
Ganjdareh35	GD-2018-D 5076 D.4 3426	D.4	Radius and Ulna	F	16.89	F	8.9	0.16

Table S9. mtDNA contamination estimates. MD=Molecular Damage (transversion variants only).

Sample	Contamination %	Contamination % no MD	Heteroplasmic Contamination %	Heteroplasmic Contamination % no MD
Abdul1	0.24	0.14	1.13	0.65
Abdul2	0.37	0.22	1.59	0.96
Abdul4	0.30	0.22	0.82	0.62
Abdul5	0.00	0.00	NA	NA
Abdul6	0.50	0.25	12.50	6.67
Abdul7	0.00	0.00	NA	NA
Abdul9	0.00	0.00	NA	NA
Ganjdareh1	0.26	0.15	1.23	0.71
Ganjdareh2	0.50	0.31	3.02	1.88
Ganjdareh3	0.17	0.10	0.91	0.55
Ganjdareh5	0.34	0.24	0.89	0.64
Ganjdareh6	0.46	0.23	3.51	1.79
Ganjdareh7	0.54	0.30	3.56	2.01
Ganjdareh8	0.19	0.09	0.83	0.39
Ganjdareh9	0.50	0.36	2.26	1.64
Ganjdareh10	0.35	0.24	1.24	0.88
Ganjdareh14	0.39	0.19	2.41	1.15
Ganjdareh15	0.95	0.36	5.50	2.14
Ganjdareh16	0.39	0.21	2.99	1.63
Ganjdareh18	0.72	0.49	3.57	2.46
Ganjdareh19	1.94	0.89	9.52	4.57
Ganjdareh20	1.15	0.80	4.76	3.37
Ganjdareh21	0.20	0.00	11.11	0.00
Ganjdareh22	0.49	0.31	0.87	0.56
Ganjdareh23	0.00	0.00	NA	NA
Ganjdareh24	0.42	0.16	6.57	2.63
Ganjdareh26	0.80	0.49	1.69	1.05
Ganjdareh27	2.21	1.31	13.18	8.20
Ganjdareh28	0.12	0.08	2.42	1.63
Ganjdareh29	0.30	0.17	1.88	1.05
Ganjdareh34	0.47	0.27	1.04	0.61
Ganjdareh35	0.56	0.19	10.00	3.57

Table S10. Sequencing and alignment statistics for samples reported here. Sequencing information for samples subject to mtDNA enrichment are presented in Table S11.
* reported in (21)

Sample	Platform	Raw Reads	Trimmed Reads	Collapsed Reads (PE)	Aligned Reads	Q30 Rmdup Reads
Abdul1	Hiseq 2500 (1x100bp)	71782023	67748178	-	13876836	10133330
Abdul2	Hiseq 2500 (1x100bp)	82929361	79490074	-	20147508	13507890
Abdul4	Hiseq 2500 (1x100bp)	343267983	318725838	-	183444818	83787233
	NovaSeq (2x100bp)	130146840	12689562	104451405	85326511	53997648
Abdul5	Hiseq 2500 (1x100bp)	4892347	4732487	-	207584	146244
Abdul6	Hiseq 2500 (1x100bp)	60290804	58893520	-	5721193	3877434
Abdul7	Hiseq 2500 (1x100bp)	5390383	5292720	-	57501	41027
Abdul9	Hiseq 2500 (1x100bp)	6101653	5949151	-	229669	155636
Lur12*	Hiseq 2500 (1x100bp)	622799577	606327375	-	-	50672962
Ganjdareh3	Hiseq 2500 (1x100bp)	548658303	524842240	-	209318292	118528257
Ganjdareh18	Hiseq 2500 (1x100bp)	544104619	458051666	-	311664491	158735176
Ganjdareh20	Hiseq 2500 (1x100bp)	527026344	483963877	-	297705304	152575330
Ganjdareh22	NovaSeq (2x100bp)	389418472	82860485	299411013	247424550	122123319
Ganjdareh24	NovaSeq (2x100bp)	42820789	13386975	28430317	8934480	5417390
Ganjdareh26	NovaSeq (2x100bp)	209324438	43765931	159908828	112017680	62442875
Ganjdareh27	NovaSeq (2x100bp)	62511829	22489465	39470080	9912803	4819379
Ganjdareh28	NovaSeq (2x100bp)	49465549	16758796	31421804	10504592	6822656
Ganjdareh34	NovaSeq (2x100bp)	246799476	29588702	210386095	179192520	73229910
Ganjdareh35	NovaSeq (2x100bp)	71648940	17172474	52848044	15207995	7928369

Table S11. mtDNA sequencing and alignment statistics for samples reported here. Raw and filtered read information for samples not subject to enrichment are presented in Table S10.
* reported in (21)

Sample	Raw Reads	Filtered Reads	Q30 Aligned	Coverage	Called Sites	%age Called	mtDNA Haplogroup
Abdul1	-	-	14665	47.91	16625	99.90	G'
Abdul2	-	-	25983	86.78	16641	100	G
Abdul4	-	-	99314	447.64	16641	100	G
Abdul5	-	-	914	3.36	10760	64.66	G
Abdul6	-	-	14343	6.29	15778	94.81	A
Abdul7	-	-	1079	4	12365	74.30	G
Abdul9	-	-	1951	6.84	16158	97.09	B

Lur12*	-	-	127542	480	16641	100	G
Ganjdareh1	5636256	5486782	53674	153.05	16641	100	G'
Ganjdareh2	1867437	1843708	14637	40.34	16633	99.5	G
Ganjdareh3	-	-	300315	1006.27	16641	100	G'
Ganjdareh5	1867437	1843708	74143	234.83	16641	100	D
Ganjdareh6	549880	534735	14716	41.51	16627	99.92	D
Ganjdareh7	2259578	2178906	10557	27.7	16557	99.5	G
Ganjdareh8	1065525	1017703	46138	139.99	16642	100	B
Ganjdareh9	882408	854920	19271	56.29	16642	100	B
Ganjdareh10	970096	947613	40744	124.84	16633	99.5	A
Ganjdareh14	1372814	1330911	24098	66.77	16642	100	A
Ganjdareh15	375457	370069	13798	40.86	16629	99.93	D
Ganjdareh16	244928	240111	18862	51.76	16641	99.92	G'
Ganjdareh18	-	-	75850	189.08	16642	100	A
Ganjdareh19	456145	445345	6880	18.61	16615	99.84	D
Ganjdareh20	-	-	72469	192.34	16642	100	B
Ganjdareh21	40374	37823	2908	8.02	15318	92.05	G'
Ganjdareh22	-	-	105647	340.37	16641	100	B
Ganjdareh23	40766	39815	1475	4.22	11974	71.95	D
Ganjdareh24	-	-	16272	49.07	16575	99.62	C bez
Ganjdareh26	-	-	50146	150.67	16640	99.99	D bez
Ganjdareh27	-	-	4052	11.83	16515	99.24	G
Ganjdareh28	-	-	13177	38.81	16642	100	A
Ganjdareh29	244928	240111	15763	47.24	16613	99.85	C
Ganjdareh34	-	-	80031	270.08	16642	100	A
Ganjdareh35	-	-	3008	8.9	16639	100	F

Table S12. Ancient sample details and analysis groupings. Cov. = X-fold coverage. NG = Nuclear Genome.

Sample	Site	Region	Sex	Context	NG Grouping	mtDNA AMOVA Groupings	Cov.	mtDNA
Abdul1	Tepe Abdul Hosein	Luristan, Iran	M	Aceramic Neolithic	AN Zagros Main	Abdul Hosein; Zagros	0.16	G'
Abdul2	Tepe Abdul Hosein	Luristan, Iran	M	Aceramic Neolithic	AN Zagros Main	Abdul Hosein; Zagros	0.24	G
Abdul4	Tepe Abdul Hosein	Luristan, Iran	F	Aceramic Neolithic	AN Zagros Outlier	Abdul Hosein; Zagros	2.31	G
Abdul5	Tepe Abdul Hosein	Luristan, Iran	M	Aceramic Neolithic	-	-	-	G
Abdul6	Tepe Abdul Hosein	Luristan, Iran	M	Aceramic Neolithic	AN Zagros Main	Abdul Hosein; Zagros	0.08	A
Abdul7	Tepe Abdul Hosein	Luristan, Iran	M	Aceramic Neolithic	-	-	-	G
Abdul9	Tepe Abdul Hosein	Luristan, Iran	M	Aceramic Neolithic	-	Abdul Hosein; Zagros	-	B
Acem1	Acemhöyük	Aksaray Plain, Turkey	F	Bronze Age	Bronze Age Turkey		4.35	A
Acem2	Acemhöyük	Aksaray Plain, Turkey	M	Bronze Age	Bronze Age Turkey	-	8.63	A
Ainghazal1	'Ain Ghazal	Amman, Jordan	M	Aceramic Neolithic	PPN Levant	Levant; Levant	0.03	F
Ainghazal2	'Ain Ghazal	Amman, Jordan	F	Aceramic Neolithic	PPN Levant	Levant; Levant	0.06	F
Ainghazal3	'Ain Ghazal	Amman, Jordan	F	Aceramic Neolithic	PPN Levant	-	0.002	F
Ainghazal4	'Ain Ghazal	Amman, Jordan	M	Aceramic Neolithic	PPN Levant	Levant; Levant	0.01	F
AP38	Aşağı Pınar	Kırklareli, Turkey	F	Ceramic Neolithic	-	West Turkey; Europe	-	C
AP44	Aşağı Pınar	Kırklareli, Turkey	F	Ceramic Neolithic	-	-	-	A
AP45	Aşağı Pınar	Kırklareli, Turkey	F	Ceramic Neolithic	Neolithic SE Europe	West Turkey; Europe	0.02	A
AP46	Aşağı Pınar	Kırklareli, Turkey	F	Ceramic Neolithic	-	West Turkey; Europe	-	C
AP49	Aşağı Pınar	Kırklareli, Turkey	F	Ceramic Neolithic	Neolithic SE Europe	West Turkey; Europe	0.02	A
AP50	Aşağı Pınar	Kırklareli, Turkey	F	Ceramic Neolithic	-	West Turkey; Europe	-	A
Azer3-5	Tepe Hasanlu	Azerbaijan, Iran	F	Bronze Age	Bronze Age Caucasus	-	4.54	A
Azer4	Tepe Hasanlu	Azerbaijan, Iran	M	Iron Age	Iron Age/Medieval Caucasus	-	2.56	A
Azer6	Soha Chay Tepe	Azerbaijan, Iran	F	Chalcolithic	Chalcolithic Caucasus	-	0.28	A
Blagotin1	Blagotin-Poljna	Trstenik, Serbia	M	Ceramic Neolithic	Neolithic Serbia	Serbia; Europe	6.93	A
Blagotin16	Blagotin-Poljna	Trstenik, Serbia	M	Ceramic Neolithic	Neolithic Serbia	Serbia; Europe	3.48	A
Blagotin2	Blagotin-Poljna	Trstenik, Serbia	F	Ceramic Neolithic	Neolithic Serbia	Serbia; Europe	3.90	A
Blagotin3	Blagotin-Poljna	Trstenik, Serbia	M	Ceramic Neolithic	Neolithic Serbia	Serbia; Europe	11.40	A
Bulak1	Tilla Bulak	Surkhandarja, Uzbekistan	F	Bronze Age	Bronze Age Uzbekistan	-	0.85	A
Bulak2	Tilla Bulak	Surkhandarja, Uzbekistan	M	Bronze Age	Bronze Age Uzbekistan	-	2.66	A
Bulak4	Tilla Bulak	Surkhandarja, Uzbekistan	M	Bronze Age	-	-	-	B
Bulak5	Tilla Bulak	Surkhandarja, Uzbekistan	F	Bronze Age	Bronze Age Uzbekistan	-	0.26	D
Cav8	Čavdar	Sofia District, Bulgaria	F	Ceramic Neolithic	-	-	-	A
Chalow1	Chalow	Khorasan, Iran	M	Bronze Age	Bronze Age Iran	-	0.05	D

Darre1	Darre-ye Bolāghi	Fars, Iran	F	Chalcolithic	Chalcolithic Iran	-	0.04	A
Darre2	Darre-ye Bolāghi	Fars, Iran	F	Medieval	Iron Age/Medieval Iran	-	3.83	A
Direkli1-2	Direkli Cave	Taurus Mountains, Turkey	F	Late Epipaleolithic	Epipaleolithic Taurus	-	11.21	T
Direkli4	Direkli Cave	Taurus Mountains, Turkey	M	Late Epipaleolithic	-	-	-	F
Direkli5	Direkli Cave	Taurus Mountains, Turkey	M	Late Epipaleolithic	Epipaleolithic Taurus	-	0.26	T
Direkli6	Direkli Cave	Taurus Mountains, Turkey	M	Late Epipaleolithic	Epipaleolithic Taurus	-	2.50	T
Dra34	Merdžumekja	Drama, Bulgaria	F	Chalcolithic	-	-	-	G
Fars1	Rahmat Abad	Fars, Iran	M	Chalcolithic	Chalcolithic Iran	-	0.02	A
Fars2-5	Rahmat Abad	Fars, Iran	M	Ceramic Neolithic	Ceramic Neolithic Zagros	-	0.03	B
Fars4	Mianroud	Fars, Iran	F	Chalcolithic	Chalcolithic Iran	-	1.01	A
GanjDareh1	Ganj Dareh	Iran	F	Aceramic Neolithic	-	Ganj Dareh; Zagros	-	G'
GanjDareh2	Ganj Dareh	Iran	F	Aceramic Neolithic	-	Ganj Dareh; Zagros	-	G
GanjDareh3	Ganj Dareh	Iran	F	Aceramic Neolithic	AN Zagros Main	Ganj Dareh; Zagros	2.17	G'
GanjDareh5	Ganj Dareh	Iran	F	Aceramic Neolithic	-	Ganj Dareh; Zagros	-	D
GanjDareh6	Ganj Dareh	Iran	F	Aceramic Neolithic	-	Ganj Dareh; Zagros	-	D
GanjDareh7	Ganj Dareh	Iran	?	Aceramic Neolithic	-	Ganj Dareh; Zagros	-	G
GanjDareh8	Ganj Dareh	Iran	M	Aceramic Neolithic	-	Ganj Dareh; Zagros	-	B
GanjDareh9	Ganj Dareh	Iran	M	Aceramic Neolithic	-	Ganj Dareh; Zagros	-	B
GanjDareh10	Ganj Dareh	Iran	M	Aceramic Neolithic	-	Ganj Dareh; Zagros	-	A
GanjDareh14	Ganj Dareh	Iran	F	Aceramic Neolithic	-	Ganj Dareh; Zagros	-	A
GanjDareh15	Ganj Dareh	Iran	F	Aceramic Neolithic	-	Ganj Dareh; Zagros	-	D
GanjDareh16	Ganj Dareh	Iran	F	Aceramic Neolithic	-	Ganj Dareh; Zagros	-	G'
GanjDareh18	Ganj Dareh	Iran	F	Aceramic Neolithic	AN Zagros Main	Ganj Dareh; Zagros	2.48	A
GanjDareh19	Ganj Dareh	Iran	F	Aceramic Neolithic	AN Zagros Main	Ganj Dareh; Zagros	-	D
GanjDareh20	Ganj Dareh	Iran	M	Aceramic Neolithic	AN Zagros Main	Ganj Dareh; Zagros	2.81	B
Ganjdareh21	Ganj Dareh	Iran	M	Aceramic Neolithic	AN Zagros Main	Ganj Dareh; Zagros	-	G'
Ganjdareh22	Ganj Dareh	Iran	M	Aceramic Neolithic	AN Zagros Main	Ganj Dareh; Zagros	2.55	B
Ganjdareh23	Ganj Dareh	Iran	M	Aceramic Neolithic	-	-	-	D
Ganjdareh24	Ganj Dareh	Iran	M	Aceramic Neolithic	AN Zagros Main	Ganj Dareh; Zagros	0.1	C bez
Ganjdareh26	Ganj Dareh	Iran	F	Aceramic Neolithic	AN Zagros Main	Ganj Dareh; Zagros	1.2	D bez
Ganjdareh27	Ganj Dareh	Iran	F	Aceramic Neolithic	AN Zagros Main	Ganj Dareh; Zagros	0.1	G
Ganjdareh28	Ganj Dareh	Iran	F	Aceramic Neolithic	AN Zagros Main	Ganj Dareh; Zagros	0.12	A
Ganjdareh29	Ganj Dareh	Iran	M	Aceramic Neolithic	-	Ganj Dareh; Zagros	-	C
Ganjdareh34	Ganj Dareh	Iran	M	Aceramic Neolithic	AN Zagros Main	Ganj Dareh; Zagros	1.49	A
Ganjdareh35	Ganj Dareh	Iran	F	Aceramic Neolithic	AN Zagros Outlier	Ganj Dareh; Zagros	0.16	F
Geor2	Tamara Fort	Kazbegi, Georgia	M	Medieval	Iron Age/Medieval Iran	-	1.5	A
Ghosh5	Abu Ghosh	Judean Hills, Israel	F	Aceramic Neolithic	PPN Levant	Levant; Levant	0.001	F
Gilat2	Gilat	Northern Negev, Israel	M	Chalcolithic	-	-	0.001	A

Gilat8	Gilat	Northern Negev, Israel	M	Chalcolithic	Chalcolithic Levant	-	0.02	D
Gilat10	Gilat	Northern Negev, Israel	F	Chalcolithic	-	-	0.001	A
Hovk1	Hovk-1 Cave	Tavush, Armenia	F	Late Pleistocene	Pleistocene Armenia	-	3.21	F
Kan19	Kanlıgeçit	Kırklareli, Turkey	C.D.	Bronze Age	-	-	-	A
Kan23	Kanlıgeçit	Kırklareli, Turkey	M	Bronze Age	-	-	-	G'
Kan25	Kanlıgeçit	Kırklareli, Turkey	C.D.	Bronze Age	-	-	-	A
Kazbeg1	Tamara Fort	Kazbegi, Georgia	F	Medieval	Iron Age/Medieval Georgia	-	3.74	A
Kohneh2	Kohneh Tepesi	Azerbaijan, Iran	F	Bronze Age	Bronze Age Caucasus	-	0.04	A
Kov27	Kovačevo	Blagoevgrad, Bulgaria	C.D.	Ceramic Neolithic	-	-	-	A
Kov57	Kovačevo	Blagoevgrad, Bulgaria	F	Ceramic Neolithic	Neolithic SE Europe	Bulgaria; Europe	0.07	A
Kov60	Kovačevo	Blagoevgrad, Bulgaria	F	Ceramic Neolithic	-	Bulgaria; Europe	-	A
Lur9	Kelek Asad Morad	Luristan, Iran	F	Aceramic Neolithic	-		-	B
Lur12	Tepe Abdul Hosein	Luristan, Iran	F	Aceramic Neolithic	AN Zagros Main	Abdul Hosein; Zagros	1.01	G
Miqne5	Tel Miqne-Ekron	Shephelah, Israel	M	Iron Age	Iron Age/Medieval Levant	-	0.001	A
Monjukli1	Monjukli Depe	Meana-Čaača, Turkmenistan	F	Eneolithic/Chalcolithic	Chalcolithic Turkmenistan	-	0.23	A
Monjukli2	Monjukli Depe	Meana-Čaača, Turkmenistan	F	Eneolithic/Chalcolithic	Chalcolithic Turkmenistan	-	0.21	D
Monjukli4	Monjukli Depe	Meana-Čaača, Turkmenistan	F	Eneolithic/Chalcolithic	Chalcolithic Turkmenistan	-	0.58	A
Monjukli6	Monjukli Depe	Meana-Čaača, Turkmenistan	M	Eneolithic/Chalcolithic	Chalcolithic Turkmenistan	-	0.03	D
Monjukli7	Monjukli Depe	Meana-Čaača, Turkmenistan	F	Ceramic Neolithic	-	Turkmenistan; East	-	D
Monjukli8	Monjukli Depe	Meana-Čaača, Turkmenistan	M	Ceramic Neolithic	Neolithic East Iran/Turkmenistan	Turkmenistan; East	2.55	D
Monjukli9	Monjukli Depe	Meana-Čaača, Turkmenistan	M	Ceramic Neolithic	-	Turkmenistan; East	-	G'
Ovc11	Ovčarovo-gorata	Tărgovište, Bulgaria	F	Ceramic Neolithic	-	Bulgaria; Europe	-	A
Pie17	Pietrele	Giurgiu, Romania	F	Chalcolithic	-	-	-	A
Potterne1	Potterne	Wiltshire, UK	F	Bronze Age	Bronze Age Britain	-	3.57	A
Qazvin1	Tepe Chizar	Qazvin, Iran	F	Bronze Age	Bronze Age Iran	-	3.13	A
Safi2	Tel es-Safi	Ashkelon, Israel	F	Bronze Age	Bronze Age Levant	-	0.04	A
Semnan1-2	Sang-e Chakhmaq	Semnan, Iran	F	Aceramic Neolithic	Neolithic East Iran/Turkmenistan	East Iran; East	6.61	B
Semnan3	Sang-e Chakhmaq	Semnan, Iran	F	Ceramic Neolithic	Neolithic East Iran/Turkmenistan	East Iran; East	14.44	D
Semnan7	Sang-e Chakhmaq	Semnan, Iran	M	Ceramic Neolithic	Neolithic East Iran/Turkmenistan	East Iran; East	3.26	D
Semnan8	Sang-e Chakhmaq	Semnan, Iran	F	Ceramic Neolithic	Neolithic East Iran/Turkmenistan	East Iran; East	0.2	D

Semnan9	Sang-e Chakhmaq	Semnan, Iran	M	Ceramic Neolithic	Neolithic East Iran/Turkmenistan	East Iran; East	3.04	G'
Semnan10	Sang-e Chakhmaq	Semnan, Iran	M	Ceramic Neolithic	Neolithic East Iran/Turkmenistan	East Iran; East	1.42	G'
Semnan13	Sang-e Chakhmaq	Semnan, Iran	F	Ceramic Neolithic	Neolithic East Iran/Turkmenistan	East Iran; East	2.83	D
Semnan17	Sang-e Chakhmaq	Semnan, Iran	F	Ceramic Neolithic	Neolithic East Iran/Turkmenistan	East Iran; East	0.12	D
Shiqmim1	Shiqmim	Northern Negev, Israel	F	Chalcolithic	Chalcolithic Levant	-	0.001	D
Shiqmim9	Shiqmim	Northern Negev, Israel	M	Chalcolithic	Chalcolithic Levant	-	0.001	D
Tac1	Tachti Perda	Kakheti, Georgia	F	Bronze Age	-	-	-	A
Tac2	Tachti Perda	Kakheti, Georgia	F	Iron Age	-	-	-	A
Tac3	Tachti Perda	Kakheti, Georgia	F	Bronze Age	Bronze Age Caucasus	-	0.13	A
Uiv17	Uivar	Timişoara, Romania	F	Aceramic Neolithic	-	-	-	A
Ulu38	Ulucak Höyük	Turkey	C.D.	Ceramic Neolithic	-	-	-	A
Yarmut1	Tel Yarmuth	Bet Shemesh, Israel	M	Bronze Age	Bronze Age Levant	-	0.001	A
Yarmut7	Tel Yarmuth	Bet Shemesh, Israel	F	Bronze Age	Bronze Age Levant	-	0.01	A
Yoqneam2	Tel Yoqne'am	Haifa, Israel	F	Bronze Age	Bronze Age Levant	-	2.13	A

Table S13. Modern goat samples used in modern call set or subsampled for ancestry analysis. Groupings used correspond to sample name i.e. French1, French2 etc placed in French group for analysis.

Sample	Sample Accession	Sex	Coverage	Subsample	Call Set	Sample	Sample Accession	Sex	Coverage	Subsample	Call Set
Bangladeshi1	SAMN10391846	M	37.89	Y	Y	French3	SAMEA2417034	F	13.34	Y	Y
Bangladeshi2	SAMN10460900	F	10.55		Y	French4	SAMEA2417036	F	14.06	Y	Y
Bangladeshi3	SAMN10460904	F	10.65		Y	French5	SAMN07156522	M	11.3		Y
Bangladeshi4	SAMN10460883	M	10.89		Y	French6	SAMN07156523	M	14.6		Y
Bangladeshi5	SAMN10460890	M	10.92	Y	Y	Italian1	SAMEA2297390	F	15.37		Y
Bangladeshi6	SAMN07156543	F	9.39			Italian2	SAMEA2297391	F	15.21		Y
Bangladeshi7	SAMN07156544	F	9.2			Italian3	SAMEA2297392	F	14.91		Y
Bangladeshi8	SAMN07156545	M	9.91	Y		Italian4	SAMEA2297392	F	14.37	Y	Y
Pakistani1	SAMN07156546	F	13.62		Y	Italian5	SAMEA2297394	F	14.19		Y
Pakistani2	SAMN07156547	F	11.74		Y	Irish Old Goat	SAMEA4609295	M	42.75		Y
Pakistani3	SAMN07156548	F	13.27		Y	Spanish1	SAMN07156520	F	13.61	Y	
Pakistani4	SAMN07156549	F	12.54		Y	Spanish2	SAMN07156521	F	10.43	Y	Y
Pakistani5	SAMN07156550	M	12.59		Y	Dutch1	SAMN07156524	M	13.27	Y	
Moroccan1	SAMEA2012964	M	12.94	Y	Y	Dutch2	SAMN07156525	F	13.95		Y
Moroccan2	SAMEA2012707	M	14.12	Y	Y	Dutch3	SAMN07156526	M	11.19	Y	Y

Moroccan3	SAMEA2012705	M	13.82		Y	Swiss1	SAMN04453658	F	16.04	Y	Y
Moroccan4	SAMEA2012826	F	15.29		Y	Swiss2	SAMN04453659	F	11.16	Y	Y
Moroccan5	SAMEA2012908	F	12.54	Y	Y	Iranian1	SAMEA2065423	F	13.82	Y	Y
Moroccan6	SAMEA2013048	F	14.97		Y	Iranian2	SAMEA2065431	F	11.97	Y	Y
Moroccan7	SAMEA2013062	F	15.74		Y	Iranian3	SAMEA2065424	M	13.15		Y
Moroccan8	SAMEA2012822	F	13.42			Iranian4	SAMEA2065422	M	13.37		Y
Moroccan9	SAMEA2012903	M	13.18	Y	Y	Iranian5	SAMEA2065426	M	11.85	Y	
Moroccan18	SAMEA2037792	F	10.77			Iranian6	SAMEA1968884	F	12.98		
Togolese	SAMEA4609296	M	38.7	Y	Y	Iranian7	SAMEA2065433	F	13.35		Y
Ethiopian1	SAMN07156478	F	11.16	Y	Y	Iranian8	SAMEA2065438	F	11.47	Y	Y
Ethiopian2	SAMN07156479	F	9.68			Iranian9	SAMEA2065437	M	11.49		Y
Nigerian1	SAMN07156480	F	8.05			Iranian10	SAMEA2065436	F	12.92		Y
Nigerian2	SAMN07156481	F	11.14			Iranian11	SAMEA2065435	F	13.15	Y	Y
Sudanese1	SAMN10995949	F	9.57	Y	Y	Iranian12	SAMEA2065587	M	12.46	Y	Y
Sudanese2	SAMN10995951	F	10.35	Y	Y	Iranian13	SAMEA2065428	F	13.15		Y
Sudanese3	SAMN10995952	M	8.96			Iranian14	SAMEA2065432	M	12.46		
Sudanese4	SAMN10995950	F	9.73			Iranian15	SAMEA2065425	F	12.27	Y	Y
Sudanese5	SAMN10995953	M	9.75			Iranian16	SAMEA2065429	M	13.97		Y
Sudanese6	SAMN10995954	F	9.47	Y		Iranian17	SAMEA2065430	F	12.72		Y
Chinese_CHIR1	SAMN02953816	F	36.65		Y	Iranian18	SAMEA2065434	F	12.85	Y	
Chinese_Alashan1	SAMN05592064	F	6.38			Iranian19	SAMEA2065427	F	13.58		
Chinese_Alashan2	SAMN05592070	F	1.77			Iranian20	SAMEA1966659	F	13.21	Y	Y
Chinese_Alashan3	SAMN05592075	F	3.34			Iranian21	SAMN07156527	F	12.99		
Chinese_Alashan4	SAMN05592080	F	1.79			Iranian22	SAMN07156531	F	11.87		Y
Chinese_Alashan5	SAMN05592082	F	6.24	Y		Iranian23	SAMN07156534	F	16.13	Y	Y
Chinese_Liaoning1	SAMN05592082	F	4.46			Azerbaijan_Bezoar1	SAMEA2065220	M	12.56	Y	Y
Chinese_Liaoning2	SAMN05601117	F	2.03			Azerbaijan_Bezoar3	SAMEA2065212	M	12.61	Y	Y
Chinese_Liaoning3	SAMN05601123	F	1.85			Azerbaijan_Bezoar4	SAMEA2065216	M	6.89	Y	
Chinese_Liaoning4	SAMN05601126	F	3.98			Azerbaijan_Bezoar5	SAMEA2065217	M	6.76	Y	
Chinese_Liaoning5	SAMN05601128	F	1.15			Azerbaijan_Bezoar6	SAMEA2065214	M	7.65	Y	
Chinese_Aerbasi1	SAMN05601591	F	1.64	Y		Azerbaijan_Bezoar7	SAMEA2065213	M	11.65	Y	Y
Chinese_Aerbasi2	SAMN05601592	F	4.55			Azerbaijan_Bezoar8	SAMEA2065215	M	12.3	Y	Y
Chinese_Aerbasi3	SAMN05601602	F	1.42			Azerbaijan_Bezoar9	SAMEA2065218	M	13.13	Y	Y
Chinese_Aerbasi4	SAMN05601605	F	6.56			Azerbaijan_Bezoar10	SAMEA2188056	M	12.86	Y	Y
Chinese_Aerbasi5	SAMN05601608	F	0.28			Zagros_Bezoar1	SAMEA2065222	M	5.72	Y	
Chinese_Aerbasi6	SAMN05601625	F	2.3			Zagros_Bezoar2	SAMEA2065221	M	6.62	Y	
Chinese_Erlangshan1	SAMN05601632	F	1.65			Zagros_Bezoar3	SAMEA2065225	M	6.91	Y	
Chinese_Erlangshan2	SAMN05601633	F	4.8			Zagros_Bezoar4	SAMEA2065223	M	5.38	Y	
Chinese_Erlangshan3	SAMN05601636	F	3.55			Markazi_Bezoar1	SAMEA2065227	M	10.96	Y	Y

Chinese_Erlangshan4	SAMN05601638	F	1.41			Markazi_Bezoar2	SAMN03071857	M	19.58	Y	Y
Chinese1	SAMN07156559	M	10.34	Y		Markazi_Bezoar3	SAMN07156477	M	11.9	Y	Y
Chinese2	SAMN07156552	F	12.01		Y	Alborz_Bezoar1	SAMEA2395407	M	15.09	Y	
Chinese3	SAMN07156554	F	11.32			Alborz_Bezoar2	SAMEA1966535	M	12.95	Y	Y
Chinese4	SAMN07156514	F	19.18	Y	Y	Alborz_Bezoar3	SAMEA2065421	F	6.82	Y	
Chinese5	SAMN07156515	F	26.31		Y	Alborz_Bezoar4	SAMEA2065226	M	11.86	Y	Y
Chinese6	SAMN07156519	F	18.17			Alborz_Bezoar5	SAMEA2395406	M	14.88	Y	Y
Chinese7	SAMN07156492	F	11.72	Y	Y	Alborz_Bezoar6	SAMEA2395408	M	14.74	Y	
Chinese8	SAMN07156494	F	9.43			Alborz_Bezoar7	SAMEA2065224	M	12.36	Y	Y
Chinese9	SAMN07156500	M	10.5		Y	Tur1	SAMN10995957	M	2.81		
French1	SAMEA2417035	F	14.41		Y	Sheep	SAMN02720826	F	29.63		Y

Table S14. qpAdm results. The *Zagros Outlier* group was the target in all tests. AzB = Azerbaijan Bezoar; AlB = Alborz Bezoar; ZaB = Zagros Bezoar; MaB = Markazi Bezoar; PAr = Pleistocene Armenia; ETa = Epipaleolithic Taurus; AN_Zag = *Zagros Main* group.

Sources	Reference Populations	p value	Mixture Proportions	SE	Accept
AzB	AlB,ZaB,MaB,PAr,ETa,AN_Zag,Sheep	1.96E-105	0	0	No
AlB	AzB,ZaB,MaB,PAr,ETa,AN_Zag,Sheep	2.84E-65	0	0	No
ZaB	AzB,AlB,MaB,PAr,ETa,AN_Zag,Sheep	0.581871905	0	0	Yes
MaB	AzB,AlB,ZaB,PAr,ETa,AN_Zag,Sheep	6.16E-10	0	0	No
PAr	AzB,AlB,ZaB,MaB,ETa,AN_Zag,Sheep	1.05E-63	0	0	No
ETa	AzB,AlB,ZaB,MaB,PAr,AN_Zag,Sheep	3.23E-38	0	0	No
AN_Zag	AzB,AlB,ZaB,MaB,PAr,ETa,Sheep	5.32E-08	0	0	No
Sheep	AzB,AlB,ZaB,MaB,PAr,ETa,AN_Zag	2.66E-07	0	0	No
AlB,PAr	AzB,ZaB,MaB,ETa,AN_Zag,Sheep	4.12E-08	4.591,-3.591	1.167,1.167	No
AlB,ETa	AzB,ZaB,MaB,PAr,AN_Zag,Sheep	3.73E-07	-5.393,6.393	2.443,2.443	No
PAr,Sheep	AzB,AlB,ZaB,MaB,ETa,AN_Zag	3.66E-07	-0.083,1.083	0.082,0.082	No
AzB,Sheep	AlB,ZaB,MaB,PAr,ETa,AN_Zag	3.21E-05	-0.261,1.261	0.099,0.099	No
AzB,ETa	AlB,ZaB,MaB,PAr,AN_Zag,Sheep	1.77E-06	-1.133,2.133	0.189,0.189	No
AlB,Sheep	AzB,ZaB,MaB,PAr,ETa,AN_Zag	1.56E-07	-0.041,1.041	0.091,0.091	No
AzB,PAr	AlB,ZaB,MaB,ETa,AN_Zag,Sheep	1.48E-07	-4.056,5.056	1.362,1.362	No
ETa,Sheep	AzB,AlB,ZaB,MaB,PAr,AN_Zag	1.12E-05	-0.070,1.070	0.121,0.121	No
ZaB,AN_Zag	AzB,AlB,MaB,PAr,ETa,Sheep	0.955599319	1.025,-0.025	0.195,0.195	No
ZaB,MaB	AzB,AlB,PAr,ETa,AN_Zag,Sheep	0.915252168	0.801,0.199	0.120,0.120	No
PAr,ETa	AzB,AlB,ZaB,MaB,AN_Zag,Sheep	0.837393601	-2.376,3.376	0.529,0.529	No
ETa,AN_Zag	AzB,AlB,ZaB,MaB,PAr,Sheep	0.798387064	0.292,0.708	0.045,0.045	Yes
ZaB,Sheep	AzB,AlB,MaB,PAr,ETa,AN_Zag	0.77534288	1.286,-0.286	0.204,0.204	No

AlB,AN_Zag	AzB,ZaB,MaB,PAr,ETa,Sheep	0.70342337	0.267,0.733	0.040,0.040	Yes
AzB,AN_Zag	AlB,ZaB,MaB,PAr,ETa,Sheep	0.57459851	0.180,0.820	0.034,0.034	Yes
AlB,MaB	AzB,ZaB,PAr,ETa,AN_Zag,Sheep	0.450621014	0.293,0.707	0.040,0.040	Yes
MaB,ETa	AzB,AlB,ZaB,PAr,AN_Zag,Sheep	0.445096471	0.675,0.325	0.045,0.045	Yes
AzB,ZaB	AlB,MaB,PAr,ETa,AN_Zag,Sheep	0.413952251	-0.043,1.043	0.051,0.051	No
AlB,ZaB	AzB,MaB,PAr,ETa,AN_Zag,Sheep	0.40940038	-0.053,1.053	0.069,0.069	No
ZaB,ETa	AzB,AlB,MaB,PAr,AN_Zag,Sheep	0.400895503	1.041,-0.041	0.078,0.078	No
ZaB,PAr	AzB,AlB,MaB,ETa,AN_Zag,Sheep	0.38692581	1.032,-0.032	0.051,0.051	No
PAr,AN_Zag	AzB,AlB,ZaB,MaB,ETa,Sheep	0.377413524	0.210,0.790	0.033,0.033	Yes
AzB,MaB	AlB,ZaB,PAr,ETa,AN_Zag,Sheep	0.3642738	0.198,0.802	0.038,0.038	Yes
MaB,PAr	AzB,AlB,ZaB,ETa,AN_Zag,Sheep	0.244606261	0.756,0.244	0.037,0.037	Yes
MaB,AN_Zag	AzB,AlB,ZaB,PAr,ETa,Sheep	0.10487526	-0.637,1.637	0.322,0.322	No
AzB,AlB	ZaB,MaB,PAr,ETa,AN_Zag,Sheep	0.00473750758	-1.460,2.460	0.231,0.231	No
MaB,Sheep	AzB,AlB,ZaB,PAr,ETa,AN_Zag	0.00297543092	0.504,0.496	0.100,0.100	No
AN_Zag,Sheep	AzB,AlB,ZaB,MaB,PAr,ETa	0.00153698473	0.329,0.671	0.195,0.195	No
AlB	MaB,ZaB,AzB,Sheep	1.05E-54	0	0	No
MaB	AlB,ZaB,AzB,Sheep	2.70E-07	0	0	No
ZaB	AlB,MaB,AzB,Sheep	0.900968445	0	0	Yes
AzB	AlB,MaB,ZaB,Sheep	1.42E-78	0	0	No
Sheep	AlB,MaB,ZaB,AzB	0.0908015199	0	0	Yes
AlB,MaB	ZaB,AzB,Sheep	0.416705992	0.320,0.680	0.053,0.053	Yes
AlB,ZaB	MaB,AzB,Sheep	0.478446072	0.013,0.987	0.073,0.073	No
AlB,AzB	MaB,ZaB,Sheep	0.0375209327	2.269,-1.269	0.818,0.818	No
AlB,Sheep	MaB,ZaB,AzB	0.135418005	0.047,0.953	0.076,0.076	No
MaB,ZaB	AlB,AzB,Sheep	0.561964456	0.095,0.905	0.228,0.228	No
MaB,AzB	AlB,ZaB,Sheep	0.637253896	0.811,0.189	0.060,0.060	Yes
MaB,Sheep	AlB,ZaB,AzB	0.016295808	0.125,0.875	0.265,0.265	No
ZaB,AzB	AlB,MaB,Sheep	0.453631542	1.002,-0.002	0.056,0.056	No
ZaB,Sheep	AlB,MaB,AzB	0.835304438	1.060,-0.060	0.471,0.471	No
AzB,Sheep	AlB,MaB,ZaB	0.0502306517	-0.071,1.071	0.083,0.083	No

Table S15. qpWave results. "Left" populations were *Zagros Main* and Neolithic Iranian/Turkmenistan groups. Columns indicate "Right" population sets and are described in the "qpWave" section. Cell entries are p values for models, rounded to three decimal points; values <0.05 represent rejection of the current model of some number of waves of gene flow from "Right" populations explaining the ancestry of "Left" populations.

	**Wilds+Domestics**	**Domestics**	**Wilds**	**Ancient+Modern Wilds**
**Single Wave**	0	0	0.178	0.267
**Two Waves**	0.164	0.139	0.697	0.870

Table S16. AMOVA/Arlequin pairwise F_{ST} measures for mtDNA data. Below the diagonal: F_{ST} estimates. Above the diagonal: p-value estimates. Significant ($p<0.05$) F_{ST} values are indicated in red. Turkmen = Turkmenistan, W. = West, E. = East; Abdul = Tepe Abdul Hosein.

	**Levant**	**W. Turkey**	**Serbia**	**Bulgaria**	**Turkmen.**	**E. Iran**	**Abdul**	**Ganj Dareh**
**Levant**	0	0.00812+-0.0009	0.02624+-0.0015	0.02544+-0.0013	0.02861+-0.0019	0.00277+-0.0005	0.00416+-0.0006	0.00010+-0.0001
**W. Turkey**	0.76872	0	0.25552+-0.0048	0.44362+-0.0047	0.15216+-0.0034	0.00663+-0.0007	0.06851+-0.0026	0.26463+-0.0041
**Serbia**	0.88837	0.17892	0	0.45392+-0.0056	0.03069+-0.0017	0.00317+-0.0006	0.02851+-0.0018	0.20731+-0.0039
**Bulgaria**	0.86188	0.05692	-0.05405	0	0.09435+-0.0026	0.06702+-0.0026	0.09148+-0.0031	0.51619+-0.0049
**Turkmen.**	0.82267	0.22873	0.54062	0.41954	0	0.84506+-0.0037	0.07395+-0.0027	0.34422+-0.0045
**E. Iran**	0.83008	0.25867	0.32815	0.22458	-0.1983	0	0.04257+-0.0020	0.20048+-0.0040
**Abdul**	0.82155	0.19813	0.27996	0.1778	0.15272	0.14608	0	0.56608+-0.0049
**Ganj Dareh**	0.7178	0.02691	0.02871	-0.06279	-0.01212	0.02941	-0.02496	0

Table S17. Y chromosome contig called sites for ancient samples. Haplogroups are assigned based on clade position with respect to modern sequences, typed at diagnostic positions.

Sample	Sites Called - Transversion only	Sites Called - All Sites	Haplogroup
Abdul1	32	89	Y1AA
Abdul2	48	130	Y1AA
Abdul6	22	59	Y1AA
Acem2	426	1097	Y2A
Azer4	200	525	Y2A
Blagotin1	446	1183	Y1B
Blagotin16	354	968	Y1AA
Blagotin3	502	1329	Y1AA
Bulak2	160	423	Y2B
Chalow1	14	23	Y1B
Direkli5	10	37	Y2A
Direkli6	238	647	Basal/Y2
Ganjdareh20	318	842	Y1AA
Ganjdareh22	314	844	Y1AA
Ganjdareh24	15	52	Y1
Ganjdareh34	152	402	Y1AA
Monjukli8	186	478	Y2A
Semnan10	92	240	Y2B
Semnan7	345	929	Y1AB
Semnan9	202	538	Y2B

Table S18. AMOVA/Arlequin pairwise differences for mtDNA data. Above the diagonal: average number of pairwise differences between populations (Pi_{XY}), diagonal elements: average number of pairwise differences within population (Pi_X); below diagonal: corrected average pairwise difference (Pi_{XY}-(Pi_X+Pi_Y)/2). Significant ($p<0.05$) pairwise values are indicated in red. Turkmen = Turkmenistan, W. = West, E. = East; Abdul = Tepe Abdul Hosein.

	Levant	W. Turkey	Serbia	Bulgaria	Turkmen.	E. Iran	Abdul	Ganj Dareh
**Levant**	45.33	205.40	213.50	203.50	221.83	221.88	222.08	215.42
**W. Turkey**	158.13	49.20	33.45	31.73	54.13	54.83	52.70	59.83
**Serbia**	189.67	7.68	2.33	2.50	31.17	32.38	29.83	41.83
**Bulgaria**	179.33	5.63	-0.17	3.00	29.00	30.00	28.39	39.56
**Turkmen.**	183.83	14.20	14.67	12.17	30.67	27.25	40.06	51.97
**E. Iran**	182.17	13.19	14.17	11.46	-5.12	34.07	40.96	52.33
**Abdul**	181.42	10.10	10.67	8.89	6.72	5.92	36.00	50.65
**Ganj Dareh**	160.50	2.98	8.42	5.81	4.39	3.05	0.40	64.50

Table S19. Genes overlapping F_{ST}/θ and PBS outlier windows.

Fst/Theta (overlapping)	Fst/Theta (nearest)	PBS	PBS	PBS
ACSM1	USP25	ABCC2	LOC102170238	LRRC58
AK3	CADM2	ANO6	LOC102171420	LRRC75A
CNOT11	ZNF804A	ARHGEF40	LOC102172038	MC5R
HOOK1	ENSCHIG00000009122	ATG4C	LOC102172204	MSMP
IBTK	KYNU	C16H1orf101	LOC102172897	NDRG2
ITGB1	ENSCHIG00000025678	C2H2orf76	LOC102173333	NPR2
LOC102168221	DUSP6	CADPS2	LOC102174011	NRDC
LOC102169730	ENSCHIG00000006327	CAMK4	LOC102174190	NSMCE1
LOC102174011	TECRL	CCT8	LOC102174212	PLD1
LOC102175877	CSNK1G3	CD5L	LOC102174724	PPP2R5E
LOC102175925	ENSCHIG00000012586	CEP350	LOC102175300	QSOX1
LOC102179637	KLHL1	CP	RNASE4	RAD21L1
LOC102180092	ENSCHIG00000023099	CREB3	LOC102179751	RERE
LOC102181592	ENSCHIG00000020857	DBI	LOC102180092	RGP1
LOC102184244	GABRB3	DENND5B	LOC102183415	RNASE13
LOC102186455	ENSCHIG00000024628	DESI2	LOC102183637	RNASE6
LOC102191034	ENSCHIG00000007130	EDDM3B	LOC102186680	RNMT
LOC102191785	ENSCHIG00000022394	ETFBKMT	LOC102190523	RWDD2B
LOC108633231		FAM210A	LOC102191011	SLC39A2
LOC108635665		FSTL1	LOC102191030	TCF12
LOC108636093		GBA2	LOC102191297	TMC3
LOC108638013		GPHN	LOC102191590	TMEM268
MUCL1		HNF4G	LOC106502442	TNFSF15
NAALADL2		HPS1	LOC106502530	TPPP2
NUP54		HPS3	LOC108635665	TRPV2
RCL1		HPSE2	LOC108636433	TSPAN9
RNF149		IBTK	LOC108636854	USP16
SCARB2		KDM8	LOC108636955	
TSPAN9				
ZNF641				

Legend for Dataset S1: All *D* statistics tests performed. |Z| of 3 was taken as significant. Analyses were performed on transversion variants only.

SI References

1. P. E. L. Smith, Ganj Dareh Tepe. *Paléorient* **2**, 207–209 (1974).

2. J. Boessneck, "Osteological differences between sheep (Ovis aries Linné) and goat (Capra hircus Linné)" in *Science in Archaeology: A Comprehensive Survey of Progress and Research*, D. R. Brothwell, E. S. Higgs, Eds. (Thames and Hudson, 1969), pp. 331–358.

3. S. Payne, "A metrical distinction between sheep and goat metacarpals" in *The Domestication and Exploitation of Plants*, P. J. Ucko, G. W. Dimbleby, Eds. (Duckworth, 1969), pp. 295–305.

4. S. Payne, Morphological distinctions between the mandibular teeth of young sheep, Ovis, and goats, Capra. *J. Archaeol. Sci.* **12**, 139–147 (1985).

5. D. Helmer, M. Rocheteau, *Atlas de squelette appendiculaire des principaux genres holocenes de petits ruminants du nord de la Mediterranee et du proche-Orient: (Capra, Ovis, Rupicapra, Capreolus, Gazella)* (APDCA, 1994).

6. D. Helmer, Discrimination des genres Ovis et Capra à l'aide des prémolaires inférieures 3 et 4 et interprétation des âges d'abattage : l'exemple de Dikili Tash (Grèce). *Anthropozoologica* **31** (2000).

7. P. Halstead, P. Collins, V. Isaakidou, Sorting the sheep from the goats: Morphological distinctions between the mandibles and mandibular teeth of AdultOvis and Capra. *J. Archaeol. Sci.* **29**, 545–553 (2002).

8. M. A. Zeder, H. A. Lapham, Assessing the reliability of criteria used to identify postcranial bones in sheep, Ovis, and goats, Capra. *J. Archaeol. Sci.* (2010).

9. M. A. Zeder, "Reconciling rates of long bone fusion and tooth eruption and wear in sheep (Ovis) and goat (Capra)" in *Recent Advances in Ageing and Sexing Animal Bones*, D. Ruscillo, Ed. (Oxbow Books, 2006), pp. 87–118.

10. I. A. Silver, "The Ageing of Domestic Animals" in *Science in Archaeology: A Survey of Progress and Research*, D. R. Brothwell, E. S. Higs, Eds. (Thames & Hudson, 1969), pp. 283–302.

11. A. von den Driesch, *A Guide to the Measurement of Animal Bones from Archaeological Sites* (Peabody Museum Press, 1976).

12. S. Davis, Size variation of the fox, Vulpes vulpes in the palaearctic region today, and in Israel during the late Quaternary. *Journal of Zoology* **182**, 343–351 (1977).

13. S. J. M. Davis, Rabbits and Bergmann's rule: how cold was Portugal during the last glaciation? *Biol. J. Linn. Soc. Lond.* **128**, 526–549 (2019).

14. U. Albarella, K. Dobney, P. Rowley-Conwy, Size and shape of the Eurasian wild boar (Sus scrofa), with a view to the reconstruction of its Holocene history. *Environ. Archaeol.* **14**, 103–136 (2009).

15. M. Zeder, “Animal domestication in the Zagros: an update and directions for future research” in *Archaeozoology of the Near East VIII: Proceedings of the 8th International Symposium on the Archaeozoology of Southwestern Asia and Adjacent Areas*, Travaux de la Maison de l’Orient., Emannuelle Vila, Lionel Gourichon, Alice M. Choyke, and Hijlke Buitenhui, Ed. (Maison de l’Orient et de la Méditerranée, 2008), pp. 243–277.

16. S. Pääbo, *et al.*, Genetic analyses from ancient DNA. *Annu. Rev. Genet.* **38**, 645–679 (2004).

17. S. Boessenkool, *et al.*, Combining bleach and mild pre-digestion improves ancient DNA recovery from bones. *Mol. Ecol. Resour.* (2016) https:/doi.org/10.1111/1755-0998.12623.

18. D. Y. Yang, B. Eng, J. S. Waye, J. C. Dudar, S. R. Saunders, Technical note: improved DNA extraction from ancient bones using silica-based spin columns. *Am. J. Phys. Anthropol.* **105**, 539–543 (1998).

19. D. E. MacHugh, C. J. Edwards, J. F. Bailey, D. R. Bancroft, D. G. Bradley, The extraction and analysis of ancient DNA from bone and teeth: a survey of current methodologies. *Anc. Biomol.* **3**, 81–103 (2000).

20. C. Gamba, *et al.*, Genome flux and stasis in a five millennium transect of European prehistory. *Nat. Commun.* **5**, 5257 (2014).

21. K. G. Daly, *et al.*, Ancient goat genomes reveal mosaic domestication in the Fertile Crescent. *Science* **361**, 85–88 (2018).

22. N. Rohland, E. Harney, S. Mallick, S. Nordenfelt, D. Reich, Partial uracil-DNA-glycosylase treatment for screening of ancient DNA. *Philos. Trans. R. Soc. Lond. B Biol. Sci.* **370**, 20130624 (2015).

23. M. Meyer, M. Kircher, Illumina sequencing library preparation for highly multiplexed target capture and sequencing. *Cold Spring Harb. Protoc.* **2010**, db.prot5448 (2010).

24. S. W. Wingett, S. Andrews, FastQ Screen: A tool for multi-genome mapping and quality control. *F1000Res.* **7** (2018).

25. H. Li, J. Ruan, R. Durbin, Mapping short DNA sequencing reads and calling variants using mapping quality scores. *Genome Res.* **18**, 1851–1858 (2008).

26. D. M. Bickhart, *et al.*, Single-molecule sequencing and chromatin conformation capture enable de novo reference assembly of the domestic goat genome. *Nat. Genet.* **49**, 643–650 (2017).

27. H. Jónsson, A. Ginolhac, M. Schubert, P. L. F. Johnson, L. Orlando, mapDamage2.0: fast approximate Bayesian estimates of ancient DNA damage parameters. *Bioinformatics* **29**, 1682–1684 (2013).

28. A. Gnirke, *et al.*, Solution hybrid selection with ultra-long oligonucleotides for massively parallel targeted sequencing. *Nat. Biotechnol.* **27**, 182–189 (2009).

29. T. Maricic, M. Whitten, S. Pääbo, Multiplexed DNA sequence capture of mitochondrial

genomes using PCR products. *PLoS One* **5**, e14004 (2010).

30. N. J. O'Sullivan, *et al.*, A whole mitochondria analysis of the Tyrolean Iceman's leather provides insights into the animal sources of Copper Age clothing. *Sci. Rep.* **6**, 31279 (2016).

31. M. Martin, Cutadapt removes adapter sequences from high-throughput sequencing reads. *EMBnet.journal* **17**, 10–12 (2011).

32. M. Meyer, *et al.*, A high-coverage genome sequence from an archaic Denisovan individual. *Science* **338**, 222–226 (2012).

33. H. Li, *et al.*, The Sequence Alignment/Map format and SAMtools. *Bioinformatics* **25**, 2078–2079 (2009).

34. Z. Zheng, *et al.*, The origin of domestication genes in goats. *Sci Adv* **6**, eaaz5216 (2020).

35. M. Schubert, S. Lindgreen, L. Orlando, AdapterRemoval v2: rapid adapter trimming, identification, and read merging. *BMC Res. Notes* **9**, 88 (2016).

36. A. McKenna, *et al.*, The Genome Analysis Toolkit: a MapReduce framework for analyzing next-generation DNA sequencing data. *Genome Res.* **20**, 1297–1303 (2010).

37. A. Hassanin, C. Bonillo, B. X. Nguyen, C. Cruaud, Comparisons between mitochondrial genomes of domestic goat (Capra hircus) reveal the presence of numts and multiple sequencing errors. *Mitochondrial DNA* **21**, 68–76 (2010).

38. T. S. Korneliussen, A. Albrechtsen, R. Nielsen, ANGSD: Analysis of Next Generation Sequencing Data. *BMC Bioinformatics* **15**, 356 (2014).

39. H. Li, Aligning sequence reads, clone sequences and assembly contigs with BWA-MEM. *arXiv [q-bio.GN]* (2013).

40. The Broad Institute, *Picard Tools* (The Broad Institute, 2018) (May 10, 2018).

41. L. Colli, *et al.*, Whole mitochondrial genomes unveil the impact of domestication on goat matrilineal variability. *BMC Genomics* **16**, 1115 (2015).

42. R. C. Edgar, MUSCLE: multiple sequence alignment with high accuracy and high throughput. *Nucleic Acids Res.* **32**, 1792–1797 (2004).

43. S. Guindon, *et al.*, New algorithms and methods to estimate maximum-likelihood phylogenies: assessing the performance of PhyML 3.0. *Syst. Biol.* **59**, 307–321 (2010).

44. V. Lefort, J.-E. Longueville, O. Gascuel, SMS: Smart Model Selection in PhyML. *Mol. Biol. Evol.* **34**, 2422–2424 (2017).

45. A. Rambaut, FigTree (2009) (May 10, 2018).

46. L. Excoffier, H. E. L. Lischer, Arlequin suite ver 3.5: a new series of programs to perform population genetics analyses under Linux and Windows. *Mol. Ecol. Resour.* **10**, 564–567

(2010).

47. R. Bouckaert, *et al.*, BEAST 2.5: An advanced software platform for Bayesian evolutionary analysis. *PLoS Comput. Biol.* **15**, e1006650 (2019).

48. R. R. Bouckaert, A. J. Drummond, bModelTest: Bayesian phylogenetic site model averaging and model comparison. *BMC Evol. Biol.* **17**, 42 (2017).

49. A. Rambaut, A. J. Drummond, D. Xie, G. Baele, M. A. Suchard, Posterior Summarization in Bayesian Phylogenetics Using Tracer 1.7. *Syst. Biol.* **67**, 901–904 (2018).

50. Y. Jiang, *et al.*, The sheep genome illuminates biology of the rumen and lipid metabolism. *Science* **344**, 1168–1173 (2014).

51. J. Meisner, A. Albrechtsen, Inferring Population Structure and Admixture Proportions in Low-Depth NGS Data. *Genetics* **210**, 719–731 (2018).

52. D. Lawson, L. van Dorp, D. Falush, A tutorial on how (not) to over-interpret STRUCTURE/ADMIXTURE bar plots. *bioRxiv*, 066431 (2017).

53. E. Paradis, K. Schliep, ape 5.0: an environment for modern phylogenetics and evolutionary analyses in R. *Bioinformatics* **35**, 526–528 (2019).

54. A. Stamatakis, RAxML version 8: a tool for phylogenetic analysis and post-analysis of large phylogenies. *Bioinformatics* **30**, 1312–1313 (2014).

55. P. Danecek, *et al.*, The variant call format and VCFtools. *Bioinformatics* **27**, 2156–2158 (2011).

56. S. Purcell, *et al.*, PLINK: a tool set for whole-genome association and population-based linkage analyses. *Am. J. Hum. Genet.* **81**, 559–575 (2007).

57. J. K. Pickrell, J. K. Pritchard, Inference of population splits and mixtures from genome-wide allele frequency data. *PLoS Genet.* **8**, e1002967 (2012).

58. J. Felsenstein, PHYLIP: Phylogeny Inference Package. Version 3.2. *Cladistics*, 164–166 (1989).

59. B. Harrington, *Inkscape*.

60. N. Patterson, *et al.*, Ancient admixture in human history. *Genetics* **192**, 1065–1093 (2012).

61. G. R. Warnes, *et al.*, *gplots: Various R Programming Tools for Plotting Data* (2019).

62. É. Harney, N. Patterson, D. Reich, J. Wakeley, Assessing the Performance of qpAdm: A Statistical Tool for Studying Population Admixture. *Cold Spring Harbor Laboratory*, 2020.04.09.032664 (2020).

63. M. Ní Leathlobhair, *et al.*, The evolutionary history of dogs in the Americas. *Science* **361**, 81–85 (2018).

64. L. Liu, *et al.*, Genomic analysis on pygmy hog reveals extensive interbreeding during wild

boar expansion. *Nat. Commun.* **10**, 1992 (2019).

65. S. D. E. Park, *et al.*, Genome sequencing of the extinct Eurasian wild aurochs, Bos primigenius, illuminates the phylogeography and evolution of cattle. *Genome Biol.* **16**, 234 (2015).

66. T. van der Valk, D. Díez-Del-Molino, T. Marques-Bonet, K. Guschanski, L. Dalén, Historical Genomes Reveal the Genomic Consequences of Recent Population Decline in Eastern Gorillas. *Curr. Biol.* **29**, 165–170.e6 (2019).

67. M. de Manuel, *et al.*, The evolutionary history of extinct and living lions. *Proc. Natl. Acad. Sci. U. S. A.* **117**, 10927–10934 (2020).

68. H. Wickham, *ggplot2: Elegant Graphics for Data Analysis* (Springer-Verlag, 2016) (March 30, 2021).

69. F. C. Ceballos, P. K. Joshi, D. W. Clark, M. Ramsay, J. F. Wilson, Runs of homozygosity: windows into population history and trait architecture. *Nat. Rev. Genet.* **19**, 220–234 (2018).

70. G. Renaud, K. Hanghøj, T. S. Korneliussen, E. Willerslev, L. Orlando, Joint Estimates of Heterozygosity and Runs of Homozygosity for Modern and Ancient Samples. *Genetics* (2019) https:/doi.org/10.1534/genetics.119.302057.

71. H. Ringbauer, J. Novembre, M. Steinruecken, Human Parental Relatedness through Time - Detecting Runs of Homozygosity in Ancient DNA. 2020.05.31.126912 (2020).

72. I. J. Nijman, *et al.*, Phylogeny and distribution of Y-chromosomal haplotypes in domestic, ancient and wild goats. *biorxiv*, 2020.02.17.952051 (2020).

73. M. Lipatov, K. Sanjeev, R. Patro, K. Veeramah, Maximum Likelihood Estimation of Biological Relatedness from Low Coverage Sequencing Data. *bioRxiv*, 023374 (2015).

74. U. Raudvere, *et al.*, g:Profiler: a web server for functional enrichment analysis and conversions of gene lists (2019 update). *Nucleic Acids Res.* **47**, W191–W198 (2019).

75. J. T. Robinson, *et al.*, Integrative genomics viewer. *Nat. Biotechnol.* **29**, 24–26 (2011).

76. C. B. Ramsey, Bayesian Analysis of Radiocarbon Dates. *Radiocarbon* **51**, 337–360 (2009).

77. P. J. Reimer, *et al.*, The IntCal20 Northern Hemisphere Radiocarbon Age Calibration Curve (0–55 cal kBP). *Radiocarbon*, 1–33 (2020).

78. K. V. Flannery, “Hunting and early animal domestication at Tepe Guran” in *Excavations at Tepe Guran: The Neolithic Period*, Acta Iranica., P. Mortensen, Ed. (Peeters, 2014), pp. 89–105.

79. M. Rosenberg, H. Togul, The Batman River archaeological site survey, 1990. *Anatolica* (1991).

80. H. E. Wright, The Geological Setting of Four Prehistoric Sites in Northeastern Iraq. *Bull.*

Am. Schools Orient. Res., 11–24 (1952).

81. B. L. Peasnall, M. Rosenberg, “A preliminary description of the lithic industry from Demirköy Höyük” in *Beyond Tools*, I. Caneva, C. Lemorini, D. Zampetti, P. Biagi, Eds. (ex oriente, 2001), pp. 363–387.

82. R. J. Braidwood, L. Braidwood, Jarmo: A Village Early Farmers in Iraq. *Antiquity* **24**, 189–195 (1950).

83. V. Özkaya, O. San, H. Yildizhan, “Excavations at Körtik Tepe: 2000” in *Salvage Project of the Archaeological Heritage of the Ilisu and Carchemish Dam Reservoirs: Activities in 2000*, N. Tuna, J. Velibeyoğlu, Eds. (Orta Doğu Teknik Üniversitesi, 2002), pp. 739–758.

84. W. Matthews, L. M. Shillito, S. Elliott, “Investigating Early Neolithic materials, ecology and sedentism: micromorphology and microstratigraphy” in *The Earliest Neolithic of Iran: 2008 Excavations at Sheikh-E Abad and Jani, Central Zagos Archaeological Project, Volume 1*, British Institute of Persian Studies, Archaeological Monograph Series., R. Matthews, W. Matthews, Y. Mohammadifar, Eds. (Oxbow Books, 2008), pp. 64–108.

85. Y. Miyake, O. Maeda, K. Tanno, H. Hongo, C. Y. Gündem, New excavations at Hasankeyf Höyük: A tenth millennium cal. BC site on the Upper Tigris, southeast Anatolia. *Neo-Lithics* **12**, 3–7 (2012).

86. R. J. Braidwood, B. Howe, E. O. Negahban, Near Eastern Prehistory. *Science* **131**, 1536–1541 (1960).

87. R. J. Braidwood, B. Howe, C. A. Reed, The Iranian Prehistoric Project. *Science* **133**, 2008–2010 (1961).

88. G. Algaze, R. Breuninger, C. Lightfoot, M. Rosenberg, The Tigris-Euphrates Archaeological Reconnaissance Project: A Preliminary Report of the 1989-1990 Seasons. *Anatolica* **XVII**, 175–240 (1991).

89. P. Mortensen, *Excavations at Tepe Guran: The Neolithic Period*, 1st Ed. (Leuven, 2014).

90. R. S. Solecki, A Paleolithic Site in the Zagros Mountains of Northern Iraq, Report on a Sounding at Shanidar Cave, Part 1. *Sumer* **8**, 127–161 (1952).

91. R. L. Solecki, *Zawi Chemi Shanidar, a postpleistocene village site in Northern Iraq* (1964).

92. R. Matthews, W. Matthews, Y. Mohammadifar, *The earliest Neolithic of Iran: 2008 excavations at Sheikh-e Abad and Jani* (Oxbow Books, 2013).

93. S. K. Kozlowski, Nemrik 9, a PPN Neolithic site in Northern Iraq. *Paléorient* **15**, 25–31 (1989).

94. N. J. Conard, S. Riehl, M. Zeidi, “Revisiting Neolithisation in the Zagros Foothills: Excavations at Chogha Golan, Pre-Pottery Neolithic site in Ilam Province, Western Iran” in *The Neolithisation of Iran: The Formation of New Societies*, BANEA monograph Series ., R. Matthews, H. Fazeli Nashli, Eds. (Oxbow Books, 2013), pp. 76–83.

95. R. J. Braidwood, B. Howe, *Prehistoric Investigations in Iraqi Kurdistan*, R. J. Braidwood, B. Howe, Eds. (The University of Chicago Press, 1960).

96. H. Darabi, R. Naseri, R. Young, H. Fazeli, The absolute chronology of East Chia Sabz: a Pre-Pottery Neolithic site in Western Iran. *Doc. Praehist.* **38**, 255 (2011).

97. H. Darabi, R. Naseri, S. Riehl, R. Young, "The Neolithisation process in the Seimarreh Valley: excavations at East Chia Sabz, Central Zagros" in *The Neolithisation of Iran: The Formation of New Societies*, BANEA monograph Series ., R. Matthews, H. Fazeli Nashli, Eds. (Oxbow Books, 2013), pp. 55–75.

98. M. Dittemore, "The soundings at M'lefaat" in *Prehistoric Archaeology along the Zagros Flanks*, L. S. Braidwood, R. J. Braidwood, B. Howe, C. A. Reed, P. J. Watson, Eds. (Oriental Institute of the University of Chicago, 1983), pp. 671–692.

99. B. Moradi, *et al.*, "A short account on Kelek Asad Morad, a Pre-Pottery Neolithic site in Pol e Dokhtar - Luristan" in *The Neolithic of the Iranian Plateau. Recent Research and Prospects*, Studies in Early Near Eastern Production, Subsistence, and Environment., K. Roustaei, M. Mashkour, Eds. (ex oriente, 2016), pp. 1–14.

100. T. Watkins, D. Baird, A. Betts, Qermez dere and the early aceramic neolithic of N. iraq. *Paléorient* **15**, 19–24 (1989).

101. F. Hole, K. V. Flannery, J. A. Neely, *Prehistory and Human Ecology of the Deh Luran Plain: An Early Village Sequence from Khuzistan, Iran* (University of Michigan Press, 1969).

102. E. A. Speiser, Southern Kurdistan in the Annals of Ashurnasirpal and Today. *The Annual of the American Schools of Oriental Research* **8**, 1–41 (1926).

103. A. Alizadeh, *Excavations at the prehistoric mound of Chogha Bonut, Khuzestan, Iran: Seasons 1976/77, 1977/78, and 1996*, Illustrated edition (Oriental Institute of the University of Chicago, 2003).

104. R. J. Braidwood, L. Braidwood, J. G. Smith, C. Leslie, Matarrah: A Southern Variant of the Hassunan Assemblage, Excavated in 1948. *J. Near East. Stud.* **11**, 1–75 (1952).

105. M. A. Zeder, A Metrical Analysis of a Collection of Modern Goats (Capra hircus aegargus and C. h. hircus) from Iran and Iraq: Implications for the Study of Caprine Domestication. *Journal of Archaeological Science* **28**, 61–79 (2001).

106. H. Darabi, T. Richter, P. Mortensen, Neolithization Process in the central Zagros: Asiab and Ganj Dareh revisited. *Documenta Praehistorica* **46**, 44–57 (2019).

107. H. F. Nashli, R. Matthews, "The Neolithisation of Iran: patterns of change and continuity" in *The Neolithisation of Iran: The Formation of New Societies*, H. F. Nashli, R. Matthews, Eds. (Oxbow Books, 2013), pp. 1–13.
